# Overview of Side-Effects of Antibacterial Fluoroquinolones: New Drugs versus Old Drugs, a Step Forward in the Safety Profile?

**DOI:** 10.3390/pharmaceutics15030804

**Published:** 2023-03-01

**Authors:** Aura Rusu, Alexandra-Cristina Munteanu, Eliza-Mihaela Arbănași, Valentina Uivarosi

**Affiliations:** 1 Pharmaceutical and Therapeutical Chemistry Department, Faculty of Pharmacy, George Emil Palade University of Medicine, Pharmacy, Science, and Technology of Targu Mures, 540142 Targu Mures, Romania; 2 Department of General and Inorganic Chemistry, Faculty of Pharmacy, Carol Davila University of Medicine and Pharmacy, 6 Traian Vuia Str., 020956 Bucharest, Romania

**Keywords:** fluoroquinolones, antibacterial quinolones, adverse effects, side-effects, delafloxacin, lascufloxacin, levonadifloxacin, nemonoxacin, sitafloxacin, zabofloxacin

## Abstract

Antibacterial fluoroquinolones (FQs) are frequently used in treating infections. However, the value of FQs is debatable due to their association with severe adverse effects (AEs). The Food and Drug Administration (FDA) issued safety warnings concerning their side-effects in 2008, followed by the European Medicine Agency (EMA) and regulatory authorities from other countries. Severe AEs associated with some FQs have been reported, leading to their withdrawal from the market. New systemic FQs have been recently approved. The FDA and EMA approved delafloxacin. Additionally, lascufloxacin, levonadifloxacin, nemonoxacin, sitafloxacin, and zabofloxacin were approved in their origin countries. The relevant AEs of FQs and their mechanisms of occurrence have been approached. New systemic FQs present potent antibacterial activity against many resistant bacteria (including resistance to FQs). Generally, in clinical studies, the new FQs were well-tolerated with mild or moderate AEs. All the new FQs approved in the origin countries require more clinical studies to meet FDA or EMA requirements. Post-marketing surveillance will confirm or infirm the known safety profile of these new antibacterial drugs. The main AEs of the FQs class were addressed, highlighting the existing data for the recently approved ones. In addition, the general management of AEs when they occur and the rational use and caution of modern FQs were outlined.

## 1. Introduction

Antibacterial quinolones (QNs) are synthetic compounds that are valuable in fighting bacterial infections. Since most of the compounds currently used in therapy have at least one fluorine atom in their chemical structure, the class is known by the generic name of “fluoroquinolones” (FQs). The relatively simple chemical structure, the mechanism of action, the broad spectrum for the newer generations, and the late occurrence of bacterial resistance compared to other antibiotics ensured the success of this class in therapy [1,2,3,4].

**Figure 1:**
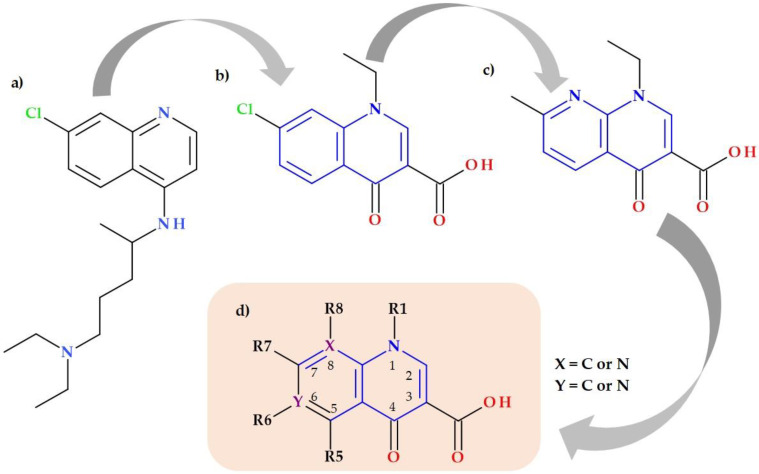
Key compounds in the discovery of QNs: (**a**) chloroquine, (**b**) 7-chloro-l-ethyl-l,4-dihydro-4-oxoquinoline-3-carboxylic acid, (**c**) nalidixic acid, and (**d**) general structure of QNs (QNs—antibacterial quinolones).

The discovery of QNs is closely related to the synthesis of nalidixic acid (an 1,8-naphthyridine derivative) by George Lesher in the early 1960s. The chemical structure of nalidixic acid was based on a secondary product from chloroquine synthesis, an antimalarial drug (Figure 1) [2,5,6].

Nalidixic acid was very efficient in treating urinary tract infections (UTI) due to its activity against Gram-negative bacteria [1]. After the success of the nalidixic acid discovery, new QNs were synthesized, looking for superior pharmacokinetics and a broader antibacterial spectrum. The second generation of QNs comprised compounds with a fluorine substituent at the C6 position (Figure 1), which led to acquiring significant advantages [2,7,8]. The 1-alkyl,1,4-dihydro-4-oxoquinoline-3-carboxylic acid skeleton and the fluorine element are accountable for the efficacy according to structure–activity correlations. Furthermore, the C6-fluorine and C7-piperazinyl substituents are essential for FQs’ broad-spectrum antibacterial. Structural changes at the C7 position led to pharmacokinetic features’ optimization and modification in membrane permeability [9,10,11,12,13].

The leap from one generation to another brought more valuable compounds for treating bacterial infections. Nowadays, the FQs mechanism of action is well-known and reported in detail [14,15,16,17,18]. Two bacterial DNA enzymes (gyrase and topoisomerase IV), involved in the replication and transcription of DNA, are the main targets of FQs from the latest generations [15,19,20]. An updated FQs classification into generations was previously published, and this classification is based on the antibacterial spectrum and therapeutic indications of FQs [4].

In the last decade, the class of FQs acquired valuable representatives. Among them are besifloxacin, finafloxacin, and delafloxacin, new FQs approved by the FDA or EMA [4,21,22]. A broad antibacterial spectrum, including activity against anaerobic bacteria, characterizes these new FQs. An essential advantage is that many resistant bacteria are susceptible to these new drugs [4]. Delafloxacin and finafloxacin present increased antibacterial activity in acidic pH environments [21]. High binding capacity to phosphatidylserine and a superior tissue penetration characterize lascufloxacin developed by Kyorin Pharmaceutical (Japan) [23,24]. Besifloxacin and finafloxacin are topical FQs approved for treating infections with susceptible bacteria [21,25,26,27]. Nemonoxacin is a new non-fluorinated quinolone developed by TaiGen Biotechnology (Taiwan). In December 2013, the FDA granted nemonoxacin “qualified infectious disease product” and “fast-track” designations for community-acquired bacterial pneumonia (CAP) and acute bacterial skin and skin-structure infections (ABSSSI) [28,29,30]. Several advantages characterize the FQs’ fourth generation: some fourth-generation compounds can be classified separately in a new generation (the fifth) according to the spectrum of activity and clinical use [4]. Nowadays, the FQs from the new generations are broad-spectrum antibiotics with excellent pharmacokinetic properties. However, although FQs can treat a wide variety of bacterial infections, their prescription is restricted due to the potential associated severe side-effects. Thus, by the FDA and EMA recommendations, the FQs are not the first-line agents due to their safety profile concerns [31,32,33,34,35].

Since the safety profile of FQs has increasingly concerned clinicians, pharmacists, and researchers, numerous studies have been published, especially in the last two decades. Sometimes, the results of these studies were contradictory or inconclusive. Some reported AEs induced by FQs had been insufficiently studied. Therefore, the drug regulatory authorities formulated numerous recommendations and warnings based on the existing data. This article aims to present a panoramic view of the main AEs induced by FQs and highlight the current safety data about recently approved ones. In addition, the mechanisms underlying these drug-induced AEs were discussed. Additionally, the paper was focused on the general management of AEs when they occur, and the rational use and cautions concerning FQs.

## 2. Materials and Methods

The review is based on references identified with the help of Clarivate Analytics, PubMed, and ScienceDirect databases using the keywords “fluoroquinolones”, “adverse reactions”, and “side-effects”. These keywords have been combined with the name of representative compounds, older and newer FQs. Additionally, the terms of the FQs representatives were combined with the keywords of the severe AEs recorded in the literature, such as “tendinopathy”, “aortic aneurism”, “aortic dissection”, “myasthenia gravis”, “QT prolongation”, “hypoglycemia”, “peripheral neuropathy”, etc. The references were selected if they included relevant data concerning the main topic of our review.

The chemical structures were drawn with Biovia Draw 2019 (https://discover.3ds.com/biovia-draw-academic, accessed on 1 November 2022) [36].

## 3. Relevant FQs Used in Therapy

Over time, the QNs class has acquired numerous compounds. The representatives of the first generation, except nalidixic acid, are not used in therapy anymore (e.g., oxolinic acid, piromidic acid, pipemidic acid, cinoxacin, flumequine), being replaced by more efficient representatives from other generations. Nowadays, some QNs are limited due to poor pharmacokinetic properties or side-effects (e.g., norfloxacin, enrofloxacin). Unfortunately, many promising compounds presented severe AEs and were withdrawn from the market (e.g., sparfloxacin, temafloxacin, grepafloxacin, and clinafloxacin) [4,5,8,37].

### 3.1. The Main FQs with Clinical Importance

The classification into generations based on the spectrum of activity and therapeutic indications is the most used. New compounds are acquired from one generation to another with a broader spectrum of activity (Table 1) and improved pharmacokinetic properties [38]. Among the representatives of the fourth generation are topical FQs (ophthalmic and otic), such as besifloxacin [39] and finafloxacin [21].

New FQs (including a nonfluorinated QN) were approved in India, Japan, South Korea, and Taiwan [4]. These representatives will be addressed in a subsequent section [21,29,40,41].

**Table 1:** QNs and FQs approved by the FDA and EMA from the perspective of the antibacterial spectrum and the main indications (EMA—European Medicine Agency, FDA—USA Food and Drug Administration, FQs—fluoroquinolones, QNs—antibacterial quinolones).

QNs/FQs	1st Generation	2nd Generation	3rd Generation	4th Generation
	Nalidixic Acid	Ciprofloxacin, Nadifloxacin ^1^, Norfloxacin, Ofloxacin, Pefloxacin	Gatifloxacin ^2^, Levofloxacin	Besifloxacin ^2^, Delafloxacin, Finafloxacin ^3^, Moxifloxacin
Antibacterial spectrum	Enterobacteria. No activity against Gram-positive bacteria.	Enterobacteriaceae; some atypical pathogens; *Pseudomonas aeruginosa* (only Ciprofloxacin); some Gram-positive bacteria (including *Streptococcus pneumoniae*), moderate activity against *Staphylococcus aureus* (Ciprofloxacin, Norfloxacin, Ofloxacin, Pefloxacin) *Staphylococcus aureus* ((MRSA) and coagulase-negative staphylococci), aerobic Gram-negative and anaerobic pathogens (Nadifloxacin ^1^)	Broad-spectrum, including *Staphylococcus aureus*, *Streptococcus* species, and Gram-negative pathogens (Gatifloxacin ^2^) Enterobacteriaceae; Atypical pathogens; *Streptococcus pneumoniae,* penicillin-resistant (Levofloxacin)	*Streptococcus pneumoniae*, *Staphylococcus epidermidis, Staphylococcus aureus, Hemophilus influenzae, Moraxella catarrhalis, Corynebacterium* spp. (Besifloxacin ^2^) Broad-spectrum (including methicillin-resistant *Staphylococcus aureus*) (Delafloxacin) Broad-spectrum activity (Finafloxacin ^3^) Enterobacteriaceae; atypical pathogens; *Pseudomonas aeruginosa*; Streptococci; *Staphylococcus aureus* methicillin-sensitive; anaerobic pathogens (Moxifloxacin)
Indications	Uncomplicated urinary tract infections (UTI)	Uncomplicated and complicated UTI, pyelonephritis, sexually transmitted diseases, prostatitis, respiratory tract infections, skin, soft tissues, bones, and joint infections (Ciprofloxacin, Norfloxacin, Ofloxacin, Pefloxacin) Acne vulgaris and other skin infections (Nadifloxacin ^1^).	Bacterial conjunctivitis due to susceptible pathogens (Gatifloxacin ^2^) Acute and chronic bronchitis, exacerbated forms, acquired pneumonia (nosocomial) (Levofloxacin)	Bacterial conjunctivitis (Besifloxacin ^2^) Bacterial skin and skin structure infections (Delafloxacin) Acute otitis externa (Finafloxacin ^3^) Sexually transmitted diseases, prostatitis, skin and tissue infections, acute and chronic bronchitis, exacerbated forms, acquired pneumonia (nosocomial), intra-abdominal infections, and gynecological infections (Moxifloxacin)
References	[2,42]	[1,43]	[44,45,46]	[27,44,47]

^1^ Topical (skin), ^2^ Topical (ophthalmic), ^3^ Topical (otic) administration.

### 3.2. Essential Chemical Characteristics

**Figure 2:**
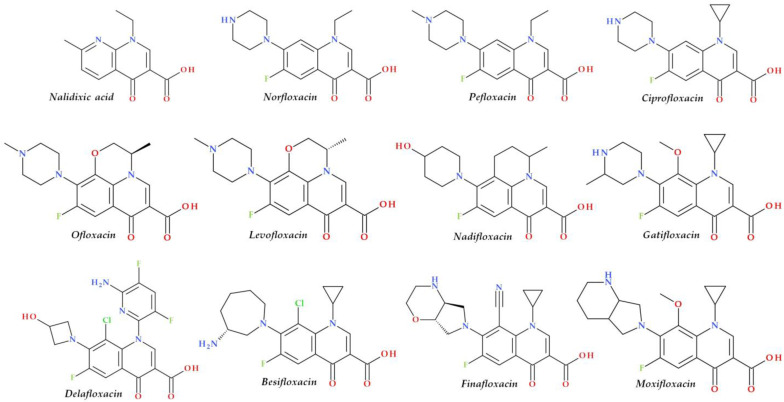
Chemical structures of FQs used in therapy and approved by the EMA and FDA (EMA—European Medicine Agency, FDA—Food and Drug Administration, FQs—fluoroquinolones).

Structural characterization of FQs (older and newer representatives) was recently described in two other papers by our group of authors [4,38]. Essential structural elements of FQs will be briefly highlighted below. FQs are based on quinoline nucleus (ciprofloxacin, norfloxacin, pefloxacin, moxifloxacin, delafloxacin. etc.). Still, some compounds are 1,8-naphthyridine derivatives (e.g., nalidixic acid and zabofloxacin) or tricyclic compounds that include a quinoline nucleus (ofloxacin/levofloxacin, nadifloxacin) (Figure 2) [4,6,8,11].

The N1 position is involved in pharmacokinetic properties and overall potency. Substitution with a cyclopropyl moiety increased the activity against Gram-negative bacteria (e.g., ciprofloxacin). Other substituents were less beneficial or associated with severe AEs (e.g., 2,4-difluorophenyl in temafloxacin) [9,11,48,49]. A 6-amino-3,5-difluoropyridinyl moiety enlarges the delafloxacin’s molecular surface and is responsible for the activity against Gram-positive pathogens [50,51].

The C2 position is optimal without substitution, and a larger substituent may hinder the C3 and C4 positions [9]. A carboxyl group in the C3 position and an oxo(keto) group in the C4 position are essential for interacting with the DNA bases and the enzyme DNA gyrase [9,11,17,52]. Small radicals substituted at the C5 position (e.g., methyl or amino) may increase activity against Gram-positive bacteria, but currently, FQs used in therapy have no substituents in this position [9,11]. The number of halogen substituents on the basic nucleus varies. Many compounds contain a single fluorine atom in the C6 position. The substitution with a fluorine atom increased the potency of the FQs [9,11]. Besifloxacin contains two different halogens in the structure (fluorine in the C6 position and chlorine in the C8 position) [25]. Other representatives have three fluorine atoms (e.g., lascufloxacin) or three fluorine and one chlorine atom (e.g., delafloxacin). Nemonoxacin is a non-fluorinated QN, approved in Taiwan in 2014 [28]. Halogen substitutions lead to increased permeability, decreased solubility, and increased lipophilicity of the compounds [53,54].

**Figure 3:**
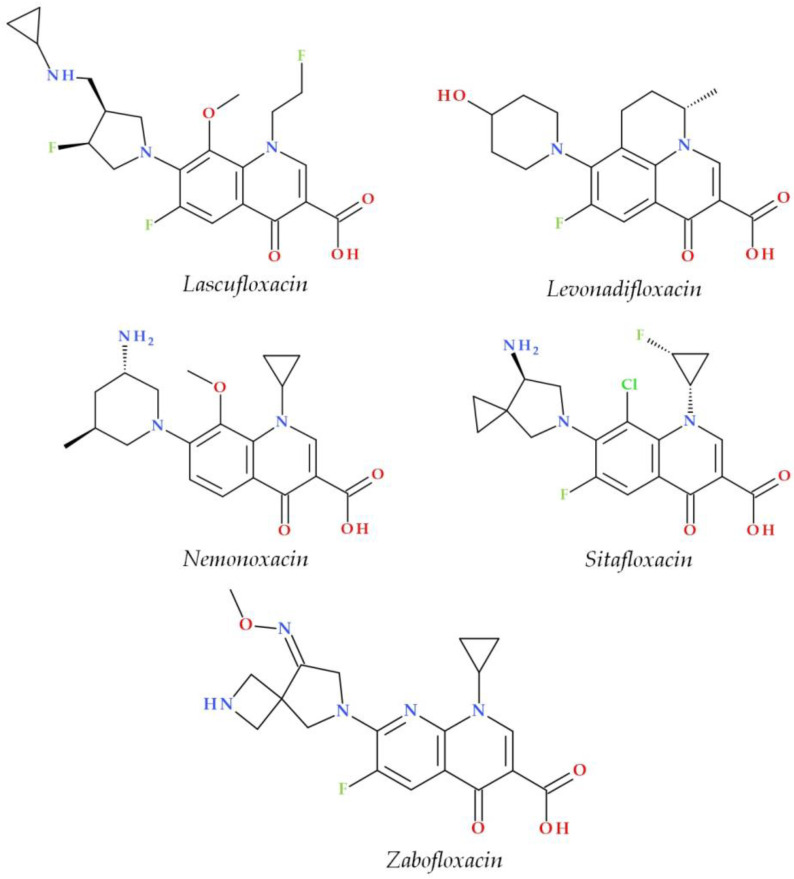
Chemical structures of the recently approved systemic antibacterial (fluoro)quinolones in the countries where they were produced.

The C7 position controls the pharmacokinetics and antibacterial activity of FQs. A five- or six-membered nitrogen heterocycle is optimal, such as piperazine (second-generation FQs), pyrrolo-piperidine (e.g., moxifloxacin), hexahydro-1*H*-azepine (e.g., besifloxacin), 3-hydroxyazetidine (e.g., delafloxacin), and pyrrolo-oxazine (e.g., finafloxacin). The substitution with a piperazine nucleus increased activity against Gram-negative bacteria (especially for the second-generation FQs). Other heterocycles increased activity against Gram-positive bacteria [4,9,48]. In general, the C8 position controls pharmacokinetic properties and activity against anaerobic bacteria. A beneficial C8 substituent is the methoxy group found in moxifloxacin and gatifloxacin and the recent representatives, lascufloxacin and nemonoxacin (Figure 3) [11].

Chlorine substitution at the C8 position increased the antimicrobial potency of besifloxacin by acting on the two target enzymes, DNA gyrase and topoisomerase IV [55]. The C8 cyano group in finafloxacin seems essential in activity against Gram-positive bacteria [21,27].

### 3.3. Mechanism of Action

Many papers concerning the FQs mechanism of action have already been published [3,8,17,18,19,56]. Antibacterial QNs act by inhibiting two enzymes involved in bacterial DNA replication, DNA gyrase and DNA topoisomerase IV. DNA gyrase introduces negative supercoils into DNA, an essential activity for the initiation of DNA replication. Topoisomerase IV removes the interlinking of daughter chromosomes (decatenation) to segregate chromosomes (and plasmids) into daughter cells at the end of a round of replication. The second function of topoisomerase IV (shared with the DNA gyrase) is to relax positive supercoils. In Gram-negative pathogens, the primary target is the DNA gyrase enzyme, while in Gram-positive pathogens, the primary target is the topoisomerase IV enzyme. Thus, the first FQs generations target only the DNA gyrase enzyme from Gram-negative bacteria [17,20,56,57]. Newer FQs representatives target both enzymes from Gram-negative and Gram-positive bacteria [18,58]. Due to FQ-enzyme-DNA complex formation, DNA replication is reversibly inhibited, and the DNA is cleaved in both strands [3,56]. Bacterial death occurs depending on the drug concentration [57]. At low concentrations, FQs block reversible DNA replication and transcription. Next, the inhibition of DNA and RNA synthesis occurs. Thus, the growth of bacteria will be inhibited, but only during FQs therapy [3]. A higher drug concentration (over the minimum inhibitory concentration (MIC)) leads to its binding to the topoisomerase-DNA cleavage complex. Bacterial death depends on the processing of the cleavage complex. The slow death of bacteria arises when the processing of the cleavage complex is missing, and DNA replication and transcription are blocked. Rapid death of bacteria occurs when the cleavage complex is processed, and the broken DNA repair no longer occurs. Due to chromosome fragmentation, the bacterial cell will quickly die [3,59]. These events can produce reactive oxygen species (ROS) and, consequently, more DNA breaks. The DNA damage induced by FQs can be restored with consequences concerning the survival of the bacterial cell [3].

Recent studies highlight the importance of ROS formation and FQs’ lethality [60,61]. It seems that ROS are the dominant factor in FQs’ lethality. ROS accumulation completes the primary DNA damage induced by FQs to kill bacterial cells [61,62]. Numerous studies regarding the increased oxidative stress state generated by the FQs treatment were discussed by Michalak et al. [63].

### 3.4. Safety Warnings concerning Emerging Serious AEs

Although the approved FQs are helpful in treating infections with sensitive germs, a significant disadvantage is the potential risk of associated severe AEs (involving muscles, tendons, or joints and the nervous system) [37,64]. The most reported severe AEs are tendon rupture (especially to the Achilles tendon), arthralgia, tendonitis, pain in extremities, gait disturbance, neuropathies associated with paresthesia, fatigue, memory impairment, depression, sleep disorders, impaired vision, hearing, taste and smell, phototoxicity, genotoxicity, QTc prolongation, hematological effect, hepatic eosinophilia effect, pulmonary interstitial eosinophilia, immunological side-effects, hypoglycemia, and CYP 450 inhibition [8,35]. Due to some severe AEs from those previously listed, many compounds have been withdrawn from therapy (Section 3.5). Thus, FQs are contraindicated in patients who have previously experienced side-effects during treatment with a (fluoro)quinolone antibiotic [35,65]. Since the risks exceed the benefits, the FDA and EMA have recently restricted the use of FQs in treating mild and uncomplicated infections, non-bacterial infections, preventing traveler’s diarrhea, and recurring lower UTI, unless other recommended antibacterial agents cannot be used [32,33,64,65]. Additionally, the FDA and EMA recommended that FQs should not be used as first-line therapies in treating acute sinusitis, bacterial infections among persons with chronic obstructive pulmonary disease (COPD), or UTIs, as the risks outweigh the benefits [33,35].

In 2016, the FDA mandated label revisions for all systemic FQs, along with a Black Box Warning about the possibility of irreversible AEs, including the muscles, joints, tendons, nerves, and central nervous system (CNS), that can occur together in the same patient (Table 2) [66,67]. Additionally, new warnings were made, and other parts of the FQs’ label were updated [66]. In 2018, the list of the approved FQs by the FDA comprised ciprofloxacin, delafloxacin, levofloxacin, gemifloxacin, moxifloxacin, and ofloxacin [68].

On 15 November 2018, the EMA completed an evaluation of significant, debilitating, and possibly irreversible AEs associated with QNs and FQs antibiotics administered orally, injectable, or inhaled, considering the experiences of patients, healthcare workers, and scientists. Following this evaluation, the EMA’s Pharmacovigilance Risk Assessment Committee (PRAC) has suggested that some medications, particularly those containing QNs, should be withdrawn from the market [33,34,35,36,37,38,39,40,41,42,43,44,45,46,47,48,49,50,51,52,53,54,55,56,57,58,59,60,61,62,63,64,65,66,67,68,69,70]. The QNs and FQs subject to these restrictions are: (a) cinoxacin, nalidixic acid, pipemidic acid (QNs), and (b) ciprofloxacin, flumequine, levofloxacin, lomefloxacin, moxifloxacin, norfloxacin, ofloxacin, pefloxacin, prulifloxacin, and rufloxacin (FQs) [34]. Instead, FQs should be used to treat illnesses when an antibiotic is required but other antibiotics are ineffective [70].

**Table 2:** FDA and EMA warnings concerning emerging severe AEs of QNs and FQs (AEs—adverse effects, EMA—European Medicine Agency, FDA—USA Food and Drug Administration, FQs—fluoroquinolones, QNs—antibacterial quinolones, Ref.—references).

No.	Year	Regulatory Entity	Document	Title of Document	Targeted AEs	The Formulations/Administration Concerned	Ref.
1	2008	FDA	FDA alert (8 July 2008)	Information for Healthcare Professionals: Fluoroquinolone Antimicrobial Drugs Black Boxed Warning	Increased risk of tendinitis and tendon rupture	Formulations for systemic use (except ophthalmic or otic formulations)	[71,72,73]
2	2011	FDA	FDA alert (February 2011)	Information for Healthcare Professionals: Fluoroquinolone Antimicrobial Drugs Black Boxed Warning?	Worsening symptoms of patients with myasthenia gravis	Formulations for systemic use	[32,74]
3	2013	FDA	FDA Drug Safety Communication (15 August 2013)	FDA requires label changes to warn of the risk for possibly permanent nerve damage from antibacterial fluoroquinolone drugs taken by mouth or by injection	Side-effects of peripheral neuropathy	Formulations for systemic use except for ophthalmic or otic formulations	[75]
4	2016	FDA	FDA Drug Safety Communication (12 May 2016)	FDA advises restricting fluoroquinolone antibiotic use for certain uncomplicated infections; warns about disabling side-effects that can occur together	Side-effects concerning tendons, muscles, joints, nerves, and CNS	Formulations for systemic use	[76]
5	2016	FDA	FDA Drug Safety Communication (26 July 2016)	FDA updates warnings for oral and injectable fluoroquinolone antibiotics due to disabling side-effects (safety labeling changes)	Side-effects involving nerves, the CNS, tendons, muscles, and joints	Formulations for systemic use	[32]
6	2018	FDA	FDA (10 July 2018)	FDA reinforces safety information about serious low blood sugar levels and mental health side-effects with fluoroquinolone antibiotics; requires label changes (warnings)	Serious risk of blood sugar drop and negative impact on mental health	Formulations for systemic use	[68]
7	2018	FDA	FDA Drug Safety Communication (20 December 2018)	FDA warns about the increased risk of ruptures or tears in the aorta blood vessel with fluoroquinolone antibiotics in certain patients (safety announcement)	Higher risk of aortic dissections or ruptures of an aortic aneurysm	Formulations for systemic use	[77]
8	2018	EMA	EMA/668915/2018 (5 October 2018)	Fluoroquinolone and quinolone antibiotics: PRAC recommends new restrictions on use following a review of disabling potentially long-lasting side-effects available online	Long-term adverse effects affecting tendons, bones, and the nervous system	Formulations for systemic and inhalation route	[34]
9	2019	EMA	EMA/175398/2019 (11 March 2019)	Disabling and potentially permanent side-effects lead to suspension or restrictions of quinolone and fluoroquinolone antibiotics	Side-effects involving the CNS, bones, muscles, joints, and tendons	Formulations for systemic and inhalation route	[35]
10	2020	EMA	EMA/Direct Healthcare Professional Communication (DHPC) (29 November 2020)	DHPC: Systemic and inhaled FQs: risk of heart valve regurgitation/incompetence	Risk of heart valve regurgitation/incompetence	Formulations for systemic and inhalation route	[78]

As a result, the EMA’s PRAC and the FDA recommend restrictions on the prescribing of QNs and FQs due to potentially life-threatening side-effects, such as tendon rupture, musculoskeletal pain, and nerve damage (Table 2) [33]. Additionally, FQs should be contraindicated in patients who have already experienced substantial AEs from a (fluoro)quinolone regimen. FQs should be used with extreme caution in elderly patients, patients with renal illness, and those who have undergone an organ transplant, due to an increased risk of tendon rupture. Additionally, combining FQs and corticosteroids raises the risk of tendon rupture. Therefore, this combination should be avoided [35].

Etminam et al. [79] found that the FQs therapy could be associated with increased aortic and mitral regurgitation. In the same year, a cellular and molecular mechanism was documented concerning FQ-associated aortopathy [80]. Consequently, prescribing advice for specialists has been issued by the EMA and other countries (e.g., the United Kingdom). Systemic or inhaled FQs should be used only after following a rigorous benefit–risk evaluation of different treatments available in the case of individuals at risk for heart valve regurgitation [78,81]. The FDA has not issued any warnings or recommendations regarding increased aortic and mitral regurgitation associated with FQs.

However, in a recent study, Strange et al. [82] demonstrated that increased valvular regurgitation rates are not significantly associated with oral FQs. Therefore, more studies must confirm or deny the link between FQs and increased aortic and mitral regurgitation.

A comprehensive review and meta-analysis published by Tandan et al. [83] evaluated the risk of the common AEs associated with FQs. Additionally, this study compared the occurrence of AEs related to FQs and the occurrence of the AEs associated with other antimicrobial agents. Prescribing FQs led to a significantly higher occurrence of gastrointestinal and CNS side-effects compared to other antimicrobial agents (e.g., macrolides and cefuroxime axetil). However, FQs were associated with fewer gastrointestinal and CNS side-effects versus fosfomycin and the combination of trimethoprim-sulfamethoxazole. In addition, FQs were not related to skin-related AEs [83,84,85].

Regulations from other countries (exclusive of the USA and EU) concerning FQ-associated AEs are discussed below.

Canada. There are five FQs (oral and injectable) approved in Canada by different companies: ciprofloxacin, levofloxacin, moxifloxacin, norfloxacin, and ofloxacin. In 2016, Health Canada changed the labels of oral FQs due to reported cases of patients developing retinal detachment, which has become a significant concern regarding FQs. Health Canada authority emphasizes the necessity of seeing a medical professional if the patients report eye problems during or following the FQs’ administration [86]. In addition, at the beginning of 2017, other safety labeling updates informed Canadians and medical professionals about the possibility of persistent or severe AEs, such as tendinopathy, peripheral neuropathy, and CNS disorders [87].

United Kingdom. Ciprofloxacin, moxifloxacin, levofloxacin, and ofloxacin are the FQs approved for therapy in the United Kingdom. In 2019, the Medicines and Healthcare products Regulatory Agency (MHRA) advised that FQs should not be prescribed to treat non-severe or non-bacterial diseases unless other antibiotics are ineffective. Additionally, following a review of the above-listed severe AEs of these drugs, all FQs were limited, with added health labeling concerns [88]. In 2020, a new safety warning for patients at risk for heart valve regurgitation was announced, stating that FQs should be used only following a rigorous benefit–risk evaluation of other alternative treatments [84].

Australia. Since 1976, Australia has expanded the regulatory standards for antimicrobial treatments among humans, and the use of QNs medications is known for its role as a backup antimicrobial agent. Every three years, a group of experts in infectious diseases evaluates the guidelines for antimicrobial administration in the population and hospitals. FQs are prescribed when necessary or as a better-suited therapy, such as in patients with severe CAP that have acute penicillin hypersensitivity, but in most situations, empirical regimens in national prescription guidelines advise the use of aminoglycosides, β-lactams, or macrolides [89]. There are three approved FQs in Australia: ciprofloxacin, norfloxacin, and moxifloxacin. In 2019, following the public announcements of the FDA and EMA regarding the severe AEs induced by FQs, the Therapeutic Goods Administration (TGA) began researching a rare but severe adverse event of aortic aneurysm associated with FQs [90]. During the TGA’s research, it was also decided to update the labeling of FQs to ensure that all products contain warnings concerning the potential AEs of dysglycemia and adverse mental reactions [91].

### 3.5. Withdrawal of Some FQs over Time

Although some new FQs representatives proved to have good antibacterial activity, a broader activity spectrum, or better pharmacokinetic properties compared to compounds of previous generations, they were associated with severe AEs that led to their withdrawal from the market. Approved FQs that were withdrawn after a few years of approval are alatrofloxacin/trovafloxacin, gatifloxacin, gemifloxacin, grepafloxacin, sparfloxacin, and temafloxacin (Table 3) [64,92].

Severe AEs such as hepatotoxicity, dysglycemia, *Clostridium difficile* infection, fatal arrhythmia due to QT prolongation, and severe hemolytic-uremic syndrome increased the risks of FQs administration and ultimately led to the withdrawal of some FQs on the market all over the world [33]. Only five FQs representatives are approved for systemic use in the USA market (ciprofloxacin, levofloxacin, moxifloxacin, ofloxacin, and delafloxacin) [64]. In 2017, the FQs were in the top 10 topics of the Division of Drug Information (DDI), which is closely connected with the FDA Center for Drug Evaluation and Research (CDER) [120]. In addition, a situation about systemic FQs associated with potential AEs events reported to the DDI in 2013–2017 highlights 2016 as the year with the most significant number of inquiries (703) [121].

## 4. The Modern FQs

### Recently Approved FQs

Several new representatives of FQs have been approved in recent decades. A comprehensive approach to these new drugs was already published [4]. Among these modern FQs are nadifloxacin (2000) [122,123], besifloxacin (2009), finafloxacin (2014), delafloxacin and ozenoxacin (2017), and all are approved by the FDA or EMA [4,124,125,126]. Other new compounds have received approval only in the countries where they were produced (Figure 3). Essential information about modern FQs introduced in therapy is comprised in Table 4.

## 5. Side-Effects of FQs and Underlying Mechanisms

Over time, FQs were associated with severe AEs, such as aortic aneurysm and aortic dissection, tendinopathy/tendon injury, retinal detachment, peripheral neuropathy, neuropsychiatric toxicity, seizures, exacerbation of myasthenia gravis, cutaneous side-effects, phototoxicity, *Clostridium difficile* infection, fatal arrhythmia due to QT prolongation, dysglycemia/hypoglycemia, hepatotoxicity, genotoxicity, severe hemolytic-uremic syndrome, and acute renal failure [33,64,165,166]. Since the risks outweigh the benefits, some FQs have been withdrawn from the market by regulatory authorities or manufacturers (Table 3). In addition, the systemic FQs introduced in therapy before 2000 are restricted and no longer considered first-line antibiotics in treating bacterial infections (Table 2).

However, modern FQs present potent antibacterial activity against many resistant bacteria (including resistance to FQs) and acceptable side-effects [4]. Consequently, these newly approved antibacterial agents must be used judiciously to prevent the rapid development of bacterial resistance [165]. Relationships between the essential elements of FQs chemical structure and the associated side-effects are comprised in Table 5.

The AEs of new systemic FQs reported from existing clinical trials are summarized in Table S1 (Supplementary Materials file). Next, we will briefly highlight the most significant AEs associated with older and newer FQs discussed previously in Section 4, including the mechanisms of occurrence.

### 5.1. Aortic Aneurysm and Aortic Dissection

In 2018, the FDA warned about the increased risk of aortic aneurysm and dissection associated with FQs for systemic use as a “Black Box Warning”. This adverse reaction is scarce, but it is severe due to the dangerous bleeding that threatens the patient’s life [77]. Among the common FQs, ciprofloxacin (2nd generation), levofloxacin (3rd generation), and moxifloxacin (4th generation) were associated with potential aortic aneurysms/dissections and other side-effects [172]. In addition, a higher risk of ruptures or tears in the aorta blood vessel was related to the treatment duration with FQs. A 2.5-fold risk of aortic aneurysm and dissection during FQs treatment was demonstrated by Lee et al. [173].

Thus, a relatively recent retrospective database analysis and self-controlled case series study did not confirm previously published results. Respectively, the observed association between FQs and the aortic aneurysm or dissection were not interpreted as significant. The study included 51,898 patients across three databases from the USA [174]. Additionally, another recent study (nationwide nested case-control) conducted in Denmark did not find a significant association between FQs regimens and the increased rates of valvular regurgitation [82]. The Therapeutic Goods Administration received no reports of aortic aneurysms or dissection associated with FQs regimens from Australia until the date of the FDA warning and the EMA’s PRAC recommendation [90].

#### 5.1.1. Underlying Mechanisms of Aortic Aneurysm and Aortic Dissection

The exact mechanism of FQ-induced ruptures or tears in the aorta blood vessel remains to be clarified [175]. One proposed mechanism claims that FQs upregulate cell matrix metalloproteinases (MMPs). Consequently, collagen fibrils (types I and III) will be reduced [176]. Thus, FQs may interfere with extracellular matrix (collagen and elastic fibers) integrity in the aortas [175]. Disrupted extracellular matrix integrity correlated with impaired biomechanical strength triggers progressive aortic degradation until dissection or rupture [177]. In addition to these data, it was reported that FQs decrease collagen production in mouse tympanic membrane fibroblasts (ciprofloxacin) and tenocytes (human-derived tendon cells) [178,179]. Several other studies have shown that ciprofloxacin enhanced MMPs expression, which mediates collagen and elastic fiber degradation [175]. Additionally, ciprofloxacin was associated with collagen degradation and decreased the inhibitors of the matrix metalloproteins (TIMPs) expression [175,180].

Due to their excellent chelating properties derived from the particular chemical structure, FQs form metal complexes with many ions, including iron, magnesium, and calcium [181,182,183,184]. Therefore, FQs strongly chelate the iron needed by the two essential iron-dependent enzymes in synthesizing collagen, prolyl 4-hydroxylase, and lysyl hydroxylase [185]. In experimental animal models, it has been demonstrated that magnesium [186] and calcium [187] ions involved in collagen synthesis form chelates with FQs during the regimen.

Daneman et al. [188] demonstrated in their population-based longitudinal cohort study (Ontario, Canada) that FQs may contribute to aortic aneurysms. The risk was similar to that of Lee et al. [173]. Less predictable, no significant difference in collagen-associated FQs side-effects was reported in a nationwide cohort study conducted on the pediatric population in Taiwan. This study included six usual systemic FQs: ciprofloxacin, gemifloxacin, levofloxacin, moxifloxacin, norfloxacin, and ofloxacin [189]. In addition, several other studies proved that FQs inhibited cell proliferation and induced cell apoptosis in various cells [175]. Thus, in a study performed on isolated human aortic myofibroblasts, FQs exposure did not significantly influence myofibroblasts’ apoptosis, necrosis, and metabolic viability [80].

#### 5.1.2. Reported Aortic Aneurysm and Aortic Dissection Associated with Modern FQs

So far, none of the recently introduced FQs in therapy have been directly associated with these severe AEs. Thus, the similar chemical structure and the property of complex metal ions are essential elements that contribute to the potential occurrence of these AEs associated with the newly approved representatives.

Delafloxacin, a recently approved drug (2017) [152], was well-tolerated in all three clinical phases [50]. All clinical studies before the approval did not highlight the occurrence of severe aortic aneurysms and dissection side-effects [190,191]. Additionally, the leaflet of delafloxacin includes the boxed warning common to all FQs: tendinitis and tendon rupture, peripheral neuropathy, and CNS effects. In addition, delafloxacin may exacerbate myasthenia gravis [152,192]. Recently, Hornak and Reynoso [193] reported zero side-effects in a retrospective review of five adult patients treated with delafloxacin, which is very encouraging. In patients with CAP, delafloxacin was generally well-tolerated (mild or moderate side-effects) [194]. Post-marketing surveillance will confirm or infirm the known safety profile of delafloxacin [50].

To our knowledge, clinical studies did not report the occurrence of severe aortic aneurysms and dissection AEs for the modern FQs reviewed herein (delafloxacin, lascufloxacin, levonadifloxacin, nemonoxacin, sitafloxacin, and zabofloxacin). In addition, they were generally well-tolerated in clinical studies, with only mild or moderate AEs [50].

### 5.2. Tendinopathy/Tendon Rupture

One of the most severe AEs of FQs therapy is tendinitis and tendon rupture. The injury rarely occurs following the FQs treatment, even if it was stopped for several months [195,196,197]. Before the tendons’ injury, tendinitis is often observed for up to two weeks. In 50% of cases, tendinitis is bilateral [196]. The highest risk of tendinopathy or tendon rupture has been reported for levofloxacin [197,198,199,200,201]. The age of over 60, long-term lung disease, steroid treatment, and impaired renal function are the known risk factors for tendinopathy, and men are more predisposed than women [179,197]. In 2008, the FDA added a “Black Box Warning” to the label of FQs regarding the increased risk for tendonitis and tendon rupture [71,72]. In 2018, the EMA reviewed the severe AEs of systemic or inhaled FQs, including those involving tendons or joints [35].

However, Baik et al. [201] conducted a retrospective observational study on over a million USA senior subjects enrolled in the Medicare insurance program (aged over 65). The results showed that FQs class was not associated with the increased risk of tendon ruptures. Among FQs, ciprofloxacin and moxifloxacin were not associated with any risk for tendon ruptures. Instead, levofloxacin was associated with a significantly increased risk but lower or equal to cephalexin from the cephalosporins class. Thus, the increased activity of MMPs was questioned regarding the observed associations with tendon rupture side-effects.

#### 5.2.1. Underlying Mechanisms of Tendinopathy/Tendon Rupture

The occurrence of tendinitis is explained by the high FQs affinity towards connective tissues (bones and cartilage). Therefore, the FQs concentration in these tissues is higher than the serum concentration, explaining its effectiveness in treating infections of bones and cartilage [196,198]. There are several theories concerning the causes of tendinopathy, such as ischemic influences, a matrix-degrading phenomenon, and toxic changes [189,199,202]. Williams et al. [203] suggested that FQs may alter tendon fibroblast metabolism by stimulating matrix-degrading protease activity. At the same time, FQs may inhibit tendon cell proliferation and the synthesis of matrix ground substance. These suggestions were issued after examining ciprofloxacin’s effect on in vitro fibroblast metabolism from the canine Achilles tendon, paratenon, and shoulder capsule samples. Another mechanism for tendonitis and tendon rupture is based on FQs upregulation of MMPs discussed previously in Section 5.1.1. Consequently, most collagen fibrils (types I and III) found in Achilles’ tendons will be reduced. As a result, the tendon cells will suffer degenerative events (organelle dilatation, vacuole formation, and apoptosis) [97,176,188,189].

Several risk factors and comorbidities that increase the incidence of tendonitis and tendon rupture were identified: age over 60, corticosteroid therapy, chronic lung disease, hemodialysis, transplantation, diabetes mellitus, hypercholesterolemia, gout, hyperparathyroidism (male predominance), rheumatoid arthritis, low bone mineral density associated with osteoporosis, magnesium deficiency, and tendon trauma [99,202].

#### 5.2.2. Tendinopathy/Tendon Rupture Associated with Modern FQs

To the extent of our knowledge, clinical studies did not report treatment-related tendinitis, tendon rupture, or myopathy for the modern FQs reviewed herein.

Delafloxacin. There were no tendinopathy events induced by delafloxacin in clinical trials. Four tendonitis events (mild and moderate in severity) were reported in the phase 3 trial, but all were considered unrelated to delafloxacin therapy [50,191]. Therefore, this rare adverse reaction was deemed unlikely to be detected in the limited phase 2 and 3 studies [204]. However, in an analysis that pooled data from two completed phase 3 studies of delafloxacin for the treatment of ABSSSIs, no patients suffered therapy-related tendinitis, tendon rupture, or myopathy [205,206,207]. Moreover, a randomized, double-blind, global phase 3 study comparing the efficacy and safety of delafloxacin 300 mg twice daily versus moxifloxacin 400 mg once daily in adults with CABP reported that no subject in either group experienced a potential tendon disorder [208].

The printed label of the product Baxdela (approved by the FDA in 2017) includes a warning about tendinitis and tendon rupture (the “Black Box Warnings” common to all FQs) since FQs have been associated with disabling and potentially irreversible severe AEs [152].

### 5.3. Retinal Detachment

Retinal detachment is a severe ocular disease which can lead to permanent vision loss [209]. Large cohort studies reported conflicting results concerning the association between retinal detachment and FQs use [210].

Etminam et al. [211] reported that oral FQs could be associated with a high risk of developing a retinal detachment. Ciprofloxacin was associated with most cases of retinal detachments. The descending order of the cases of administration of FQs related to retinal detachment was: ciprofloxacin (368) > levofloxacin (32) > norfloxacin (22) > moxifloxacin (18) > gatifloxacin (5) (total of 445 cases). Thus, the study did not focus on individual FQs and had some limitations. For example, the study cohort included only ophthalmologic patients, and the risk of retinal detachment associated with FQs was not evaluated in the general population [211]. Pasternak et al. [212] conducted a nationwide cohort study based on the Danish population from 1997 through 2011. The results proved that the therapy with oral FQs was not associated with an increased risk of retinal detachment [212]. Brett [213] published his opinion (editorial) in JAMA about FQs-induced retinal detachment determined. Based on the main published studies to date [211,212], the author considers the absolute risk of FQ-induced retinal detachment relatively low, and the interpretation of the contradictory results is debatable due to study limitations [213]. Eftekhari et al. [214] conducted a retrospective cohort study in the UK (1994–2012 period) and reached a similar conclusion concerning FQ-induced retinal tears and retinal detachment as Pasternak et al. [212].

A meta-analysis by Chui et al. [215] disproved the development of FQ-induced retinal detachment. The study included 695 references, of which 7 observational studies were relevant. The risk of developing retinal detachment associated with FQs regimens was minimal. The authors concluded that retinal detachment would be very rare and associated with FQs treatment. Based on the obtained results, a case-crossover study conducted in France by Raguideau et al. [216] reaffirmed that oral FQs were associated with an increased risk for retinal detachment. The cases also included the rhegmatogenous and exudative types of retinal detachment. Thus, after the publication of the results of Raguideau et al. [216], Douglas et al. [217] expressed concerns about the method of this study in a letter to the JAMA ophthalmology editor. Raguideau et al. [216] replied to the comments and reinforced their previous conclusions.

Using sequence symmetry analysis, a case-only design, and a Korean nationwide healthcare database (2004–2015 period), Baek et al. [218] intended to bring their contribution to the potential relationship between the administration of FQs and retinal detachment. However, the results were not very convincing, suggesting only a possible association. Another study conducted in South Korea (2004–2015) reached similar conclusions [219]. A self-controlled case series study completed in 2018 found no association between FQs and retinal detachment. This study used 3 USA databases, including 40,981 patients (2012–2017), and was pre-registered on clinicaltrials.gov. In conclusion, oral FQs were not associated with an increased risk for retinal detachment before or after the exposure period [174].

In a systematic review, 145 eligible trials were identified by Taher et al. [220] from 1554 original studies concerning FQs, during the 1974–2020 period. No evidence was found regarding FQs regimens associated with retinal detachment in patients with no history of eye diseases. Additionally, Taher et al. [221] identified all retinal detachment reported in the FDA adverse event reporting system (2010–2019 period). Two methods were used for analysis: the proportional reporting ratio and the multi-item gamma Poisson shrinker. Of the targeted FQs, only moxifloxacin showed a positive disproportionality signal for retinal detachment. However, Taher et al. [222] found no increased risk of retinal detachment within 30 days following FQs systemic administration.

#### 5.3.1. Underlying Mechanisms of Retinal Detachment

The mechanism by which FQs therapy could be associated with certainty of retinal detachment is not yet fully elucidated. The connective tissue is essential in eye components, including the retina. Several collagen types keep the retina attached to the choroid. Additionally, the retina contains several layers of different kinds of collagen [188,223]. It is known that FQs interfere with collagen synthesis [178,188]. The reported mechanisms concerning collagen synthesis were previously presented in Section 5.1.1. FQs upregulate MMPs, and consequently, collagen fibrils will be reduced [176]. Thus, FQs may disrupt the extracellular matrix outside the retina [224,225]. The presence of FQs in the vitreous body leads to the impairment of collagen synthesis, which can be related to injury of connective tissues such as the vitreous and vitreous cortex [211].

In addition, an ischemic vascular process may be involved, similar to effects observed in some reported tendinopathies associated with FQs therapy [226].

#### 5.3.2. Retinal Detachment Associated with Modern FQs

To the extent of our knowledge, clinical studies did not report the occurrence of retinal detachment as treatment-emergent AEs for the modern FQs reviewed herein.

### 5.4. Peripheral Neuropathy

Antibiotic therapy can occasionally be associated with peripheral nerve or acute CNS dysfunction [227]. Systemic FQs regimens have been associated with peripheral neuropathy [228,229,230,231,232,233]. For example, a case report of a 20-year-old male with diabetes mellitus type 1 revealed severe painful peripheral neuropathy (10/10 on a numeric scale) following the second day of the levofloxacin regimen for epididymitis. In addition, small fiber neuropathy was found after the skin biopsy [233]. Another case report of a 62-year-old man highlights severe axonal neuropathy after four days of a ciprofloxacin regimen prescribed for urinary infection [231]. Additionally, a 57-year-old Caucasian female developed a painful, debilitating, and permanent peripheral neuropathy associated with ciprofloxacin administration (250 mg once daily for five days) [232]. To Scavone et al. [234], the third generation of FQs are more associated with AEs, including neurological ones. Administration of the usual FQs (ciprofloxacin, levofloxacin, moxifloxacin, norfloxacin, and ofloxacin) was associated with an increased relative incidence of peripheral neuropathy compared to the oral amoxicillin-clavulanate combination. Each additional day of FQ administration increased the risk (by approximately 3%). The risk persisted for up to 180 days following the FQs regimens. However, a recent systematic review conducted by Althaqafi et al. [235] did not obtain relevant data regarding the association between FQs regimens and peripheral neuropathy as an induced side-effect.

In 2013, the FDA required the label update of systemic FQs to better describe peripheral neuropathy as a severe side-effect. Peripheral neuropathy may occur after the first administered FQs doses and may be permanent. At that time, the announcement was addressed to all approved systemic FQs (ciprofloxacin, levofloxacin, gemifloxacin, moxifloxacin, norfloxacin, and ofloxacin) [75].

#### 5.4.1. Underlying Mechanisms of Peripheral Neuropathy

FQs were associated with neurotoxicity through the inhibition of GABA receptors [117,187,195]. Structural elements such as 7-piperazine or pyrrolidinyl moieties without substituents may be essential in interacting with GABA receptors [44,117]. In addition, FQs derivatives with unsubstituted heterocycles in position C7 seem more associated with CNS side-effects [2,117].

#### 5.4.2. Reported Peripheral Neuropathy Associated with Modern FQs

Delafloxacin proved to be a well-tolerated FQ regardless of the administrated formulation (i.v. or oral) and until now was not associated with the increased risk of AEs reported for other FQs [51,204]. Sporadic cases of peripheral neuropathy were, however, reported by some clinical studies. For instance, a phase 3 trial comparing the safety and efficacy of delafloxacin versus the combination therapy vancomycin/aztreonam in the treatment of ABSSSI reported one case of paresthesia in each treatment group that was thought to be potentially related to treatment [51,206].

A randomized clinical trial evaluating delafloxacin compared with the best available therapy in patients with surgical site infections found that 1.49% of the patients in the delafloxacin group (versus 2.27% best available treatment) experienced a headache. At the same time, no cases of paresthesia were reported in either group [236]. Hypoesthesia, burning sensation, and paresthesia were the reported events for potential neuropathy. These registered side-effects were mild or moderate in severity. The rates of possible neuropathy were very close to comparators (phase 3 clinical for ABSSSI studies). Additionally, the rates of events related to delafloxacin treatment were lower than comparators. The incidence of potential peripheral neuropathy induced by delafloxacin lasting longer than 30 days was similar to comparators. There were discontinuations of regimens or severe AEs associated with delafloxacin [191]. However, in another phase 3 study, no subjects reported peripheral neuropathy drug-related adverse events (by the FDA definition of FQs-associated disability) [207,208].

The EMA’s summary of the risk management plan for Quofenix (delafloxacin) informs that, during the clinical trials, the incidence of potential peripheral neuropathy was estimated at 0.8%. In this document, peripheral neuropathy is on the “list of important risks and missing information” [154].

Lascufloxacin. There is a limited number of clinical studies available for lascufloxacin. However, in a phase 3 double-blind comparative study of lascufloxacin versus levofloxacin in patients with CAP, 1 patient in 140 treated with lascufloxacin reported drug-related headache versus 2 in 137 patients in the levofloxacin comparator group [237].

Levonadifloxacin. No cases of peripheral neuropathy associated with levonadifloxacin treatment were reported.

Nemonoxacin. In the few existing studies, treatment with nemonoxacin was associated with some signs of peripheral neuropathy (headache, dizziness, and facial/muscle twitch). These are briefly presented below.

In a multiple-dose trial assessing the safety, tolerability, and pharmacokinetics of oral nemonoxacin in healthy volunteers, the most common drug-related adverse effect determined by the investigator was the headache, which was mild in severity (13.9% in the nemonoxacin group and 10% in the placebo group) [238]. Several neuropathy adverse events associated with nemonoxacin treatment were reported in a double-blind, multicenter study in which 265 patients with mild to moderate CAP were randomized to receive oral nemonoxacin (500 mg and 750 mg groups) or levofloxacin 500 mg once daily for 7 days [239]. A randomized, double-blind, multicenter phase 2 study assessed the efficacy and safety of oral nemonoxacin with oral levofloxacin in treating CAP. Only one patient in the nemonoxacin 750 mg group experienced facial twitch, which resulted in treatment discontinuation. This symptom disappeared after the investigational drug administration was stopped [240].

Three multicenter, randomized, double-blind, parallel, comparative clinical trials conducted in Taiwan, South Africa, and China reported CNS disorders, such as dizziness and headache, which were reported in all treatment groups (nemonoxacin 500 mg, nemonoxacin 750 mg, and levofloxacin 500 mg). One event led to drug discontinuation in the nemonoxacin 750 mg group due to muscle twitching. The drug-related side-effects and side-effects resulting in treatment discontinuation were similar between the different groups [30].

Sitafloxacin. Although no serious adverse events occurred in a nested cohort within a multicenter clinical trial evaluating sitafloxacin against CAP caused by *Streptococcus pneumoniae*, the drug-related headache was reported by 2.08% of the patients in the 100 mg once-daily group and by 4.17% of the subjects in the 50 mg twice-daily group [241].

Zabofloxacin seems to have a safety profile concerning peripheral neuropathy as an adverse effect of its therapy. In a randomized, two-way crossover study comparing a zabofloxacin hydrochloride 400 mg capsule (DW224a, 366.7 mg as zabofloxacin) to a zabofloxacin aspartate 488 mg tablet (DW224aa, 366.5 mg as zabofloxacin), two cases (7%) of nausea and a single case (3%) of somnolence were reported [242]. Moreover, a double-blind, double-dummy, randomized, controlled, phase 3, non-inferiority trial assessing the safety and efficacy of oral zabofloxacin (367 mg once daily for five days) versus oral moxifloxacin (400 mg once daily for seven days) in patients with COPD exacerbation, reported mild cases of nausea (zabofloxacin: 1.7%, moxifloxacin: 2.4%) and dizziness (zabofloxacin: 1.7%, moxifloxacin: 0.6%) [243].

### 5.5. Neuropsychiatric Toxicity

There are few published data about the neuropsychiatric toxicity associated with FQs regimens [33,172]. Some relevant studies are presented below.

In a study by Tomé et al. [244], the neurological and psychiatric AEs associated with QNs were reviewed using PubMed/MEDLINE (search of case reports and case series). The results showed that ciprofloxacin, ofloxacin, and pefloxacin were the FQs most associated with neurological and psychiatric AEs. The most frequently reported psychiatric AEs were mania, insomnia, acute psychosis, and delirium. In addition, the most reported neurological AEs were grand mal convulsion, confused state, convulsions, and myoclonus. Pharmacovigilance investigations should be based on collaboration between investigators, basic scientists, and social networks. As a result of good cooperation, revised product labels describe a new severe adverse reaction, FQ-associated disability (FQAD), as recommended by an FDA advisory committee [245].

Results from a study conducted by Samyde et al. [246] during the 1970–2015 period (608 FQ-related cases) showed an increased reported suicidal behavior induced by FQs compared to other antibiotics. By primary analysis, ciprofloxacin was the most strongly associated with suicidal behavior, followed by levofloxacin, moxifloxacin, and ofloxacin. In the secondary analysis results, ofloxacin was the most associated with increased reporting of depressive disorders. Additionally, ciprofloxacin was the most associated with increased reporting of completed suicide [246]. Another retrospective study was conducted during 2005–2013 by Sellick et al. [247] in the Western New York Veterans Affairs Health System. Six hundred and thirty-one hospitalized veterans received ciprofloxacin, levofloxacin, or moxifloxacin for at least 48 h. The study results pointed out that in this veteran population, the FQs regimens may be associated with delirium/psychosis. Two identified risk factors that may predispose targeted patients receiving FQs to develop delirium or psychosis are (1) coadministration with typical antipsychotics and (2) advanced age (the mean age in the study was 71.5 years).

Bennet et al. [172] succeed in evaluating reports about ciprofloxacin-, levofloxacin-, and moxifloxacin-associated neuropsychiatric toxicities, along with long-term disability and aortic aneurysms/dissections. The FDA and EMA published the reports. Neuropsychiatric toxicity events were not unitary. Thus, these AEs could be observed after one dose of FQs or several months. Scavone et al. [234] concluded in their study conducted in Italy that levofloxacin, moxifloxacin, and pefloxacin had a higher reporting probability observed compared to other FQs (ciprofloxacin, lomefloxacin, ofloxacin, and rufloxacin). However, in this study, the classification in generations of FQs was incorrect, considering published literature on this topic.

#### 5.5.1. Underlying Mechanisms of Neuropsychiatric Toxicity

Usual FQs containing substituents in the C7 position were associated with some CNS reactions (e.g., dizziness, insomnia, and headache). Alkyl > piperazine > pyrrolidinyl groups is the order regarding the increase in CNS side-effects, closely related to GABA receptors’ inhibition [8]. Thus, substituting the C7 positions with a piperazinyl or pyrrolidinyl group was associated with GABA-antagonistic effects [117,187]. On the other hand, unsubstituted C7-piperazine or pyrrolidinyl groups of FQs seem essential in the interactions with GABA receptors [44,117], and these FQs derivatives are more associated with CNS side-effects [2,117]. A review published in 2012 extracted 145 individual case reports from 83 articles in which the FQs were related to neurological (46.9%) and psychiatric (40.7%) side-effects, or both (5.5%). Ciprofloxacin, ofloxacin, and pefloxacin were the FQs most associated with neurological and psychiatric side-effects [244]. Additionally, Sellick et al. [247] conducted a retrospective study on hospitalized veterans (2005–2013) and suggested that FQs may be associated with delirium/psychosis. Even if the precise mechanism was not elucidated, this association could be based on N-methyl-*D-*aspartate (NMDA) agonism and GABA receptor antagonism.

Samyde et al. [246] proposed two mechanisms for FQs-induced suicidal behaviors, such as GABA_A_ antagonism and activation of NMDA receptors. In addition, decreased levels of serotonin, altered microRNA expression, and oxidative stress were proposed as mechanisms. In 2015, the FDA identified a “FQ-associated disability” syndrome (neuropsychiatric toxicity) based on previous reports. In 2018, the label of FQs comprised the description of some mental health side-effects in the “Warnings and Precautions” section (disturbances in attention, agitation, disorientation, nervousness, memory impairment, and delirium) [68,245].

#### 5.5.2. Reported Neuropsychiatric Toxicity Associated with Modern FQs

To the extent of our knowledge, clinical studies did not report the occurrence of neuropsychiatric toxicity for the modern FQs reviewed herein.

### 5.6. Seizures

Over time, antibiotic therapy (including FQs) was associated with seizures as side-effects [168]. In a systematic review conducted by Sutter et al. [171], FQ-induced seizures are proven based on case reports, and the most concerning FQ was ciprofloxacin. Ciprofloxacin was administered to patients with renal or mental disorders, prior seizures, or concurrently with theophylline. Only two reports associated ciprofloxacin therapy with seizures in patients without any comorbidities. Levofloxacin and norfloxacin were rarely associated with seizures, most likely due to their limited CNS penetration. Alatrovafloxacin, gatifloxacin, and moxifloxacin had only one published case report for induced seizures or status epilepticus [109,171].

Following the analysis of two case reports (levofloxacin- and ciprofloxacin-induced seizures), Kushner et al. [248] consider that the risk factors for FQs-induced seizures could be seizure history, electrolyte imbalances, unadjusted doses for renal insufficiency, and concomitant treatment with drugs that decrease the seizure threshold. Co-administration of FQs with certain NSAIDs (e.g., fenbufen, due to the 4-biphenylacetic acid metabolite) or co-administration of ciprofloxacin or enoxacin with theophylline (due to cytochrome CYP1A2 interactions) favor the occurrence of seizures [168].

#### 5.6.1. Underlying Mechanisms of Seizures

The first theories by which seizures occur associated with FQs therapy were the binding of the antibiotic to GABA receptors or N-methyl-D-aspartate receptors (in CNS) [5,109]. The animal models revealed that FQs block the GABA receptors at the level of synaptic space [171]. Thus, the FQs could be considered selective antagonists of GABA_A_ receptors in the CNS, resulting in stimulation [97,249]. The pathophysiological basis of the underlying mechanism of seizures is the R7 substituent that controls the binding of FQs to the GABA receptors in the brain and consecutively inhibits their function [48,98]. By inhibiting GABA receptors, the patient becomes prone to epileptic activity (including seizures) [5,248].

There are some relationships between the chemical structure of FQs and the potential to increase the occurrence of associated seizures. The substituents from the C7 position as piperazine or pyrrolidine moieties (except the substituted analogues, such as methylated analogues) were associated with increased seizure potential. Some analogues with bulky moieties in the C7 position (sparfloxacin, temafloxacin) were less suitable to bind to GABA receptors, most likely due to steric hindrance. A high risk for seizures may appear when FQs structures include these moieties, and these FQs are characterized by increased penetration of the CNS [48,97,98,250,251].

#### 5.6.2. Seizures Associated with Modern FQs

To the extent of our knowledge, clinical studies report insufficient data regarding the seizure induced by the modern FQs reviewed herein.

Delafloxacin. In the phase 2 study, a 53-year-old male in the delafloxacin 450 mg arm experienced a generalized seizure, which was thought to be potentially drug-related. The 53-year-old subject was enrolled in the trial with a left chest wall abscess and had an observed seizure on day three after receiving a dosage of delafloxacin. During the subsequent evaluation, he revealed that he had previously suffered seizures but had never had any medical workup. A computed tomography scan was performed the day following the occurrence, and an electroencephalogram and magnetic resonance imaging around three weeks later were unremarkable. There was no conclusive cause for the seizure, and the investigator considered that the investigational drug caused it. Notably, the 300 mg delafloxacin treatment was the best-tolerated regimen [252].

In phase 3 clinical studies, the patients who received delafloxacin (n = 741) did not experience any seizures or convulsions, and stopping the treatment was unnecessary for any patient due to severe events related to seizures/convulsions [51,253,254]. The investigation comparing the incidence of side-effects between delafloxacin and vancomycin/aztreonam across two phase 3 studies for ABSSSI found no convulsions in the delafloxacin group [51].

For lascufloxacin, levonadifloxacin, nemonoxacin, sitafloxacin, and zabofloxacin, no cases of seizures associated with these new FQs treatments were reported.

### 5.7. Exacerbation of Myasthenia Gravis

The FQs may induce exacerbations of myasthenia gravis due to structural characteristics similar to quinoline derivatives (e.g., quinine/quinidine and chloroquine) that block neurotransmission [255,256]. In 2004, Tintinalli [257] warned about the use of FQs (especially levofloxacin) and the association with the occurrence of a myasthenia gravis crisis based on a case observation and scientific literature at that time. Two years later, Gunduz et al. [258] reported levofloxacin as a trigger for a myasthenic crisis and respiratory depression in a patient with no previous history of myasthenia gravis. In February 2011, the FDA added a box warning of FQs’ risk of myasthenia gravis exacerbations [32]. Afterwards, in a retrospective study, Jones et al. [74] identified 37 cases of myasthenia gravis exacerbation following FQs systemic exposure. The FDA Adverse Event Reporting System (AERS) database and scientific literature were researched. Most identified cases were associated with the use of levofloxacin and ciprofloxacin [74]. Recently, a retrospective cohort study included myasthenia gravis patients who received 894 prescriptions with FQs. In this study, Pham Nguyen et al. [259] could not confirm the FQs’ association with myasthenia gravis exacerbation. Thus, the potential of FQs to cause the worsening of myasthenia gravis should not be excluded, and more studies are needed.

#### 5.7.1. Underlying Mechanisms of Exacerbation of Myasthenia Gravis

Most FQs used in therapy are quinolone derivatives, whose basic structure is similar to other drugs with a quinoline structure (e.g., quinine) [4,52,256]. Additionally, the efficacy of certain quinolines to block neuromuscular transmission in botulinum neurotoxin poisoning was assessed. Chloroquine, amodiaquine (4-aminoquinoline), and quinacrine increased by three times the time required for botulinum type A neurotoxin to block neuromuscular transmission. Based on equimolar effective concentrations, quinacrine > amodiaquine > chloroquine > quinine or quinidine was the rank order of potencies [260]. Based on the study of Sieb [261], norfloxacin, ofloxacin, and pefloxacin block neuromuscular transmission.

#### 5.7.2. Reported Exacerbation of Myasthenia Gravis Associated with Modern FQs

Delafloxacin. The rates of potential myopathy (lasting longer than 30 days) were lower for the patients treated with delafloxacin versus the comparator. Additionally, no severe AEs associated with myopathy or treatment discontinuation were reported [191,204]. In the pivotal phase 3 trials, no cases of myopathy (by the FDA definition of FQ-associated disability) were noted [51]. The label of delafloxacin products comprises the “Black Box Warning” relating to events regarding myasthenia gravis alongside tendinitis and tendon rupture, peripheral neuropathy, and CNS effects [32,152].

For lascufloxacin, levonadifloxacin, levonadifloxacin, nemonoxacin, sitafloxacin, and zabofloxacin, no cases of myasthenia gravis exacerbation associated with these new FQs therapies were reported.

### 5.8. Cutaneous Side-Effects, Hypersensitivity Reactions, Anaphylaxis

Antimicrobial agents are known to be responsible for a series of dermatological AEs. These AEs most often occur during therapy with beta-lactams, sulfonamides, fluoroquinolones, and vancomycin [227,262,263]. The FQs treatment was associated with a low incidence of hypersensitivity reactions (erythema, pruritus, urticaria, rash) [262,264]. Although rare, immunologically caused hypersensitivity reactions related to FQs can be severe and life-threatening due to damage to internal organs and circulating blood cells [265,266]. For example, gemifloxacin (a third-generation FQ with a naphtiridone basic structure) was withdrawn in 2009 due to the association with a severe rash erythematous reaction [117,267,268]. An intense, resistant, and biphasic anaphylactic reaction to gemifloxacin mesylate was described by Yilmaz et al. [269] in a case report of a 60-year-old male who had a regimen with one angiotensin-converting enzyme inhibitor, and α- and β-blockers.

There are two general categories of FQ-induced hypersensitivity reactions: (a) immediate reactions mediated by IgE and (b) delayed reactions (which occur after at least one hour) mediated by T-cells. An uncommon immediate reaction is anaphylaxis, and 4.5% of 333 drug-induced cases were produced by FQs [270,271]. Other cutaneous reactions associated with FQs are phototoxicity, acne, fixed drug eruption, angioedema, erythroderma, erythema multiforme, exanthems, Steven–Johnson syndrome, and toxic epidermal necrolysis [264].

Urticaria, angioedema, and shock are the immediate cutaneous AEs associated with QNs and observed with an increasing frequency by Manfredi et al. [272]. They demonstrated that a type I (IgE-mediated) allergic mechanism is involved in these AEs. Regimens with nine QNs (FQs) were observed. The study included 5000 patients with reported AEs; among them, 55 reported 69 immediate AEs to QNs (FQs). In addition, 62% of the patients had previous exposure to QNs (FQs). The history of the patients was similar, with most of them using only one QN/FQ. The allergic reactions were immediate with rapid onset [272]. Cross-reactivity within the class has also been demonstrated [265,272].

Data collected by Kulthanan et al. [273] in a study conducted in Thailand suggested that each FQ is associated with different types and frequencies of cutaneous AEs. The prevalence of AEs was 0.13%, and the prevalence of cutaneous AEs was 0.09% in 166,736 patients with FQs regimens. Ciprofloxacin was the most associated FQ with cutaneous AEs, with maculopapular rash (39.7%) being the most frequent. A previous history of FQs’ hypersensitivity was observed in thirteen cases (8.6% of cases). Cross-reactivity potential had 15.4% between these cases [273]. Among the usual FQs, moxifloxacin was the most associated with anaphylaxis side-effect [270]. Thus, an experimental animal model on albino mice assessed cutaneous drug reactions (severe skin exfoliation or alopecia) induced by four systemic FQs (ciprofloxacin, levofloxacin, moxifloxacin, and ofloxacin). The maximum number of cutaneous drug reactions was caused by ofloxacin; additionally, the onset of cutaneous reactions was significantly earlier comparative to the rest of the studied FQs. Levofloxacin proved to have the least potential for cutaneous drug reactions in this study [274].

In a review article, Neuman et al. [275] have comprehensively addressed the topic of hypersensitivity to FQs that manifest clinically. Additionally, they classified the reactions to FQs by the organ system involved in the response: anaphylaxis, drug-induced delayed reactions, and hypersensitivity syndrome reactions. Systemic reactions comprise anaphylaxis, FQ-induced delayed reactions, and hypersensitivity syndrome reactions. Numerous organ-specific reactions include cutaneous reactions, alongside hepatic reactions and renal reactions. In addition, they highlight that the lymphocyte toxicity assay could be used to diagnose and monitor the hypersensitive reactions associated with FQs. Li and Bernstein [276] described a 56-year-old non-atopic male who received moxifloxacin to treat a refractory sinus infection. Twenty minutes after the administration, signs of anaphylactic shock appeared, such as shortness of breath, nausea, flushing, and vascular collapse, resulting in myocardial infarction. In addition, cross-reactivity of FQs absence was verified by the Prick skin testing.

Doña et al. [277], in their review, focused on the diagnostic approach of allergic reactions to FQs and the management of these AEs. Unfortunately, a precise diagnosis could be challenging due to a lack of validated diagnostic tests and the pathogenic mechanism slightly deciphered [270,277,278]. Diagnosis of allergic reactions induced by FQs is based on the clinical history and in vivo (skin tests, drug provocation tests) and in vitro tests [270]. Hypersensitivity reactions to FQs can be assessed in vitro by basophil activation tests. However, the sensitivity of these tests is not optimal, and the performance must be optimized individually for each FQ [279]. Furthermore, in some hypersensitivity events, the diagnosis is confirmed or excluded only through the FQs provocation test, which involves certain risks [278,280]. A diagnosis scheme and management of patients with allergic reactions induced by FQs was proposed by Doña et al. [277,280,281].

Unfortunately, conflicting results were published concerning cross-reactivity among the FQs class. In addition, FQ-induced hypersensitivity reactions were not uniform across this antibiotic class [270,277,278,281].

#### 5.8.1. Underlying Mechanism of Cutaneous Side-Effects, Hypersensitivity Reactions, and Anaphylaxis

FQs can induce immediate hypersensitivity reactions mediated by IgE and delayed hypersensitivity reactions mediated by T-cells in about 2–3% of patients [272,275]. Delayed immune responses induced by FQs are mediated by T-cells, which could be an explanation for the frequent cross-reactivity among the FQs class. Additionally, cross-reactivity of IgE among FQs class is frequent [272,275]. The chemical structure gives the predisposition for cross-reactivity of FQs, and respectively, the basic nucleus [265]. In vitro studies show that T-cells recognize a basic structure, whereas IgE recognizes smaller groups, such as side chains or smaller moieties, but with lower affinity [277]. Allergies may influence hypersensitivity to FQs to other drugs, such as beta-lactam antibiotics or neuromuscular blocking agents [277].

To elucidate the mechanism of the FQ-induced anaphylactic reactions, Liu et al. [282] conducted an experimental study using wild-type mice, MrgprB2 knockout mice, and mast cell-deficient W-sash c-kit mutant KitW-sh/W-sh mice to investigate nine FQs in vivo. A mast cell-specific receptor that mediates cell degranulation in anaphylactic reactions is the mas-related G protein-coupled receptor X2 (MRGPRX2). In vitro, with the help of human mast cell line LAD2 and MRGPRX2-expressing HEK293 cells, the mechanism of FQ-induced Ca^2+^ mobilization and mast cell degranulation via MRGPRX2 was studied. Mast cells were activated in a dose-dependent manner by FQs. Degranulation was reduced, and consequently, MRGPRX2 silencing. The results prove that FQ-associated anaphylactic reactions are mediated by mast cells through MRGPRX2 [282].

#### 5.8.2. Cutaneous Side-Effects, Hypersensitivity Reactions, and Anaphylaxis Associated with Modern FQs

Delafloxacin. The analysis compared the incidence of AEs between delafloxacin and vancomycin/aztreonam in two phase 3 studies in ABSSSI patients and found that delafloxacin treatment was associated with 0.9% versus 4.7% for the vancomycin/aztreonam group, for skin and subcutaneous tissue disorders (pruritus, urticaria, dermatitis, rash) [51,191]. Moreover, in a randomized clinical study of delafloxacin powder for solution for infusion of 300 mg or a tablet of 450 mg, administered twice daily for 5 to 14 days, compared to the best available therapy in patients with surgical site infections, pruritus was noted in 2 out of 134 patients (1.49%) [236]. All cases were mild and did not lead to treatment discontinuation.

Lascufloxacin. In a phase 3, a double-blind, comparative study of lascufloxacin versus levofloxacin in patients with CAP, a drug-related rash occurred in 2 out of 140 patients, similar to the levofloxacin comparator group (2 in 137 patients) [237].

Levonadifloxacin. An intrapulmonary pharmacokinetics study following oral administration of alalevonadifloxacin (1000 mg twice daily for five days) to 30 healthy adult subjects reported skin papule as a rare AE associated with alalevonadifloxacin treatment [283].

Nemonoxacin. The most relevant data reported on cutaneous AEs associated with nemonoxacin treatment are presented below.

In a double-blind, ascending single-dose study, 56 healthy subjects (48 males and 8 females) were randomized to receive a placebo or single oral doses of 25, 50, 125, 250, 500, 1000, or 1500 mg of nemonoxacin. Nemonoxacin was well-tolerated up to 1500 mg, and no severe AEs were reported during the study. Contact dermatitis, pruritus, and erythema were the most frequent adverse events. Most patients recovered from the reported AEs without treatment during the observation period, while pruritus was resolved after administering intramuscular diphenhydramine or topical calamine lotion [284].

A multiple-dose study of oral nemonoxacin in healthy participants yielded similar results. Contact dermatitis from ECG electrode application (nemonoxacin 8.3% and placebo 10%), rash (nemonoxacin 8.3% and placebo 0%), and ECG electrode application site pruritus (nemonoxacin 2.8% and placebo 10%) were the most frequently reported adverse events. Notably, treatment was discontinued in one subject in the 750 mg group due to a minor rash [238]. In single and multiple oral dose studies assessing the safety and clinical pharmacokinetics of nemonoxacin in healthy Chinese volunteers, 50% (3/6) of the subjects in the 1000 mg group in the single-dose safety and tolerability study reported pruritus, and a skin rash accompanied 1 case. The mild rash was also noted in two participants (2/12) in the 500 mg group after the last treatment (day 10) in the multiple-dose study, and both recovered spontaneously. Four patients (4/12) in the 750 mg group developed a minor rash while taking the medication, but all recovered with or without antihistamines. All subjects completed the multiple-dose study without interruption [285].

An injection site reaction was the most prevalent AE in a randomized, double-blind, placebo-controlled, dose-escalation safety and tolerability study in 92 individuals. The next most common AE was an erythematous rash with or without pruritus that appeared on the face, neck, or trunk skin during treatment. There were no major or severe AEs reported, and all AEs were mild and temporary [148]. A randomized, placebo- and positive-controlled crossover study in healthy Chinese adults found no cutaneous AEs in the nemonoxacin 500 mg group administered in the fasted condition. In comparison, face flushing (12.5%) and pruritus (10.4%) were reported in the group treated with nemonoxacin 750 mg in the fasted condition. In the fed condition, 8.3% of subjects treated with nemonoxacin 500 mg reported a skin rash [28]. Moreover, in another study evaluating the safety and pharmacokinetic/pharmacodynamic profiles of nemonoxacin conducted in healthy Chinese volunteers following multiple-dose intravenous infusion once daily for ten days, the most common AE in the clinical disorder category was found to be an injection site reaction and rash [286].

Three randomized, double-blind, parallel, comparative clinical trials conducted in Taiwan, South Africa, and China reported rash (nemonoxacin 500 mg: 0.4%, nemonoxacin 750 mg: 0%, levofloxacin 500 mg: 1.3%) as a less common adverse event following nemonoxacin compared to levofloxacin treatment [30].

Sitafloxacin. No cases of cutaneous adverse events associated with sitafloxacin treatment were reported.

Zabofloxacin. No cases of cutaneous adverse events associated with zabofloxacin treatment were reported.

### 5.9. Phototoxicity

Phototoxicity and photoallergy are the two types of photosensitivity associated with FQs therapy. Photoallergic reactions require previous exposure to FQs and are rarely encountered [117]. Phototoxicity events associated with FQs regimens have been known since the first representatives were introduced in therapy. The accumulation of FQs in the skin, followed by the activation by exposure to sunlight, could produce severe damage to the skin [8,117]. It was observed that the halogen (fluorine/chlorine atom) substitution in the C8 position led to an increased risk of phototoxicity. The 8-fluorine substituent proved to have more phototoxic potential than 8-chlorine [5,37,195,287]. Additionally, an amino substituent at the C5 position and a fluorine/chlorine substituent at the C8 position can increase the phototoxic potential of FQs [8]. Some halogenated FQs have been withdrawn due to their phototoxic potential, such as clinafloxacin (8-chloro derivative), and fleroxacin, lomefloxacin, and sparfloxacin (8-fluoro derivatives) (Table 3) [2,44]. The representatives with a naphthyridone structure and an additional third nitrogen have been considered phototoxic FQs; for example, enoxacin had an increased phototoxicity potential [37,288]. In an in vivo study which estimated the production of singlet oxygen and/or hydrogen peroxide, the order of the active oxygen species production was: lomefloxacin > ciprofloxacin > fleroxacin >> enoxacin > levofloxacin > ofloxacin > norfloxacin [289]. Additionally, several studies highlighted the phototoxic effects of enoxacin depending on the UVA irradiation dose in vitro [290]. Hayashi et al. [169] conducted a structure–phototoxicity study where they demonstrated that the substituent from the N1 position (e.g., 1-difluorophenyl 8-chloro) also influenced the phototoxic potentials of FQs, alongside the substituent from the C8 position.

#### 5.9.1. Underlying Mechanisms of Phototoxicity

FQs are often associated with the risk of phototoxicity, photoallergy, and photocarcinogenesis [291,292]. Phototoxicity is a dermatologic side-effect associated with FQs therapy, which appears consecutively to UVA and UVB radiation exposure. The intensity of phototoxicity varies from mild to severe and is observed a few hours after exposure. FQs are photosensitizer drugs and can produce a phototoxic skin reaction through several pathways [291,293,294,295]. Thus, FQs can undergo processes of gradual decomposition, formation of reactive oxygen species (ROS) (singlet oxygen and other free radicals), or return to the ground state, unchanged [292,293,296]. The appearance of ROS may occur by a type I mechanism (including hydroxyl radical (OH•), superoxide anion (O2^−^•), and hydrogen peroxide (H_2_O_2_)), a type II mechanism (including singlet oxygen (^1^O_2_)), and by a triplet energy transfer [292]. FQs can follow different photochemical processes, such as dehalogenation, decarboxylation, oxidation of the 7-amino substituent, production of superoxide anion, and generation of singlet oxygen [292]. The potential to produce singlet oxygen may vary depending on the chemical structures of the FQs [289]. The resulting products affect the lipid membranes (photoperoxidation) and other subcellular components (e.g., mitochondria), including lipids, proteins, and nucleic acids. Thus, an inflammatory process will occur. In addition, DNA is damaged under ROS action with varying degrees of severity [97,292,297].

A study by Marrot et al. [298] proposed a multistep mechanism. Inducing photooxidative stress is the first step of the mechanism. The second step of the mechanism is the production of some genotoxic effects by the formed photoproducts. On the other hand, some FQs are photosynthesizers through dehalogenation and the formation of aryl cations [299,300]. The 6,8-dehalogenated products of some FQs (e.g., lomefloxacin, fleroxacin, and sparfloxacin) were associated with photogenotoxic potential [292,300]. Lomefloxacin (a 6,8-difluoro derivative) proved to be the most phototoxic FQ [296,301]. As previously presented, these FQs have been withdrawn from the market due to AEs. Moxifloxacin proved to be the least phototoxic compared to other 8-substituted derivatives due to the 8-methoxy substituent [301,302]. Cuquerella et al. [300] found that photodegradation of ofloxacin under aerobic conditions undergoes N-demethylation as the primary process. Thus, the predominant photosensitized mechanism is type I (photoionization from the excited singlet state). Instead, oxidative DNA damage is a type II mechanism. Ofloxacin does not produce significant DNA oxidation, although H_2_O_2_ occurs in photodegradation.

#### 5.9.2. Reported Phototoxicity Associated with Modern FQs

Delafloxacin. In clinical trials, delafloxacin was not associated with phototoxicity AEs [51,191,193]. Oral delafloxacin, lomefloxacin, or placebo, once daily for 6 days in 52 healthy volunteers (male and female), were administered in 1 placebo/active-controlled, randomized phase 1 study. Delafloxacin showed no clinically relevant phototoxicity at dosages of 200 and 400 mg per day at any wavelength evaluated [303]. Furthermore, no participant in either group reported phototoxicity in a randomized, double-blind, global phase 3 trial comparing the efficacy and safety of delafloxacin 300 mg twice daily to moxifloxacin 400 mg once daily in adults with CABP [208]. Nonetheless, pooled safety events from pivotal phase 3 trials show that delafloxacin is well-tolerated in both i.v. and oral formulations. Delafloxacin is not associated with an increased risk of treatment-related phototoxicity [51,85].

Levonadifloxacin. Weak phototoxicity comparable with levofloxacin was found in the preclinical safety evaluation of the two forms of levonadifloxacin (*L*-arginine salt and *L*-alanine ester) [304]. In a phase 1 single and multiple ascending dose study (USA trial), no clinically significant abnormalities were observed in the phototoxicity assessments [305]. However, 4 subjects enrolled in an intrapulmonary pharmacokinetics study following oral administration of alalevonadifloxacin (1000 mg twice daily for five days) to 30 healthy Indian adults reported mild cases of photophobia [283]. Alalevonadifloxacin (WCK 2349) is the prodrug of levonadifloxacin (the name of alalevonadifloxacin emerged from *L*-alanine ester of levonadifloxacin at the hydroxyl substituent of piperidine moiety) [306].

Sitafloxacin. A few existing studies have reported conflicting results regarding the phototoxic potential of sitafloxacin. A randomized, controlled trial examined drug-induced phototoxicity related to using sitafloxacin, enoxacin, levofloxacin, and sparfloxacin in volunteers. In the Caucasian trial, sitafloxacin 100 mg twice daily caused mild ultraviolet UVA-dependent phototoxicity (median PI = 1.45) at 365 ± 30 nm (half-maximum bandwidth), which was maximal at 24 h and normalized 24 h after drug discontinuation. Interestingly, no clinically relevant phototoxicity was observed in either the sitafloxacin or placebo groups in the Oriental trial [307].

This is consistent with a prior psoralen photochemotherapy study, which found that more intensely pigmented (skin phototype IV) patients are marginally less susceptible to phototoxicity than fairer (skin types I and II) subjects [308]. In Thailand, a randomized controlled trial compared oral sitafloxacin to i.v. ceftriaxone, followed by oral cefdinir, in treating acute pyelonephritis and complicated UTI. There was no evidence of phototoxicity in any of the participants enrolled in this study [309].

For lascufloxacin, nemonoxacin, and zabofloxacin, no cases of phototoxicity associated with these FQs treatments were reported.

### 5.10. Clostridium difficile Infection

*Clostridium difficile* is a Gram-positive non-pathogenic bacterium that lives in the colon and can overgrow when the patient takes an antibiotic for another infection. This overgrowth may include symptoms such as diarrhea, fever, and pain. Additionally, *Clostridium difficile*-associated diarrhea may be mild to fatal [310]. Among the side-effects frequently reported for the administration of FQs, infection with *Clostridium difficile* is present. FQs were initially associated with a low risk compared with other antibiotics (e.g., clindamycin, cephalosporins) [166,311,312]. Later, several reports tried to establish a link between the administration of common FQs (ciprofloxacin, gatifloxacin, levofloxacin, and moxifloxacin) and the occurrence of infection with *Clostridium difficile* [312,313,314]. Dhalla et al. [311] found no increased risk of *Clostridium difficile*-associated disease requiring hospitalization concerning gatifloxacin or moxifloxacin compared to levofloxacin. Thus, the results were suggestive, and there were no firm conclusions, so more in-depth studies are necessary.

#### 5.10.1. Underlying Mechanisms of *Clostridium difficile* Infection

The therapy with broad-spectrum antibiotics or other drugs (gastric acid suppressants, NSAIDs drugs), age, some comorbidities (leukemia or lymphoma, diabetes, inflammatory bowel disease, renal failure, solid cancer), a poor immune response to the toxins of *Clostridium difficile*, dysbacteremia in the colon flora, and exposure to *Clostridium difficile* spores from the hospital (surfaces, roommates, or hand carriage by staff) are increasing factors of *Clostridium difficile* infection [315,316,317]. Additionally, the occurrence of *Clostridium difficile* infections associated with FQs is closely related to the emerging BI/NAP1/027 strains (resistant to respiratory FQs) [312]. In 2008, in Germany, ribotype 001 had a high prevalence, and ribotype 027 strains had restricted dissemination [318]. A pro argument is that the FQs restrictions were associated with the decrease in *Clostridium difficile* infections proportionally to the reduction of infections with FQs-resistant BI/NAP1/027 strains related to the more severe condition [319].

#### 5.10.2. Reported *Clostridium difficile* Infections Associated with Modern FQs

Delafloxacin. Peripheral neuropathy, hypersensitivity, and diarrhea caused by *Clostridium difficile* were listed by the FDA as possible dose-dependent side-effects. They were reported to be less severe than those caused by other FQs and only sporadically occurred. For instance, a pooled study comparing the incidence of AEs between delafloxacin and vancomycin/aztreonam across pivotal phase 3 trials involving patients with acute bacterial skin and skin structure infections (ABSSSI) found that only one patient in the two phases 3 trials considered (0.1%) developed a *Clostridium difficile* infection in the delafloxacin group. This treatment-emergent adverse event (TEAE) was classified as mild in severity and did not cause the discontinuation of the treatment. No cases were seen in the vancomycin/aztreonam (the comparator drugs) group [51,191,206].

A randomized clinical study in which delafloxacin was compared to the best available therapy in patients with surgical site infections also reported one case (0.75%) of *Clostridium difficile* colitis [236]. In addition, two subjects (0.5%) in the delafloxacin group and one subject (0.2%) in the moxifloxacin group had a TEAE of *Clostridium difficile* colitis in a randomized, double-blind, global phase 3 study involving adult subjects with CABP. As a result of the AE, one subject in each treatment group discontinued treatment [208]. Although most studies reported diarrhea as the most common gastrointestinal disorder associated with delafloxacin treatment, no connections were made between this side-effect and potential *Clostridium difficile* infections.

Lascufloxacin. The most common AE associated with lascufloxacin (Lasvic^®^ Tablets 75 mg) is diarrhea [320], which has been associated with other antibacterial agents, including FQs with *Clostridium difficile* infections. However, no direct link was made between diarrhea and *Clostridium difficile* infections in clinical trials involving lascufloxacin [161,237].

Levonadifloxacin is generally well-tolerated. One patient on i.v. therapy was reported with diarrhea in a retrospective, multi-center study involving 227 patients. The episode was mild, subsequently resolved, and was not directly associated with a potential *Clostridium difficile* infection. There were no serious AEs reported in the patient records, nor was levonadifloxacin therapy discontinued due to AEs [321].

Nemonoxacin. While mild cases of diarrhea have been reported following single and multiple oral doses of nemonoxacin, *Clostridium difficile* infections were not confirmed in any of these studies [238,239,285].

Sitafloxacin. *Clostridium difficile* colitis was reported by 1/48 patients (2.08%) receiving 100 mg of sitafloxacin once daily in a nested cohort within a multicenter clinical trial in patients with CAP caused by *Streptococcus pneumoniae*. The AEs were mild in severity and did not cause treatment discontinuation [241].

Zabofloxacin. Both salts, zabofloxacin hydrochloride and zabofloxacin aspartate, were well-tolerated in clinical trials in healthy patients [243] and patients with COPD with moderate exacerbations [21,242]. In addition, there were no reports of *Clostridium difficile* infections in these studies, albeit diarrhea, a common symptom, was listed as an adverse drug reaction.

### 5.11. QT Prolongation (Fatal Arrhythmia) and Torsade de Pointes

One of the most common AEs associated with antibiotics therapy is QT prolongation with ventricular arrhythmia. In some patients (with coronary artery disease and electrolyte disturbances), torsade de pointes and sudden death can occur [227]. The time interval from the start of the Q-wave to the end of the T-wave is known as the QT interval. In the QT interval, the ventricular depolarization and repolarization intervals are included. QT prolongation is mainly based on the increased repolarization duration through the blockade of the cardiac K^+^ channel. QT prolongation is a severe AE associated with some FQs therapies (gatifloxacin, grepafloxacin, levofloxacin, moxifloxacin, sparfloxacin) [322,323]. Thus, the proarrhythmic potential varies from one QN to another [114,322,324]. QT prolongation can be associated with FQs therapy and is classified as an inherited long QT syndrome or an acquired long QT syndrome [325,326]. In addition, QT prolongation may lead to the occurrence of arrhythmia [327]. Torsade de pointes is a potentially fatal polymorphic ventricular tachyarrhythmia characterized by the twisting of points on an electrocardiogram (ECG), often preceded by a prolonged QT interval [325,326,328,329].

Among FQ-induced QT interval prolongation were levofloxacin, moxifloxacin, gatifloxacin, gemifloxacin, grepafloxacin, and sparfloxacin [330]. Grepafloxacin (1999) and sparfloxacin (2001) were withdrawn from the market due to severe cardiotoxicity, including QT prolongation (Table 3). Previously, both FQs were assessed as the most potent inhibitors of hERG and associated with pro-arrhythmia (including fatal arrhythmias) [330].

Ciprofloxacin may be associated with QT prolongation; rarely, it can be associated with torsade de pointes. These severe AEs could occur in patients with predisposing factors [331,332]. In a clinical trial conducted in healthy adult volunteers, the effect of a single dose of ciprofloxacin, levofloxacin, and moxifloxacin on the QT and rate-corrected QT (QTc) was evaluated. QT and QTc intervals compared with placebo were higher after the moxifloxacin dose than levofloxacin and ciprofloxacin [333]. Other studies confirm these findings concerning those three FQs [334]. Stancampiano et al. [335] observed 1004 hospitalized patients with prolonged QTc and concluded that levofloxacin might be a safe option in patients with prolonged QTc. Moxifloxacin is associated with QTc interval prolongation starting at 11.5 to 19.5 ms. A higher incidence of QTc interval prolongation was reported in patients with pneumonia and when moxifloxacin is i.v. administered compared to oral formulations. The moxifloxacin-induced QT interval prolongation could lead to torsade de pointes [329,336]. Additionally, data obtain on moxifloxacin can estimate the proarrhythmic potential of new drugs in development [337].

In addition, there are sex differences in the cardiac safety assessment of FQs. A recent comparative study showed that women are more affected than men, and moxifloxacin was a more potent prolonger of the QT interval versus levofloxacin [338]. Moxifloxacin sensitivity to a prolonged QT interval was independent of ethnicity in a study that included Japanese and Caucasian subjects [339].

#### 5.11.1. Underlying Mechanisms of QT Prolongation (Fatal Arrhythmia) and Torsade de Pointes

The mechanism of FQ-induced QT interval prolongation is based on the blockade of the human cardiac K+ channel (*human ether-a-go-go related gene,* hERG). Additionally, hERG channel blockade is an essential predictor of proarrhythmic activity in screening new drugs [322]. The inhibition of hERG is not an FQs class effect due to the variability from one compound to another. Thus, the inhibition of hERG is highly dependent upon specific substituents of the FQs and other unidentified factors [114,324]. It should be noted that the antibacterial FQs do not inhibit isoenzymes CYP3A4, CYP 2C9, and CYP 2C19 and follow linear pharmacokinetics as other cardiotoxic drugs [325]. The differences in FQ-induced QT interval prolongation in men and women are given by the sex hormones that influence cardiac ion channel expression [338].

#### 5.11.2. QT Prolongation (Fatal Arrhythmia) and Torsade de Pointes Associated with Modern FQs

Delafloxacin. In clinical trials, only syncope and loss of consciousness were reported in the standardized MedDRA queries for potential QT prolongation. Delafoxacin therapy was not associated with QT prolongation and torsade de pointes events [51,185,191,204]. A randomized, double-blind, 4-period crossover study conducted in healthy adults assessed the effect of delafloxacin on the corrected QT (QTc) interval after dosing with delafloxacin at 300 mg i.v. (therapeutic dose) and at 900 mg i.v. (supratherapeutic dose). Neither of the two delafloxacin dosing groups had an upper bound that approached or exceeded 10 ms, indicating no clinically significant increase in the QTcF (QT interval adjusted for heart rate using the Fridericia method) interval [340]. A phase 3, randomized, double-blind, global study compared the efficacy and safety of delafloxacin 300 mg twice daily to moxifloxacin 400 mg once daily in adults with CABP and found that no subjects in the delafloxacin group versus two subjects (0.5%) in the moxifloxacin group experienced QT prolongation [208].

Lascufloxacin. The drug information sheet of Lasvic^®^ Tablets 75 mg (lascufloxacin hydrochloride) developed by Kyorin Pharmaceutical (Japan) mentions the QT interval prolongation and ventricular tachycardia as rare AEs (includes torsade de pointes) [320]. However, to the extent of our knowledge, clinical studies did not report any events associated with these AEs.

Levonadifloxacin. No clinically significant abnormalities were reported in the electrocardiographic evaluations in other phase 1 and 3 clinical trials. In 48 healthy subjects, the electrocardiographic (ECG) effects of alalevonadifloxacin at a supratherapeutic oral dose of 2600 mg were compared to a placebo and oral moxifloxacin (400 mg). Alalevonadifloxacin was found not to affect the baseline and placebo-corrected QTcF, QRS, or PR interval [341]. Furthermore, clinically indicated doses of 1000 mg are not anticipated to impact cardiac conduction or repolarization, except for a potential temporary increase in heart rate by a maximum of 14.4 beats per minute, which seems clinically negligible [321,341]. Therefore, levonadifloxacin constitutes a viable substitute for antibiotics that prolong the QT interval, including macrolides and other FQs.

Nemonoxacin. The existing studies concerning QT prolongation as AE suggest the administration of nemonoxacin with caution, especially in high doses requiring careful monitoring. The associated risk of prolonging the QT interval seems similar to levofloxacin. Nemonoxacin was found to be well-tolerated up to the maximum dose of 1500 mg in a double-blind, ascending single-dose study including 56 healthy participants. No severe AEs were reported, and electrocardiograms revealed no clinically significant abnormalities [284]. In a randomized, double-blind, multicenter study, no cases of ECG QTc interval prolongation were observed in the 500 mg group. In contrast, nemonoxacin 750 mg caused prolongation of the QTc interval in 2.3% of the patients. In comparison, 3.3% of the patients in the comparator group receiving 500 mg of levofloxacin experienced QTc interval prolongation [239].

Another clinical trial investigating the effects of nemonoxacin treatment in healthy Chinese volunteers following single and multiple oral doses reported no QTc difference from the baseline compared with the placebo for the single-dose safety study and no increase in QTc values with dose [285]. Two single-dose, open-label, randomized, crossover investigations involving 24 healthy male Chinese volunteers revealed similar findings. There were no clinically significant alterations in the vital signs, and the ECG was normal in all patients enrolled in the study [342]. No QTc difference from the baseline compared with the placebo or increase in QTc values with dose were also reported by a randomized, double-blind, dose-escalating safety and tolerability study in 92 healthy Chinese subjects [148].

Twelve healthy Chinese volunteers participated in an open-label, randomized crossover trial in which one incident of sinus bradycardia and one event of a prolonged QT on the ECG were thought to be possibly connected to the study drug. All AEs were minor, resolved without treatment, and did not cause treatment discontinuation [343]. However, drug-related QT interval prolongation occurred in all three treatment groups in a randomized, double-blind, phase 2 study comparing the efficacy and safety of oral nemonoxacin with oral levofloxacin in the treatment of CAP [240].

The effects of oral nemonoxacin 500 mg (therapeutic dose), nemonoxacin 750 mg (supratherapeutic dose), moxifloxacin 400 mg (positive control), or placebo on the QT/QTc interval were investigated in a randomized, placebo- and positive-controlled trial in 48 healthy Chinese adults. Nemonoxacin 500 mg caused a QTc interval prolongation of 5–10 ms, which was considered unlikely to be dangerous. However, the QTc interval prolongation after treatment with nemonoxacin 750 mg (>10 ms, ≤15 ms) was classified as potentially hazardous. These findings suggest that the therapeutic dose of nemonoxacin (500 mg) has a tolerable and acceptable effect on cardiac repolarization, whereas the administration of the supratherapeutic dose (750 mg) necessitates careful monitoring. Notably, the QTcF values after eating high-fat food did not significantly differ from those in the fasted condition [344].

A prolonged QT interval was also reported in 0.8%, 2.6%, and 1.5% of participants in the nemonoxacin 500 mg, nemonoxacin 750 mg, and levofloxacin groups, respectively, in three multicenter, randomized, double-blind, parallel, comparative clinical trials conducted in Taiwan, South Africa, and China. The results suggested that nemonoxacin may have a comparable risk of prolonging the QT interval to levofloxacin [30]. Furthermore, in a population pharmacokinetics study of nemonoxacin conducted in six Chinese patients with moderate hepatic impairment, a mild T-wave alteration was reported in 1 subject and a prolonged QT interval in 2 subjects. Meanwhile, the healthy volunteers reported ventricular premature beat (1 subject), T-wave changes (2 subjects), and sinus bradycardia (1 subject). According to these findings, it is advised that patients with severe hepatic impairment take nemonoxacin under ECG monitoring [345].

Sitafloxacin. No cases of cardiotoxic adverse events associated with sitafloxacin treatment were reported.

Zabofloxacin. No cases of cardiotoxic adverse events associated with zabofloxacin treatment were reported. For instance, a randomized, open-label, single-dose, 2-way crossover trial conducted in 32 healthy Korean male volunteers found that the QT interval did not suffer significant changes following zabofloxacin administration compared with baseline [242].

### 5.12. Dysglycemia/Hypoglycemia and Hyperglycemia

FQs therapy is linked with alterations in glucose metabolism, such as an increased risk of dysglycemia or severe hypoglycemia [346,347,348]. Many studies have highlighted this adverse reaction. For example, the hypoglycemia associated with levofloxacin can be persistent, severe, and life-threatening. However, this effect is reversible upon discontinuation of treatment [346,349]. Gatifloxacin (from third generation) was withdrawn from the market (2008) due to severe or even fatal hypoglycemia [116,350]. Additionally, gatifloxacin was associated with hyperglycemia [351,352]. In a systematic review conducted by Murad et al. [353] for drugs reported to cause hypoglycemia, 32 publications using QNs were analyzed (including 826 patients). Gatifloxacin was highlighted (moderate quality of evidence) from the group of targeted FQs (ciprofloxacin, clinafloxacin, levofloxacin, moxifloxacin, and sparfloxacin) (low-quality of proof).

As previously presented in Table 2, in 2018, the FDA reinforced safety information about severe low blood sugar levels associated with FQs, requiring safety labeling changes for FQs class [68]. In a systematic review conducted to evaluate the safety of FQs in diabetic patients, Althaqafi et al. [235] found that the administration of moxifloxacin was the most associated with dysglycemia. Comparatively, ciprofloxacin was the least associated with dysglycemia.

#### 5.12.1. Underlying Mechanisms of Dysglycemia

The exact mechanisms by which FQs disturb sugar levels in the blood are unknown. However, there are some hypotheses concerning hypoglycemia side-effects: (a) sulfonylurea-like action: FQs may bind to the ATP-sensitive K^+^ channels (block the channels), such as secretagogues responsible for the insulin release from the pancreas, and the depolarized beta-cell membrane will allow the entry of calcium and the release of insulin, and (b) FQs may inhibit the activity of P450 isoenzymes (CYP2C9, CYP2C8, CYP3A4), which are responsible for several antidiabetics drugs’ metabolism; consequently, the serum level of antidiabetics will be increased, and the blood sugar level will be much decreased [347,348]. In a case report, gatifloxacin induced hyperglycemia in an elderly patient with no diabetes, and the mechanism may be related to the vacuolation of pancreatic beta-cells, which leads to decreased insulin levels [351]. Increased epinephrine secretion was demonstrated in an experimental animal model (diabetic and normal rats) following gatifloxacin administration [354].

#### 5.12.2. Reported Dysglycemia and Hypoglycemia Associated with Modern FQs

Delafloxacin. In a randomized, double-blind, phase 2 trial, a total of 150 patients were administered i.v. delafloxacin or tigecycline. Here, 11 out of 100 delafloxacin-treated patients and 1 tigecycline-treated patient had lower-than-normal glucose levels after having had normal values at baseline. However, only one patient reported an adverse effect of hypoglycemia, and the remaining patients were asymptomatic [206]. In another double-blind, phase 2 trial, 256 patients received either 300 mg of delafloxacin, 15 mg/kg of vancomycin (actual body weight), or 600 mg of linezolid, each administered i.v. twice daily, for 5–14 days. TEAEs of hyperglycemia occurred in two, two, and one patients in the delafloxacin, vancomycin, and linezolid groups, respectively. All hyperglycemia TEAEs were rated as mild. However, the investigational drugs were delivered in 5% dextrose, which may have impacted hyperglycemia reports. No patient reported TEAEs related to hypoglycemia in this study [355].

However, in other clinical studies involving delafloxacin, hypoglycemic and hyperglycemic events were reported as TEAEs as uncommon complications. In a phase 3, randomized, double-blind study comparing the efficacy and safety of delafloxacin to vancomycin/aztreonam in patients with ABSSSI, one delafloxacin-treated patient and two vancomycin/aztreonam-treated patients reported hypoglycemia related to treatment, and two delafloxacin patients and one vancomycin/aztreonam patient reported hyperglycemia potentially related to treatment. The reported symptoms were mild or moderate in severity [51,191,207]. Pharmacokinetic studies noted no relevant differences in blood glucose levels in 12 h following delafloxacin doses [51,191,206].

Lascufloxacin. The drug information sheet of the product Lasvic^®^ Tablets 75 mg mentions hypoglycemia as a rare adverse reaction [320]. However, the few clinical studies available for lascufloxacin did not report any hypoglycemia-related TEAEs [237,356,357].

Levonadifloxacin. In a phase 3, a randomized, active-comparator study involving 500 subjects with ABSSSI, 1.6% displayed above-normal blood glucose values or hyperglycemia. In this study, oral levonadifloxacin 1000 mg was compared with oral linezolid 600 mg, and i.v. levonadifloxacin 800 mg was evaluated in comparison to i.v. linezolid 600 mg, where each drug was administered twice daily for 7–10 days. However, these adverse events were of mild to moderate severity and not related to levonadifloxacin, and most patients had high blood glucose at screening. Noteworthy, levonadifloxacin displayed favorable clinical and microbiological efficacy in diabetic patients, including diabetic foot infections caused by Gram-positive pathogens such as MRSA [41].

For nemonoxacin, sitafloxacin, and zabofloxacin, no cases of dysglycemia were reported associated with these drugs.

### 5.13. Hepatotoxicity

Although many side-effects are associated with FQs included in the EMA and FDA warnings, the hepatotoxicity of these drugs is not in the foreground [35,66]. The usual FQs are rarely associated with severe liver damage with increased transaminase levels (estimation of 1:100,000 patients who received treatment with FQs). However, some FQs were withdrawn as a result of serious side-effects, including hepatotoxicity (e.g., temafloxacin, trovafloxacin, and gatifloxacin (Table 3)) [287,358,359,360,361].

Ciprofloxacin is one of the most frequent FQs associated with hepatotoxicity, while levofloxacin and moxifloxacin were less associated [358,359,360,361,362]. In a prospective study based on data from the Drug-Induced Liver Injury Network (DILIN) of the National Institute of Diabetes and Digestive and Kidney Diseases (USA), only 12 patients developed liver injury out of a total of 679. In this group, six were treated with ciprofloxacin, four with moxifloxacin, one with levofloxacin, and one with gatifloxacin [363]. Another retrospective case-control study indicated that ciprofloxacin was associated with an increased risk of hepatotoxicity compared to levofloxacin and moxifloxacin. The number of patients included in the research and exposed to FQs was 7862 [364]. Additionally, recently evaluated data from the FDA Adverse Event Reporting System pointed out ciprofloxacin association with acute hepatic failure, having a marginal and a significant signal compared to the other three FQs (levofloxacin, moxifloxacin, and ofloxacin) with a weak and non-significant signal [365].

#### 5.13.1. Underlying Mechanisms of Hepatotoxicity

Rarely, FQs may cause idiosyncratic liver injury as a class effect. The liver injury appears starting from the first week to four weeks of treatment [358,363]. Liver damage includes hepatocellular necroses (profound alterations), cholestasis, and immune allergic reactions [360,363]. Among the FQs, trovafloxacin was associated with liver failure due to hepatic necrosis with rapid onset (even after two days of treatment) [112,361]. One hypothesis of the mechanism by which trovafloxacin produces hepatotoxicity is closely related to the transformation of cyclopropylamine moiety under cytochrome P450 enzymes and myeloperoxidase. Additionally, trovafloxacin contains other oxidizable moieties (e.g., a difluoroaniline system) and may produce reactive metabolites responsible for liver injuries [366].

The naphthyridone structure of some FQs was not directly associated with hepatotoxicity. Thus, two or three nitrogen atoms in the chemical structure of FQs could be related to liver injury. Some naphthyridone FQs are enoxacin, tosufloxacin, trovafloxacin, and gemifloxacin. Of these, only trovafloxacin was withdrawn due to severe hepatotoxicity (Table 3) [37]. Other FQs, such as ciprofloxacin, levofloxacin, and moxifloxacin, were associated with hepatocellular and cholestatic hepatitis [361]. A case report points out that ciprofloxacin is associated with increased hepatic transaminases. This adverse reaction was successfully resolved by replacing ciprofloxacin with levofloxacin [362]. However, FQs slightly increase alanine transaminase (ALT) levels in serum and liver injury rarely occurs, even when moxifloxacin and levofloxacin are administered to treat tuberculosis as alternative drugs [361].

#### 5.13.2. Reported Hepatotoxicity Associated with Modern FQs

Delafloxacin has been associated with increased liver enzyme levels [204]. Hoover et al. [367], in multiple ascending dose studies regarding delafloxacin, noticed that some participants had increased ALT levels. Thus, the association of these ALT elevations with delafloxacin was not clearly established. In a double-blind, phase 2 trial, 256 patients were randomized (1:1:1) to receive 300 mg of delafloxacin, 600 mg of linezolid, or 15 mg/kg of vancomycin (actual body weight), each administered i.v. twice daily, for 5–14 days. A total of five patients (two on delafloxacin and three on vancomycin) had six TEAEs related to liver toxicity. None of these events resulted in treatment discontinuation or serious AEs. Three subjects displayed TEAEs of “liver function test abnormal”: two in the delafloxacin group (one considered possibly related to the investigational drug and one probably related) and one in the vancomycin group (considered possibly related to the study drug). Notably, one of these subjects (in the delafloxacin group) had high ALT and aspartate aminotransferase (AST) values at screening. The subject experienced additional increases at follow-up and late follow-up [355].

Across two phase 3 studies (for ABSSSIs), the incidence of hepatic events was similar between delafloxacin and comparators (vancomycin/aztreonam). Generally, the hepatic side-effects of delafloxacin therapy were mild or moderate in severity. Additionally, Hy’s Law criteria (a method to predict if a drug may induce severe liver injury based on transaminases and total bilirubin level) were not violated for any patient in the study [51,368]. In addition, no patient discontinued the treatment with delafloxacin due to hepatic side-effects [51]. Additionally, in the phase 3 study, both formulations of delafloxacin (i.v. and oral) were compared to vancomycin/aztreonam. There were no increases in hepatic events in the delafloxacin groups versus comparators. Elevated levels of AST had four patients in the delafloxacin group versus two in the vancomycin/aztreonam group. However, Hy’s law criteria were not violated for any patient in the study [206].

A randomized, double-blind, global phase 3 study compared the efficacy and safety of delafloxacin 300 mg twice daily versus moxifloxacin 400 mg once daily in adults with CABP. Here, 5.1% of the subjects in the delafloxacin group, compared to 2.8% in the moxifloxacin group, reported hepatic TEAEs, with increased transaminases being the most frequent AEs. Except for one subject in the moxifloxacin group, the hepatic TEAEs were all mild or moderate in severity. Due to elevated liver enzymes, there were two and one treatment discontinuations for delafloxacin and moxifloxacin, respectively. Similar percentages of subjects had ALT or AST values five times higher than the upper limit of normal in the delafloxacin (1.4% and 0.9%) and moxifloxacin (1.6% and 0.5%) groups [208].

Lascufloxacin. A phase 3 double-blind comparative study of lascufloxacin versus levofloxacin in patients with CAP reported a drug-related ALT increase in 1 out of 140 patients. The results were similar between patients in the lascufloxacin and comparator (levofloxacin) groups. Moreover, a drug-related eosinophil count increase was observed in 3 out of 140 patients versus 2 in 137 patients in the comparator group [237].

Levonadifloxacin. Oral levonadifloxacin was well-tolerated in subjects with normal and impaired hepatic functions with no dose adjustment [341].

Nemonoxacin. In a study assessing the safety and clinical pharmacokinetics of nemonoxacin, healthy Chinese volunteers received either a placebo or single oral doses of nemonoxacin of 125, 250, 500, 750, or 1000 mg. In the multiple-dose pharmacokinetic study, one subject in the 500 and 750 mg groups had mild to moderate transaminase elevations [285]. Compared to an earlier nemonoxacin phase I study in the USA [238,284], the Chinese volunteers in this study had fewer clinical but slightly more laboratory AEs, such as transaminase elevations [285].

In the analysis of two single-dose, open-label, randomized crossover studies conducted in 24 healthy male Chinese volunteers (12 per study), only 1 episode (4.16%) of increased ALT following administration of nemonoxacin was considered possibly drug-related [342]. Similarly, in a study which evaluated the safety and pharmacokinetic/pharmacodynamic profiles of nemonoxacin in healthy Chinese volunteers, the most common AEs were increased ALT, AST, and total bilirubin levels. The AEs described above spontaneously resolved during infusion or within 24 h of administration [286]. A randomized, double-blind, phase 2 study comparing the efficacy and safety of oral nemonoxacin versus oral levofloxacin in the treatment of CAP reported 25 episodes of laboratory abnormalities (primary leukopenia and elevated ALT level) in 16 subjects (25.8%) [240]. Another study involving healthy Chinese adults reported an ALT increase in 4.2% of the subjects treated with nemonoxacin 500 mg versus 2.1% of the subjects treated with nemonoxacin 750 mg, and 2.1% for the group treated with the comparator drug moxifloxacin. AST elevation was only reported in the group treated with nemonoxacin 750 mg (2.1%) [344].

It is unclear why these differences occur. In a pharmacokinetics study of six Chinese patients with moderate hepatic impairment, there was an increase in total bilirubin in two subjects, an increase in ALT in one subject, and an increase in AST in one subject. Except for one AE in a subject with an increased total bilirubin level until the last follow-up visit, all the above events were resolved without treatment. However, the elevated total bilirubin level was considered due to the preexisting condition rather than the investigated drug [345]. ALT elevation (nemonoxacin 5.1%, levofloxacin 4.1%), white blood cell decrease (nemonoxacin 2.0%, levofloxacin 1.2%), nausea (nemonoxacin 3.1%, levofloxacin 1.8%), and vomiting (nemonoxacin 1.7%, levofloxacin 2.3%) were common drug-related AEs in a phase 3, multicenter, randomized, double-blind trial. All the adverse events were of moderate severity [369]. Three randomized, double-blind clinical trials conducted in Taiwan, South Africa, and China reported elevated levels of hepatic enzymes (ALT, AST, and γ-glutamyl transferase) as the most frequently reported drug-related AEs in the nemonoxacin group. Patients in these studies received either nemonoxacin 500 mg or 750 mg or levofloxacin 500 mg. The nemonoxacin 750 mg group had an overall higher incidence of abnormal liver function. Notably, the incidence of increased ALT was higher for the nemonoxacin 500 mg than the nemonoxacin 750 mg and the levofloxacin 500 mg groups. Due to increased blood bilirubin and conjugated bilirubin levels, one event resulted in treatment discontinuation in the nemonoxacin 500 mg group [30].

Sitafloxacin. There are few studies regarding signs of hepatotoxicity associated with sitafloxacin. For example, increased levels of ALT (9.2% in the 100 mg once-daily group and 5.4% in the 50 mg twice-daily group), AST (8.2% in the 100 mg once-daily group and 9.6% in the 50 mg twice-daily group), and increased eosinophil counts (3.1% in the 100 mg once-daily group and 7.2% in the 50 mg twice-daily group) were also listed as frequent adverse drug reactions in a pharmacokinetic–pharmacodynamic analysis of two clinical trial results for community-acquired respiratory tract infections [370]. In a nested cohort within a multicenter clinical trial evaluating the safety and efficacy of sitafloxacin against CAP caused by *Streptococcus pneumoniae*, 14.6% of the patients in the 100 mg once-daily group and 8.33% in the 50 mg twice-daily group reported increased ALT, and the exact percentages in both groups reported increased AST [241]. Nevertheless, sitafloxacin was generally well-tolerated, and no serious adverse events occurred in either group.

For zabofloxacin, no cases of hepatotoxic AEs were reported.

### 5.14. Genotoxicity

Currently, the genotoxic potential of FQs is insufficiently studied. Genotoxicity of FQs can lead to AEs when they are administered to children, adolescents, and pregnant or lactating women. Additionally, the genotoxic potential of FQs that reach the environment is worrying. In the following, pieces of evidence of the genotoxic potential of FQs resulting in several relevant studies are presented.

In 1998, Hartmann et al. [371] proved that ciprofloxacin is responsible for most of the genotoxic effects detected in hospital wastewater samples by the *umuC* test. Ciprofloxacin was determined in native hospital wastewater by reversed-phase high-performance liquid chromatography (RP-HPLC) and fluorescence detection. Ciprofloxacin concentrations were found to be between 3 and 87 μg/L. A log-linear correlation (r2 = 0.84, *p* < 0.0001) was found between the *umuC* induction factor and ciprofloxacin concentrations in wastewater samples from 16 hospitals in Switzerland [371]. Additionally, the genotoxic potential of ciprofloxacin was studied by İkbal et al. [372] in cultured human lymphocytes in patients with a UTI. The results suggested that ciprofloxacin may have a moderate genotoxic potential due to the cytotoxicity and mild genotoxic effects in human peripheral blood lymphocytes [372]. A quantum chemical modeling method was applied to find the relationships between the quantitative structure indices and genotoxic potentials of 18 QNs. Thereby, a quantitative structure–activity relationship (QSAR) model for predicting the genotoxicity of QNs was established. The minimal inhibitory concentration (MIC_50_) values against *Streptococcus pneumoniae* were dependent on the genotoxic potentials (except for the FQs with pyrrolidine or cyclopropyl moieties at the C7 position) [373].

Nalidixic acid, ciprofloxacin, and enrofloxacin were evaluated from the genotoxic point of view alone and in mixtures using a sensitive micronucleus test on *Vicia faba* roots. LUFA standard soil was contaminated by FQs at four different concentrations and was in direct contact with *Vicia faba* roots. The *Vicia faba* micronucleus test enables the detection of genetic alterations. Significant micronuclei induction had been detected at the highest concentrations of the three FQs, and no significant genotoxic effect was detected for the lowest concentrations. In addition, the mixture of the three FQs at a very low concentration, similar to concentrations found in contaminated soils, presented genotoxic effects, most likely due to the synergy between the compounds [374]. Additionally, some impurities in FQs are suspected of having genotoxic potential. For example, descarboxyl levofloxacin is known as an isolated impurity of levofloxacin. In silico and in vitro methods were used to assess the genotoxicity of this impurity. Descarboxyl levofloxacin triggered a mutagenic structural alert. However, the results excluded the genotoxic potential of this impurity, considering its low concentration and the levofloxacin genotoxicity potential [375].

In one study, the main parameters affecting the genotoxicity of FQs were screened through two-dimensional quantitative structure–activity relationship (2D-QSAR) and principal component analysis (PCA) techniques. Additionally, these methods were combined with other sensitivity analysis methods to screen the rules affecting the genetic toxicity of FQs. Thus, it turned out that the chemical structure and the critical temperature were the main factors influencing FQs’ genotoxicity. It tested seven parameters affecting the FQs’ genotoxicity. The genotoxicity decreased with increasing the steric parameter, quadrupole moments QXX and QYY, and the boiling point. Thus, the genotoxicity increased with increasing the total energy, critical temperature, and molecular weight. These results could be helpful in the future molecular design of new antibacterial FQs with less genetic toxicity [376]. On the other hand, the HQSAR method and molecular docking could be helpful in the design of FQs derivatives with high genotoxicity towards Gram-negative bacteria. Thereby, in a study based on these two in silico methods, amifloxacin derivatives with highly hydrophilic groups at the C7 position presented high genotoxicity, increased stability of the FQs-topoisomerase IV-DNA complex, and increased antibacterial activities [377,378].

#### 5.14.1. Underlying Mechanism of Genotoxicity

The genotoxic potential of FQs is closely related to the chemical structure of the compounds (Table 5). The C6-fluorine atom increases the genotoxic potential of the compounds, although it is valuable for improving the antibacterial activity [8,11]. Currently, in the design of new compounds there is a tendency to remove the fluorine atom from the C6 position, with an example of non-fluorinated FQ being nemonoxacin [8,379]. The removal of the C6-fluorine substituent can be compensated with other responsible substituents for increasing the antibacterial activity and broadening the activity spectrum. The C8-methoxy substituent of nemonoxacin improves its antibacterial efficacy against Gram-positive bacteria due to its action on the two target enzymes (DNA gyrase and topoisomerase IV) [379,380].

The genotoxic potential is present in the FQs with an amino or methyl substituent at the C5 position [11,167,168]. Additionally, some substituents at the C7 position, such as pyrrolidinyl, piperazine, and alkyl, confer genotoxicity to the FQ compounds in the following order: pyrrolidine > piperazine > alkyl [11,167]. Thus, the substitution of these groups decreases the genotoxic potential, such as: pyrrolidine (unsubstituted) > piperazine (unsubstituted) > pyrrolidine (substituted) > piperazine (substituted) [168]. Substituted piperidine [(3*S*,5*S*)-3-amino-5-methylpiperidin-1-yl] was preferred in the structure of nemonoxacin at the C7 position [380]. Another study highlighted increased genotoxicity of the QNs after the chlorination process of the waters if at N1 position they contain hydrophilic substituents with fewer H-bond donors or negative charge. Comparable levels of genotoxicity were found in ultrapure water and secondary effluent matrices [381].

Genotoxic effects of some FQs were recently established by Bhattacharya et al. [382] through several combined methods (UV-vis absorption titration, fluorescence-based competitive ethidium bromide displacement assay, and molecular docking). Five of the FQs most used in therapy were studied: ciprofloxacin, levofloxacin, moxifloxacin, norfloxacin, and ofloxacin. The hypothesis of their study was the ability of FQs to intercalate healthy human DNA and to produce oxidative DNA damage. At an FQs concentration above 50 mg/mL in the MTT (3-(4,5-dimethylthiazol-2-yl)-5-(3carboxymethoxyphenyl)-2-(4-sulfophenyl)-2H-tetrazolium) assay, a significant decrease in cell viability was obtained for ciprofloxacin, moxifloxacin, and norfloxacin. Additionally, FQs produced DNA oxidative damage according to Western immunoblotting studies’ results. This study showed DNA intercalation with binding constants of the order 10^4^. In conclusion, the selected FQs presented moderate to good DNA-damaging properties [382]. In addition, QSAR and 3D Pharmacophore models were used to screen essential positions and substituents contributing to the genotoxicity of 21 FQs. Among the four positions finally examined (N1, C5, C7, C8) through full factorial design, the C5 and C7 positions have a dominant effect, being the main effect on FQ genotoxicity. The N1 and C8 positions have a second-order interaction effect. A more substantial second-order interaction effect and lower genotoxicity characterized two adjacent positions. The obtained results regarding the structural modification were validated on new FQs derivatives. These modified derivatives presented lower genotoxicity and higher efficacy, with their synthesis being feasible [383].

#### 5.14.2. Genotoxicity Associated with Modern FQs

Delafloxacin. No genotoxicity effects (neither mutagenic nor clastogenic) were identified in in vitro studies. Additionally, in in vivo studies, genotoxicity was negative at the highest possible dose [152,154].

Lascufloxacin. Genotoxicity of lascufloxacin was assessed as similar or decreased versus other QNs in the toxicity studies in animals [384].

Levonadifloxacin. No genotoxicity was found in the preclinical safety evaluation of levonadifloxacin as *L*-arginine salt and *L*-alanine ester formulations [304].

Nemonoxacin. Although in vitro studies presented positive results regarding genotoxicity, in vivo tests presented negative results [385].

Sitafloxacin. The predicted genotoxic potential (EC_10_) in a QSAR model was higher (442.7 nM) than the other 13 QNs (out of a total of 15 compounds) [373]. To the extent of our knowledge, there are insufficient published data regarding the genotoxic potential of sitafloxacin.

Zabofloxacin. To the extent of our knowledge, there are insufficient published data regarding the genotoxic potential of zabofloxacin.

### 5.15. Severe Hemolytic-Uremic Syndrome

Hemolytic anemia is a less frequent side-effect of FQs in patients with a deficiency of the enzyme glucose-6-phosphate dehydrogenase [5]. Among the FQs used in therapy, temafloxacin was withdrawn from the market in 1992 due to its association with severe hemolysis with or without other organ system dysfunction, described as a “temafloxacin syndrome” [37,49,92,97]. An estimated number of 189,000 prescriptions were reported to the FDA over 100 cases of “temafloxacin syndrome” [5]. After about six days of treatment, the signs of “temafloxacin syndrome” were fever, chills, jaundice, vomiting, abdominal pain, myalgia, and back pain. Hemolysis based on the drop in hemoglobin level, renal dysfunction, which required dialysis for 34 cases, and coagulopathy (as a hepatic dysfunction) were recorded. In some patients, CNS side-effects were observed. Two patients died. Ten patients developed this syndrome after the first administered dose [5,49]. In subsequent years, concerns about hemolytic-uremic syndrome remained for all approved FQs.

In a Japanese study conducted by Shiomi et al. [386], fifteen patients during the Sakai outbreak of *Escherichia coli* O157:H7 infection did not develop hemolytic-uremic syndrome associated with FQs (norfloxacin and sparfloxacin). In vitro, FQs were reported to be associated with the production induction or release of Shiga-like toxins from *E. coli* O157:H7. However, this study concluded that an oral FQs regimen for five days effectively prevented the development of the hemolytic-uremic syndrome and treated *Escherichia coli* O157 infection in all patients. Two meta-analysis focuses on the antibiotic therapy of *Escherichia coli* O157:H7 infection and the risk of the hemolytic-uremic syndrome. The results did not show a higher risk of hemolytic-uremic syndrome associated with antibiotic therapy, including FQs [387,388]. Ohnishi et al. [389] observed fifteen patients infected with verotoxin-producing *Escherichia coli* O157 (VTEC O157), including six patients treated with levofloxacin and nine patients without an antibiotic regimen. The patients treated with levofloxacin did not develop hemolytic-uremic syndrome. However, the occurrence rate of hemolytic-uremic syndrome was statistically insignificant between the two groups of patients. There was no association between oral levofloxacin therapy and the risk of hemolytic-uremic syndrome in patients included in the study. Geerdes-Fenge et al. [390] reported that the treatment with ciprofloxacin reduced the risk of hemolytic-uremic syndrome versus other antibiotics (cefotaxime, amoxicillin, and/or metronidazole) in 24 patients with enterohemorrhagic *Escherichia coli*-associated diarrhea.

In a review article, Kakoullis et al. [391] included several studies with infection produced by Shiga toxin-producing *Escherichia coli* O157:H7 to assess the association of FQs with the development of the hemolytic-uremic syndrome. In clinical studies, FQs therapy leads to beneficial results, although unfavorable results were reported in in vitro studies. Recently, Mody et al. [392] suggested that the assessed antimicrobial classes for treating O157 diarrhea should be avoided, especially β-lactams, which increased the odds of the hemolytic-uremic syndrome. Targeted classes of antibiotics were β-lactams, FQs, nitroimidazoles (metronidazole), macrolides, and sulfonamides (sulfamethoxazole-trimethoprim). The treatment with FQs was associated with decreased odds.

From the previously cited studies, it can be seen that FQs therapy was not associated with hemolytic-uremic syndrome. Instead, most of the time, the treatment with FQs had positive results in patients with *Escherichia coli* O157 infection.

#### 5.15.1. Underlying Mechanisms of Severe Hemolytic-Uremic Syndrome

Known data plead for immune hemolytic anemia produced by temafloxacin as a secondary event to the immune complex formation [49]. By the observations of Maguire et al. [393], the “temafloxacin syndrome” was immune-mediated in some patients. The chemical structure of temafloxacin with a 2,4-difluorophenyl substituent at the N1 position was speculated to be responsible for this severe adverse reaction. Additionally, the increased number of fluorine atoms could be responsible, but it is still an unfounded hypothesis [100]. In patients with *Escherichia coli*-associated diarrhea treated with antibiotics associated with the hemolytic-uremic syndrome, production and the release of toxic seems to be the primary mechanism [392].

#### 5.15.2. Severe Hemolytic-Uremic Syndrome Associated with Modern FQs

The structural similarity of delafloxacin with temafloxacin is easy to notice. At the N1 position, temafloxacin has a 2,4-difluorophenyl substituent and delafloxacin, a 6-amino-3,5-difluoropyridine substituent, combined with a chlorine substituent at the C8 position. Nevertheless, delafloxacin has a good safety profile, and no reported cases of hemolytic-uremic syndrome were induced by delafloxacin [21,100]. To the extent of our knowledge, clinical studies did not report the occurrence of the severe hemolytic-uremic syndrome as a treatment-emergent adverse event for the modern FQs reviewed herein.

### 5.16. Acute Renal Failure

Intensive care unit patients often develop acute renal failure associated with a higher mortality risk. A large number of drugs can produce impairment of renal function in patients admitted to intensive care units. Mechanisms vary from decreased glomerular filtration to interstitial nephritis, acute tubular necrosis, or crystallization within the tubules. Some antibiotics (including FQs) can cause interstitial nephritis [227]. Generally, older FQs are eliminated by metabolism and renal clearance. Newer FQs are non-renal clearance drugs, e.g., moxifloxacin. The pharmacokinetics of moxifloxacin are not significantly affected by renal or hepatic impairment [8,394].

Mulgaonkar et al. [395] identified the specific transporters of FQs in renal proximal tubule cells. These data are essential at the molecular level to explain the mechanism of drug–drug interactions, organ-specific side-effects, and interpatient variability, considering the FQs’ pharmacokinetics and pharmacodynamics. Numerous members from the transporter superfamilies (ATP-binding cassette and the solute carrier transporters) have an active role in FQs’ disposition, especially at the renal level. Lomaestro [396] conducted a Medline search of eleven FQs regimens to highlight the incidence of FQ-induced nephrotoxicity during the 1985–1999 period. At that time, the incidence of nephrotoxicity associated with FQs therapy was difficult to estimate. Of the FQs targeted, ciprofloxacin appears to increase the risk, but this finding may be due to its frequent use versus newer FQs. It is known that renal function declines with age, and some FQs are eliminated by the kidney (e.g., ofloxacin, levofloxacin). Recommendations for modifying their doses are based on the renal function changes and not on the age category, and doses need to be reduced if the creatinine clearance parameter is reduced. The nephrotoxicity associated with FQs therapy is rare but can endanger the patient’s life. Some identified predisposing factors are age over 60, recent nephrotoxic drugs or co-administration with such drugs, and the patient’s reduced fluid intake [397].

The first reported data which support a significantly increased risk of acute kidney injury associated with oral FQs therapy were published by Bird et al. [398]. They conducted a case-control analysis from 2001 to 2011 to study the association between oral FQs therapy and acute kidney injury in male patients. The identified FQs in the regimens were ciprofloxacin, gatifloxacin, gemifloxacin, levofloxacin, moxifloxacin, and norfloxacin. A significantly increased risk of acute kidney injury with oral FQs’ use (RR 2.16, 95% CI 1.52–3.18) (significant) resulted from the study. In addition, ciprofloxacin was associated with a higher risk compared with levofloxacin. Additionally, they examined the interaction with renin–angiotensin system blockers and found a significant interaction.

Farid et al. [399] conducted a retrospective study of biopsy-proven FQ-induced acute interstitial nephritis (1993–2016). As expected, ciprofloxacin was the most prescribed (in 17 patients out of 24). Acute interstitial nephritis induced by FQs therapy could be considered rare. The symptoms, the time of symptom onset, and the degree of nephrotoxicity were different for each patient. In addition, the clinicians faced a significant challenge in treating acute interstitial nephritis due to the lack of extrarenal manifestations in most patients, and renal biopsy may be necessary for diagnosis. Discontinuation of the FQs therapy led to renal function recovery within three weeks in most patients.

#### 5.16.1. Underlying Mechanisms of Acute Renal Failure

Renal injury may be due to a hypersensitivity reaction or a direct toxic effect of secondary FQs’ administration [396]. Most of the case reports were attributed to several main mechanisms of kidney injury induced by FQs therapy: hemolytic-uremic syndrome, interstitial nephritis, and crystal-induced nephropathy [399]. The evidence of FQ-induced nephrotoxicity was an allergic or hypersensitivity reaction, termed acute interstitial nephritis, granulomatous interstitial nephritis, tubular necrosis, or crystalluria (crystals’ formation consecutive to FQs therapy) [396,398].

Older FQs (e.g., ciprofloxacin, norfloxacin) can induce nephrotoxicity by crystallization in the renal tubules due to their lower solubility in an environment with neutral or alkaline pH [397,400]. In a case report described by Khan et al. [401], ciprofloxacin precipitated intra-tubular due to alkaline urine and caused renal failure. Intrarenal crystallization of ciprofloxacin may occur secondary to administering large doses in elderly patients, in patients with chronic kidney disease, dehydration, or urine alkalinization [401,402]. It is known that the solubility of ciprofloxacin is pH-dependent: the maximum solubility is at pH < 5, and the minimum solubility is near pH 7 (the isoelectric point) [403,404,405].

Strong interactions with the co-administration of FQs and renin–angiotensin system blockers were found by Bird et al. [398]. Blockers of the renin–angiotensin system cause dilation of the efferent arteriole, affecting the kidney. Thus, the intraglomerular pressure is reduced, and the serum creatinine level increases. The risk of acute kidney injury induced by renin–angiotensin system blockers may increase after renal failure (with dehydration or other drug administration). Consequently, the study authors recommended avoiding the co-administration of these two classes of drugs.

#### 5.16.2. Acute Renal Failure Associated with Modern FQs

To the extent of our knowledge, clinical studies report insufficient data regarding the signs related to acute renal failure induced by the new FQs reviewed herein.

Delafloxacin. There is no data concerning acute renal failure associated with delafloxacin [406,407,408]. Renal failure was identified as potentially due to therapy in one patient in the delafloxacin group in a phase 3, multicenter, randomized, double-blind, active-controlled study comparing the efficacy and safety of delafloxacin to vancomycin/aztreonam in patients with ABSSSI. In contrast, three vancomycin/aztreonam patients experienced renal failure [207].

Lascufloxacin. No cases of renal failure associated with lascufloxacin treatment were reported. Notably, during a phase 1 clinical investigation in healthy Japanese males, laboratory tests revealed modestly elevated levels of blood creatinine but no changes in renal function markers. As a result, it was determined that the increase in blood creatinine levels was not caused by a structural defect in the kidneys [356].

Nemonoxacin. Two single-dose, randomized crossover studies conducted in twenty-four healthy male Chinese volunteers reported two episodes of a creatine phosphokinase increase as possibly related to the investigational drug. However, these adverse events were classified as mild and unrelated to renal failure [342].

For levonadifloxacin, sitafloxacin, and zabofloxacin, no cases of renal failure associated with this newer FQs treatment were reported.

## 6. Management of Side-Effects

Essential guidelines for managing AEs related to FQs are provided below based on the sources evaluated in this review.

1. Discontinuation of the regimen

If a patient develops severe side-effects associated with an FQ, the treatment should be stopped, and the FQ should be replaced with an antibiotic from a different class. There is an exception to continuing the treatment with the respective FQ only if the benefits outweigh the risks [32,35,65]. Some situations in which the treatment discontinuation is recommended are presented as follows.

Treatment with FQs must be stopped immediately if the patient experiences any sign or symptoms related to tendinopathy/tendon rupture (instability and impairment in walking, redness, swelling, stiffness, pain, inflammation, or rupture of a tendon). Drug administration should be stopped at the first sign related to tendinopathy/tendon rupture, and patients should avoid exercise and use of the affected area [32,33,34,35,196,197]. In addition, prescribing FQs in patients with a history of tendinopathy/tendon rupture should be avoided [152].

Moreover, in patients with FQ-induced acute renal failure and other forms of nephrotoxicity (such as allergic interstitial nephritis), treatment requires immediate discontinuation of the FQ agent [396]. Management of allergic reactions to FQs, such as ciprofloxacin, moxifloxacin, levofloxacin, and delafloxacin, requires prompt treatment discontinuation. Additionally, corticosteroid therapy, electrolyte fluid replacement, and albumin substitution are usually needed to resolve the allergic reaction [270]. Along with clinicians, pharmacists are responsible for advising patients to discontinue the treatment with FQs if they report any severe side-effects [8].

2. Rest and decrease of physical load on the tendon alongside physical therapy

Following a diagnosis of tendinopathy or tendon rupture related to an FQ regimen and discontinuation of the offending drug, therapy should involve rest and reducing physical demand on the tendon(s). Additionally, physical therapy should be started. In the case of the elderly, strict bed rest with an attendant may be required. The injured tendon(s) should be protected for the first month (heel lift, counterforce bracing, and crutches). Physical load on the Achilles tendon must be reduced for six weeks to six months, and tendinitis recovery might take anywhere from a few weeks to two months [196].

3. Avoiding FQs regimens in patients with pre-existing conditions of aortic aneurysm or aortic dissection

The use of FQs has been linked to an increased incidence of aortic aneurysm or dissection. Alternative antibiotics to FQs should be sought in individuals with a history of valvular regurgitation, high blood pressure, certain hereditary or uncommon conditions (e.g., Marfan syndrome, Ehlers–Danlos syndrome, Takayasu arteritis, Behcet’s disease), and giant cell arteritis, as well as in elderly patients [69,79,91,172,173].

4. Reducing the duration of FQs’ administration may decrease the risk of peripheral neuropathy side-effect occurrence

In a nested case-control study, Morales et al. [230] concluded that peripheral neuropathy risk increases by approximately 3% for each additional day of FQs regimens. Thus, they suggested that shorter FQs regimens could reduce the risk of peripheral neuropathy. Currently, there are no guidelines for treating peripheral neuropathy induced by FQs. In a case report, a twenty-year-old male with diabetes mellitus type 1 developed severe painful peripheral neuropathy on the second day of ten days of treatment with levofloxacin. The i.v. treatment with immunoglobulin was initiated alongside several analgesic drugs, and the pain was reduced. Then, the patient received outpatient therapy bimonthly with i.v. immunoglobulin following a slow recovery [233].

5. Monitoring the serum folate level and supplementation in FQ-induced neuropathy

One pathological biomarker was highlighted in patients with peripheral neuropathy: the reduction of serum folate. Popescu [231] proposed the measure and the supplementation with folate in patients with FQ-induced neuropathy based on a case report.

6. Avoiding the FQs therapy in patients with a history of seizures

In patients who have experienced past seizures, FQs should be used with caution. Co-administration with other medicines known to lower the seizure threshold should also be avoided [248]. However, a self-controlled case series study that analyzed data retrieved from clinical centers in Hong Kong and the UK did not support an association between the use of oral FQs and the incidence of seizures among patients with a history of seizures. The study’s results indicate that the infection for which the oral FQ was prescribed may be responsible for the development of incident seizures rather than the drug itself [215].

7. Collaboration between the health professional team to prevent and treat hypersensitive syndrome to FQs

Before administration of any FQ, predictive tests for FQ-induced hypersensitivity should be performed. The selection of the appropriate therapy for the patient is based on the analysis of risk factors for the patient’s sensitivity to FQs, quantitative assessment using laboratory tests, early identification of the toxicity mechanism, and possible drug interactions. The treatment of hypersensitivity induced by FQs and its prevention can be positively influenced by collaboration between clinicians, pharmacists, and laboratory doctors [275].

Doña et al. [277,280,281] compiled a diagnostic algorithm of hypersensitivity AEs induced by FQs and proposed approaches and solutions for treating these AEs. Sometimes, the diagnosis can be confirmed only by a drug provocation test, a slightly risky procedure for the patient [278]. An essential aspect of managing hypersensitivity to FQs is avoiding the culprit drug [277,280,281]. There are three directions to follow in the management of allergy induced by FQs, as follows: (1) discontinuation of the causative drug, (2) initiation of an alternative regimen (from another class of antibiotics, if possible), and (3) supportive care (e.g., corticosteroid therapy, electrolytes as fluid replacement, albumin substitution, histamine antagonist, anti-IgE antibody, short-acting beta-adrenergic agonist), depending on the severity of the events [270,278]. Some severe delayed skin reactions require wound care procedures or surgical debridement [270]. Desensitization is necessary in cases where FQ is the only treatment option [270,277,278,280,281].

8. Avoiding exposure to sunlight

During FQs therapy, the patients should avoid exposure to sunlight due to the phototoxic potential of FQs [195,297]. The concentration of FQs and the dose of UV radiation influence the degree of phototoxic damage, with the most severe phototoxic reactions occurring after high or moderate sunlight exposure [409,410].

9. Increase the *Clostridium difficile* infection control measures

According to Donskey [319], lowering FQs’ use is an essential measure in the management of *Clostridium difficile*, as well as resistant Gram-negative bacteria, and infection control measures take precedence over FQs therapy. On the other hand, Weiss [312] and Deshpande et al. [314] believe that the current information on the link between FQs therapy and *Clostridium difficile* infection is “suggestive, but not conclusive”. Furthermore, improved infection control techniques resulted in a significant reduction in the rate of *Clostridium difficile* infections across all impacted regions. Furthermore, identified risk variables for developing primary *Clostridium difficile* infections in diverse inpatient populations aged over 65 comprise antibiotic misuse and prior hospitalization [314,317]. Other factors include gastric acid suppressants, nonselective NSAIDs, and certain comorbidities [316].

10. Reversing severe induced hypoglycemia by FQs

Hypoglycemia associated with using FQs can be severe, even life-threatening. Several studies have indicated that octreotide can reverse severe sulfonylurea-induced hypoglycemia [411,412,413]. For example, Kelesidis et al. [346] have successfully used octreotide in hypoglycemia associated with ciprofloxacin in a 65-year-old woman with type 2 diabetes (treated with glipizide), chronic kidney disease, and cirrhosis.

11. Replacing the culprit drug for the elevation of hepatic transaminases

Notable here is a reported case in which a 45-year-old woman presented increased hepatic transaminase values following ciprofloxacin therapy. When ciprofloxacin was replaced with levofloxacin, hepatic transaminase values returned to normal. In addition, at the second admission (later in the same month), the prescribed levofloxacin did not induce the elevation of hepatic transaminases [362].

12. Combating potential crystalluria produced by FQs in the kidney

The i.v. hydration and maintaining the pH of the acidic urine can fight crystalluria if these measures are applied in time. Microscopic examination of urine sediment for diagnoses of FQ-induced acute kidney injury is a simple and non-invasive method compared to kidney biopsy [401].

13. Avoiding the co-administration of FQs and renin–angiotensin system blockers

The risk of acute renal failure increases by 4.5-fold in men taking fluoroquinolone antibiotics in combination with renin–angiotensin system blockers [398]. Therefore, to decrease the risk of acute kidney injury, Bird et al. [398] recommend avoiding the association between FQs and renin–angiotensin system blockers. The risk of death from severe infections outweighs the risks associated with FQs therapy. However, the potential for acute kidney injury increases the importance of judicious FQs’ prescribing.

14. Lower doses of FQs in older patients with advanced chronic kidney disease

In a recent study, Muanda et al. [414] recommended lower doses of FQs in patients with advanced chronic kidney disease. Higher-than-recommended FQs doses were associated with several severe side-effects (nervous system and/or psychiatric disorders, collagen-associated disorders, and hypoglycemia). However, the absolute risk of these side-effects resulting from this study was less than 2%.

Pharmacists and clinicians are essential in ensuring patients’ safety with systemic FQs. They should be aware of the latest information about this class of antibiotics. Additionally, they must be able to suggest alternative therapies when the situation requires it. In addition, pharmacists have an essential role in counselling patients about the benefits and potential risks of FQs. If adverse events occur, it is necessary to collaborate with patients to submit reports to regulatory authorities [121].

## 7. Rational Use and Caution of Modern FQs

In the 21st century, the problem of balancing the advantages and disadvantages of FQs in therapy is increasingly raised. For example, Van Bambeke et al. [167] compiled a list of important pros and cons for FQs’ clinical administration. They focused on the use in the clinics by the main indications of FQs. Additionally, they highlighted the main side-effects of FQs, the frequency observed based on the published studies until that moment, and the populations at risk. In 2016, the German Society for Infectious Diseases, in association with other societies, associations, and institutions, developed a guideline for the rational use of antibiotics (including FQs) in hospitals. This guide aimed to decrease antimicrobial resistance, increase the beneficial clinical outcomes in patients with various infections, and decrease the toxicity induced by antibiotic therapy. Excessive use of FQs and cephalosporins in hospitals should be reconsidered. Thus, restricting some antibiotics (e.g., FQs, cephalosporins) or substituting some antibiotic classes (e.g., penicillin) proved to reduce the incidence of *Clostridium difficile* infection. A decrease in the prescription of FQs and cephalosporins in hospitals may reduce the incidence of infections produced by multidrug-resistant bacteria [415].

The warnings of the drug regulatory agencies FDA and EMA were discussed in Section 3.4. Targeted side-effects involve tendinitis, tendon rupture, aortic dissections or ruptures of an aortic aneurysm, heart valve regurgitation/incompetence, muscles, joints, peripheral neuropathy, nerves, CNS, dysglycemia, and mental health disorders (Table 2). Additionally, the FDA and EMA recommend restriction of FQs’ use due to severe, disabling, and potentially permanent side-effects with systemic FQs antibiotics or those administered by inhalation [32,35]. In addition, FQs have designated antibiotics in patients with various infections who have no other treatment alternatives [91]. Prescription of systemic FQs should be avoided for mild and moderate infections (e.g., viral infections, influenza, the common cold, acute bronchitis, and pharyngotonsillitis) if there are other equally effective antibiotics. Systemic FQs are recommended if other alternatives are not available. Additionally, systemic FQs are recommended if the pathogen agent that caused the infection is multidrug-resistant (e.g., multidrug-resistant tuberculosis) or in case of clinical/microbiologic failure. Another particular situation is the severe allergy to beta-lactams of the patient (a life-threatening event). Therefore, documented justification of the prescription of systemic FQs is necessary, as well as informing patients about risks and benefits [165].

Based on the reported studies, numerous cautions regarding the use of FQs have been issued. Some precautions based on relevant studies are listed below.

In patients with intra-abdominal infections, the FQs are widely prescribed due to their spectrum of activity, including Gram-negative pathogens and good tissue penetration. Usually, FQs are co-administered with metronidazole (except moxifloxacin) to widen the spectrum of activity against anaerobe bacteria. Thus, ciprofloxacin and levofloxacin are no longer included in the first-line medication for empiric treatment of intra-abdominal infections due to the prevalence of FQs resistance. In general, the threatening increase in bacterial resistance to FQs worldwide (e.g., *Escherichia coli* and other Enterobacteriaceae) has restricted the use of FQs for empirical therapy of intra-abdominal infections. Therefore, clinicians must avoid prescribing broad-spectrum antibiotics to save them for future use. In addition, the need for antibiotic therapy must be reassessed daily, and judicious antibiotic management decisions must be integrated into responsible prescribing behavior [416]. Peripheral neuropathy associated with FQs regimens may have an increased relative incidence in patients with diabetes mellitus type 1, alcohol abuse, increasing body mass index (BMI), smoking patients, and in patients treated with oral phenytoin or nitrofurantoin [230,233]. Thus, caution is necessary when administering FQs to these categories of patients.

Sellick et al. [242] conducted a study to determine the incidence of FQs (systemic ciprofloxacin, levofloxacin, or moxifloxacin) therapy association with delirium or psychosis and the risk factors in a veteran population. A higher risk of developing psychosis or delirium was found in hospitalized older patients and hospitalized patients with typically prescribed antipsychotics. In conclusion, the study’s authors advise caution when administering FQs to hospitalized patients from any of these two categories. A study conducted by Scavone et al. [234] in Italy, based on individual case safety reports (2001–2019) and data from the online public report system (2002–2019), found that the third generation of FQs was linked to a higher reporting probability of AEs (musculoskeletal, neurological, and psychiatric) comparative to the second generation of FQs. Inappropriate prescribing of FQs is one of the leading causes of Aes. Rational use of FQs can result in the best clinical care and lower risks of Aes and bacterial resistance.

All FQs (including the newer representatives) should be used with caution in patients with a history of or suspected CNS conditions or in the presence of risk factors that may predispose them to seizures or reduce the seizure threshold [152,154]. Additionally, all FQs are not recommended in patients with myasthenia gravis or with a known history of myasthenia gravis due to post-marketing of serious AEs, including death and the requirement for ventilator support [74,152,154]. Due to the cross-reactivity within the FQs class, strict avoidance of all FQs is necessary for patients with FQ-induced allergies [272]. Another caution regarding FQs therapy (moxifloxacin especially) concerns patients with a genetic predisposition of QT interval prolongation or a prolonged baseline QT interval, hypokalemia, and other QT prolonging medication. Moxifloxacin is not recommended to treat infections in these patients due to the associated high risk of QTc interval prolongation and torsade de pointes. Patients must be carefully monitored if there is no alternative treatment [329]. Thus, avoid the concomitant administration of FQs with a drug known for prolonging QT intervals, such as ondansetron, antipsychotics, antidepressants, methadone, and azole antifungals [323,326].

Due to the potential to produce dysglycemia, FQs should be prescribed with caution in diabetic patients who have comorbidities and are on antidiabetic and/or steroid treatment [235,348]. In addition, before initiation of FQs therapy, the patient’s liver function would be indicated to be assessed due to the hepatotoxicity potential of FQs [364,365,366,417]. Additionally, the FQs should be prescribed judiciously only when no alternatives exist. It is challenging to prevent induced hepatotoxicity, and the treatment regimens for drug-induced liver injury are limited [393]. Additionally, caution and dose reduction are necessary for prescribing FQs to elderly patients with advanced chronic kidney disease. In a population-based cohort study in Ontario, Canada, 11,907 subjects 66 years or older with advanced chronic kidney disease were enrolled, with 5482 (46.0%) receiving a higher-dose FQ (ciprofloxacin, 501–1000 mg/day; levofloxacin, 501–750 mg/day; norfloxacin, 401–800 mg/day) and 6435 (54.0%) receiving a lower-dose FQ (ciprofloxacin, 500 mg/day; levofloxacin, 250–500 mg/day; norfloxacin, 400 mg/day). The conclusions of this study indicate that older patients with advanced chronic kidney disease who received FQs at a higher-than-recommended dose were significantly more likely to have a hospital visit involving nervous system and/or psychiatric disorders, hypoglycemia, or a collagen-associated event, even though the absolute risk of these events was less than 2% [414].

It is necessary to use FQs therapy with caution in patients with diabetes mellitus and alcohol abuse to prevent the occurrence of retinal detachment. In contrast to diabetes, alcohol abuse was not associated with an increased retinal detachment risk in the study population or demographic subgroups. Women were shown to be more at risk than men, while African Americans were found to be more at risk than Caucasians. There was also a five-fold risk in the youngest-age tertile (0–55), which decreased and became non-significant in the higher-age tertiles [222].

A recent meta-analysis published by Yan et al. [418] compared the efficacy and safety of FQs with other drug comparators (sulfamethoxazole-trimethoprim, nitrofurantoin, fosfomycin, and β-lactams) in adult patients with uncomplicated UTI. The meta-analysis was based on 47 randomized controlled trials, including 8992 patients. In treating adult patients with uncomplicated UTI, it was discovered that FQs positively affected clinical remission and bacteriological eradication compared to the other comparator medications. Bacteriological resistance to FQs and the recurrence rate were relatively low. Six randomized controlled trials reported severe AEs, such as vomiting, retrosternal burning and acid regurgitation, episodes of depression, and allergic reactions. However, FQs therapy did not show a higher risk of AEs than drug comparators.

The adaptation speed of bacteria is higher than the launching speed of new antibiotics on the pharmaceutical market. Therefore, the judicious use is more than necessary for the representatives of the new generations of FQs to preserve these drugs as valuable antibiotics and not as discoveries of the past [165,167,415,416,419].

## 8. Conclusions

A broad antibacterial spectrum, including activity against anaerobic bacteria, characterized the newer FQs. The transition from one generation to another meant the acquisition of compounds with a broader spectrum of activity, improved pharmacokinetic properties, and reduced AEs. An essential advantage is that a series of resistant bacteria are susceptible to the new FQs. The most reported severe AEs for the approved FQs include tendon rupture (especially to Achilles tendon), arthralgia, tendonitis, pain in extremities, gait disturbance, neuropathies associated with paresthesia, fatigue, memory impairment, depression, sleep disorders, impaired vision, hearing, taste and smell, phototoxicity, genotoxicity, QTc prolongation, hematological effect, hepatic eosinophilia effect, pulmonary interstitial eosinophilia, immunological side-effects, hypoglycemia, and CYP 450 inhibition. In addition, due to some severe AEs from those listed, many compounds have been withdrawn from therapy.

The modern FQs reviewed herein (delafloxacin, lascufloxacin, levonadifloxacin, nemonoxacin, sitafloxacin, and zabofloxacin) have a proven safety profile. To our knowledge, clinical studies did not report the occurrence of severe aortic aneurysms, aortic dissection, treatment-related tendinitis, tendon rupture, or myopathy, retinal detachment, neuropsychiatric toxicity, severe hemolytic-uremic syndrome, or acute renal failure induced by the modern FQs reviewed herein. Sporadic cases of peripheral neuropathy for delafloxacin were reported in some clinical studies. In the few existing studies, treatment with nemonoxacin was associated with some signs of peripheral neuropathy (headache, dizziness, and facial/muscle twitch). Some clinical studies revealed mild cutaneous side-effects and hypersensitivity reactions related to modern FQs: skin and subcutaneous tissue disorders, such as pruritus, urticaria, dermatitis, rash (delafloxacin), rash (lascufloxacin), skin papule (levonadifloxacin), pruritus, and rash (nemonoxacin). In a clinical study, one patient in the two phase 3 trials (0.1%) developed a *Clostridium difficile* infection in the delafloxacin group. This TEAE was classified as mild in severity and did not cause the discontinuation of the treatment. The existing studies concerning QT prolongation as an AE suggest the administration of nemonoxacin with caution, especially in high doses requiring careful monitoring. The associated risk of prolonging the QT interval seems similar to levofloxacin. In some clinical studies, hyperglycemia was observed as an AE, with the reported symptoms being mild or moderate in severity. In clinical trials, delafloxacin was not associated with phototoxicity or increased risk of treatment-related phototoxicity, while levonadifloxacin was found with weak phototoxicity comparable with levofloxacin.

Rational use and caution of modern FQs take into consideration the limitation of the AEs. Prescription of systemic FQs should be avoided for mild and moderate infections if there are other equally effective antibiotics. Systemic FQs are recommended if all other alternatives are unavailable, if the pathogen agent that caused the infection is multidrug-resistant, in case of clinical/microbiologic failure, or in the particular situation of the severe allergy to beta-lactams. Documented justification of the prescription of systemic FQs is necessary, as well as informing patients about risks and benefits.

Caution is necessary when the risk of peripheral neuropathy associated with FQs regimens may have increased: in patients with diabetes mellitus type 1, alcohol abuse, increasing body mass index (BMI), smoking patients, and in patients treated with oral phenytoin or nitrofurantoin therapy. A higher risk of developing psychosis or delirium in hospitalized older patients and in hospitalized patients with typically prescribed antipsychotics was found in one study, which advises caution when delivering FQs to hospitalized patients from any of these two categories. All FQs (including the newer representatives) should be used with caution in patients with a history of or suspected CNS conditions or in the presence of risk factors that may predispose them to seizures or reduce the seizure threshold. Additionally, all FQs are not recommended in patients with myasthenia gravis or with a known history of myasthenia gravis due to post-marketing severe AEs, including death and the requirement for ventilator support. Another caution regarding FQs therapy (moxifloxacin especially) concerns patients with a genetic predisposition to QT interval prolongation or a prolonged baseline QT interval, hypokalemia, and other QT prolonging medication. Thus, moxifloxacin is not recommended to treat infections in these patients due to the associated high risk of QTc interval prolongation and torsade de pointes. Patients must be carefully monitored if there is no alternative treatment. Additionally, caution and dose reduction are necessary for prescribing FQs to elderly patients with advanced chronic kidney disease. It is required to use FQs therapy with caution in patients with diabetes mellitus to prevent the occurrence of retinal detachment.

Management of the AEs associated with FQs’ administration includes measures that depend on the type and severity of the AEs, such as discontinuation of the regimen, rest, and decrease of physical load on the tendon, alongside physical therapy, avoiding FQs regimens in patients with pre-existing conditions of aortic aneurysm or aortic dissection, reducing the duration of FQs administration that may decrease the risk of peripheral neuropathy side-effects, monitoring the serum folate level and supplementation in FQ-induced neuropathy, avoiding FQs therapy in patients with a history of seizures, increasing the *Clostridium difficile* infection control measures, combating potential crystalluria produced by FQs in the kidney, avoiding co-administration of FQs and renin–angiotensin system blockers, and administering lower doses of FQs in older patients with advanced chronic kidney disease.

Although FQs are a valuable class of antibiotics, they require judicious prescribing and numerous precautions due to the potential of associated severe AEs. So far, new FQs discussed herein have presented an acceptable safety profile. However, similar to the old representatives, these new FQs should be cautiously administered only when necessary, and there are no antibiotic alternatives until future studies reinforce a superior safety profile.

## Figures and Tables

**Table 3:** Examples of FQs withdrawn from therapy due to severe AEs (AEs—adverse effects, FQs—fluoroquinolones).

No.	FQs (Generation)	Manufacturer	Approval Year	Withdrawn Year	Side-Effects	References
1	Fleroxacin (2nd)	Kyorin Pharmaceutical	1981	1990	CNS effects, phototoxicity	[37,93,94]
2	Tosufloxacin (2nd)	Toyama Chemical	1990	2006	Thrombocytopenia, nephritis, toxic epidermal necrosis, eosinophilic pneumonitis	[37,95,96,97]
3	Temafloxacin (2nd)	Abbott Laboratories	1992	1992	“Temafloxacin syndrome”: hemolytic-uremic syndrome	[2,37,49,98,99,100]
4	Lomefloxacin (2nd)	Serle	1992	1993	CNS effects, phototoxicity	[101,102,103]
5	Sparfloxacin (3rd)	Mylan	1996	2001	QT prolongation, phototoxicity	[103,104,105,106,107,108]
6	Alatrofloxacin (3rd)	Pfizer	1997	2006	Seizures, thrombocytopenia, hepatotoxicity	[108,109,110]
7	Trovafloxacin (3rd)	Pfizer	1997	2000	Hepatotoxicity	[99,108,111,112]
8	Grepafloxacin (3rd)	Glaxo	1997	1999	QT prolongation, fatal cardiotoxicity, gastrointestinal toxicity	[99,108,113,114]
9	Clinafloxacin (3rd)	Parke Davis	1999	1999	Hypoglycemia, phototoxicity	[115]
10	Gatifloxacin (3rd)	Bristol-Myers Squibb	1999	2006	Increased risk of dysglycemia	[108,116,117]
11	Gemifloxacin (3rd)	Vansen Pharma	1999	2009	Rash erythematous	[117,118,119]

**Table 4:** Essential information about modern FQs introduced in therapy (ABSSSI—acute bacterial skin and skin-structure infections, CAP—community-acquired pneumonia, COPD—chronic obstructive pulmonary disease, FQs—fluoroquinolones, G(+)—Gram-positive, G(−)—Gram-negative, MRSA—methicillin-resistant *Staphylococcus aureus*, QNs—antibacterial quinolones, Ref.—References).

Approval Year(s)	QNs/FQs	Antibacterial Spectrum	Indications	Formulations	Administration	Observations	Ref.
1998 2000	Nadifloxacin	G(+) (including MRSA and coagulase-negative staphylococci), G(−), and anaerobic bacteria	Acne vulgaris Other skin infections	Topical (1% cream)	Twice daily	Approved in Japan Approved by EMA	[42,43,122,123,127]
2008 2012	Sitafloxacin	Broad-spectrum: G(+) and G(−) bacteria, including anaerobic bacteria, atypical pathogens (particularly against bacteria resistant to other FQs)	Respiratory infections and UTI	Oral formulation (tablets 50 mg)	50–100 mg twice daily	Approved in Japan Approved in Thailand	[128,129,130,131,132,133,134,135,136,137,138,139,140]
2009	Besifloxacin	*Staphylococcus aureus, Streptococcus pneumoniae, Staphylococcus epidermidis, Moraxella catarrhalis, Hemophilus influenzae, Corynebacterium* spp.	Bacterial conjunctivitis	Ophthalmic suspension (0.6%)	One drop in the affected eye(s), three times daily, 4 to 12 h apart (for 7 days)	Approved by the FDA Approved in Canada and later in other countries (Argentina, South Korea, Brazil etc.)	[141,142,143]
2014	Finafloxacin	Broad-spectrum (particularly against *Staphylococcus aureus* and *Pseudomonas aeruginosa*)	Acute otitis externa	Otic suspension (0.3%)	Four drops in the affected ear(s), twice daily (for seven days)	Approved by the FDA	[27,144,145,146]
2014 2016	Nemonoxacin ^1^	Broad spectrum: typical and atypical respiratory pathogens (particularly against resistant G(+) cocci, including penicillin-resistant *Streptococcus pneumoniae* and MRSA)	CAP (pending for diabetic foot ulcer infections, Skin and soft tissue infections approval)	Oral formulation (capsules 250 mg) Intravenous (i.v.) formulation	500 mg once a day	Approved in Taiwan Approved in China	[28,29,147,148]
2015	Zabofloxacin	Broad-spectrum (particularly against major respiratory tract pathogens)	Acute bacterial exacerbation of COPD	Oral (tablets)	367 mg once daily (for five days)	Approved in South Korea, the Middle East, and North-African countries FDA’s Clinical phase 3 trial approval for CAP patients	[21,149,150,151]
2017 2019	Delafloxacin	Broad-spectrum: G(+) (including MRSA) and G(−) bacteria	ABSSSI, CAP	Oral (tablets) Parenteral (i.v. infusion)	Oral: 450 mg every 12 h (for 5 to 14 days) Parenteral: 300 mg by i.v. infusion over 60 min every 12 h.	Approved by the FDA Approved by the EMA	[21,152,153,154,155]
2017 2018 2019	Ozenoxacin	*Staphylococcus aureus*, *Staphylococcus pyogenes*, and other G(+) bacteria sensitive and resistant to methicillin, QNs, mupirocin and fusidic acid	Impetigo	Topical (1% cream)	Twice daily (for five days)	Approved by the FDA Approved in Spain Approved in 12 EU countries	[126,156,157,158]
2019 2022	Lascufloxacin	Major respiratory tract pathogens *(*e.g., *Streptococcus pneumoniae, Moraxella catarrhalis, Hemophilus influenzae,* and *Mycoplasma pneumoniae)*	Respiratory tract and ear, nose, and throat infections, CAP, otorhinolaryngological infections	Oral (tablets)	75 mg once daily	Approved in Japan Approved in China	[23,159,160,161]
2020	Levonadifloxacin	Broad-spectrum: G(+) (including MRSA and FQs-resistant *Staphylococcus aureus*) and G(−) bacteria, atypical bacteria, anaerobic bacteria, bioterror pathogens	ABSSSI with concurrent bacteraemia and diabetic foot infections	Oral (tablets) I.v. injection	500 mg twice daily 800 mg twice daily	Approved in India	[162,163,164]

^1^ Non-fluorinated QN.

**Table 5:** Relationships between chemical structure and side-effects of FQs (FQs—fluoroquinolones, GABA—gamma-aminobutyric acid, NSAIDs—non-steroidal anti-inflammatory drugs, Ref.—References).

No.	Substituents on Chemical Structure (*X and Y: C or N)* 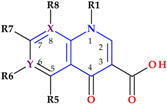	Associated Side-Effects	Ref.
1	N1: Cyclopropyl, Ethyl, 2,4-Diphluorophenyl	Interactions with the cytochrome P450	[167]
	N1: Cyclopropyl ≥ Ethyl > 2,4-Diphluorophenyl > Fluorethyl	Theophylline interactions	[168]
	N1: Cyclopropyl ≥ Tert-Butyl > 2,4-Diphluorophenyl > Ethyl	Genotoxicity	[168]
	N1: 2,4-Diphluorophenyl (combined with halogen at position 8)	Phototoxicity	[169]
	N1: Cyclopropyl, Ethyl	Phototoxicity	[169]
	N1: Aminodifluorophenyl, 1-Isoxazolyl	Phototoxicity	[169]
2	C2: No substitution	No side-effects	[168,170]
3	C3: Carboxyl	Decrease absorption of biological metals; interactions with antacids, multimineral supplements, and milk products (due to metal binding and chelating)	[168]
	C3: Carboxyl	Chondrotoxicity (due to specific chelation of Mg^2+^)	[167,170]
4	C4: Oxo	Decrease absorption of biological metals; interactions with antacids, multimineral supplements, and milk products (due to metal binding and chelating)	[168]
	C4: Oxo	Chondrotoxicity (due to specific chelation of Mg^2+^)	[167,170]
5	C5: Methyl/amino substituents	QT prolongation	[104,168,170]
5	C5: Methyl >> H > Amino substituents	Phototoxicity	[167,168]
5	C5: Methyl > Amino substituents > H	Genotoxicity	[167,168]
6	C6: Fluorine substituent	Genotoxicity	[167]
7	C7: Pyrrolidine > Piperazine > Alkyl	Genotoxicity	[167]
	C7: Pyrrolidine (unsubstituted) > Piperazine (unsubstituted) > Pyrrolidine (substituted) > Piperazine (substituted)	Genotoxicity	[168]
	C7: Alkyl > Piperazine (unsubstituted) > Pyrrolidine (unsubstituted) > Piperazine (substituted) or Pyrrolidine (substituted)	Neuropsychiatric toxicity, seizures (GABA receptor binding)	[8,44,117,167,168,171]
	C7: Piperazine (unsubstituted)	Crystalluria	[167]
	C7: Piperazine (unsubstituted) > Pyrrolidine (unsubstituted) > Piperazine (substituted) or Pyrrolidine (substituted)	Some NSAIDs interactions	[167]
	C7: Pyrrolidine (unsubstituted) > Piperazine (unsubstituted) > Piperazine (substituted) or Pyrrolidine (substituted)	Theophylline interactions	[168]
8	C8: Fluorine/chlorine substituents (F > Cl)	Phototoxicity, Genotoxicity	[167,170]
	C-F > C-Cl > N > C-H > C-O-Methyl, C-CF_3_	Phototoxicity	[168]
	C-F > C-Cl ≥ C-O-Methyl > N > C-H	Genotoxicity	[168]
	C8: Fluorine/chlorine, methoxy substituents	Crystalluria	[167]
	C8: Bulky substituent	Decreased interactions with the cytochrome P450	[167]
	N8: Naphtiridone nucleus	Increased interactions with the cytochrome P450	[167,170]

## Data Availability

Not applicable.

## Supplementary Materials

The following supporting information can be downloaded at: https://www.mdpi.com/article/10.3390/pharmaceutics15030804/s1, Table S1: Notable AEs of novel FQs (AEs—adverse effects, ALT—alanine aminotransferase, AST—aspartate aminotransferase, b.i.d.—two times, COPD—chronic obstructive pulmonary disease, FQs—fluoroquinolones, i.v.—intravenous, qd—once a day, TEAE—treatment-emergent adverse event, Ref.—references, UTI—urinary tract infections).

## References

[1] Beale J.M., Block J.H. (2010). Wilson and Gisvold’s Textbook of Organic Medicinal and Pharmaceutical Chemistry.

[2] Ball P. (2000). Quinolone Generations: Natural History or Natural Selection?. J. Antimicrob. Chemother..

[3] Bush N.G., Diez-Santos I., Abbott L.R., Maxwell A. (2020). Quinolones: Mechanism, Lethality and Their Contributions to Antibiotic Resistance. Molecules.

[4] Rusu A., Lungu I.-A., Moldovan O.-L., Tanase C., Hancu G. (2021). Structural Characterization of the Millennial Antibacterial (Fluoro)Quinolones—Shaping the Fifth Generation. Pharmaceutics.

[5] Sheehan G., Chew N.S.Y., Ronald A.R., Low D.E. (2003). The History of Quinolones. Fluoroquinolone Antibiotics.

[6] Lesher G.Y., Froelich E.J., Gruett M.D., Bailey J.H., Brundage R.P. (1962). 1,8-Naphthyridine Derivatives. A New Class of Chemotherapeutic Agents. J. Med. Pharm. Chem..

[7] MacDougall C., Brunton L.L., Hilal-Dandan R., Knollmann B.C. (2017). Sulfonamides, Trimethoprim-Sulfamethoxazole, Quinolones, and Agents for Urinary Tract Infections. Goodman & Gilman’s: The Pharmacological Basis of Therapeutics.

[8] Pham T.D.M., Ziora Z.M., Blaskovich M.A.T. (2019). Quinolone Antibiotics. Medchemcomm.

[9] Tillotson G.S. (1996). Quinolones: Structure-Activity Relationships and Future Predictions. J. Med. Microbiol..

[10] Peterson L.R. (2001). Quinolone Molecular Structure-Activity Relationships: What We Have Learned about Improving Antimicrobial Activity. Clin. Infect. Dis..

[11] Domagala J.M., Hagen S.E. (2003). Structure-Activity Relationships of the Quinolone Antibacterials in the New Millennium: Some Things Change and Some Do Not. Quinolone Antimicrobial Agents.

[12] Srinivasan S., Beema Shafreen R.M., Nithyanand P., Manisankar P., Pandian S.K. (2010). Synthesis and in Vitro Antimicrobial Evaluation of Novel Fluoroquinolone Derivatives. Eur. J. Med. Chem..

[13] Asif M. (2015). Study of Antimicrobial Quinolones and Structure Activity Relationship of Anti-Tubercular Compounds. Res. Rev. J. Chem..

[14] Brighty K.E., Gootz T.D., Andriole V.T. (2000). Chapter 2—Chemistry and Mechanism of Action of the Quinolone Antibacterials. The Quinolones.

[15] Aldred K.J., Kerns R.J., Osheroff N. (2014). Mechanism of Quinolone Action and Resistance. Biochemistry.

[16] Hooper D.C. (2000). Mechanisms of Action and Resistance of Older and Newer Fluoroquinolones. Clin. Infect. Dis..

[17] Hooper D.C., Jacoby G.A. (2016). Topoisomerase Inhibitors: Fluoroquinolone Mechanisms of Action and Resistance. Cold Spring Harb. Perspect. Med..

[18] Correia S., Poeta P., Hébraud M., Capelo J.L., Igrejas G. (2017). Mechanisms of Quinolone Action and Resistance: Where Do We Stand?. J. Med. Microbiol..

[19] Blondeau J.M. (2004). Fluoroquinolones: Mechanism of Action, Classification, and Development of Resistance. Surv. Ophthalmol..

[20] Fàbrega A., Madurga S., Giralt E., Vila J. (2009). Mechanism of Action of and Resistance to Quinolones. Microb. Biotechnol..

[21] Kocsis B., Gulyás D., Szabó D. (2021). Delafloxacin, Finafloxacin, and Zabofloxacin: Novel Fluoroquinolones in the Antibiotic Pipeline. Antibiotics.

[22] Polat H.K., Pehlivan S.B., Ozkul C., Calamak S., Ozturk N., Aytekin E., Firat A., Ulubayram K., Kocabeyoglu S., Irkec M. (2020). Development of Besifloxacin HCl Loaded Nanofibrous Ocular Inserts for the Treatment of Bacterial Keratitis: In Vitro, Ex Vivo and in Vivo Evaluation. Int. J. Pharm..

[23] Thakare R., Singh S., Dasgupta A., Chopra S. (2020). Lascufloxacin Hydrochloride to Treat Bacterial Infection. Drugs Today.

[24] Hagihara M., Kato H., Shibata Y., Sakanashi D., Asai N., Suematsu H., Yamagishi Y., Mikamo H. (2021). In Vivo Pharmacodynamics of Lascufloxacin and Levofloxacin against Streptococcus Pneumoniae and Prevotella Intermedia in a Pneumonia Mixed-Infection Mouse Model. Anaerobe.

[25] Totoli E.G., Nunes Salgado H.R. (2018). Besifloxacin: A Critical Review of Its Characteristics, Properties, and Analytical Methods. Crit. Rev. Anal. Chem..

[26] DeCory H.H., Sanfilippo C.M., Proskin H.M., Blondeau J.M. (2020). Characterization of Baseline Polybacterial versus Monobacterial Infections in Three Randomized Controlled Bacterial Conjunctivitis Trials and Microbial Outcomes with Besifloxacin Ophthalmic Suspension 0.6%. PLoS ONE.

[27] McKeage K. (2015). Finafloxacin: First Global Approval. Drugs.

[28] Poole R.M. (2014). Nemonoxacin: First Global Approval. Drugs.

[29] Chang L.-W., Hsu M.-C., Zhang Y.-Y. (2019). Nemonoxacin (Taigexyn^®^): A New Non-Fluorinated Quinolone.

[30] Cheng S.-L., Wu R.-G., Chuang Y.-C., Perng W.-C., Tsao S.-M., Chang Y.-T., Chang L.-W., Hsu M.-C. (2019). Integrated Safety Summary of Phase II and III Studies Comparing Oral Nemonoxacin and Levofloxacin in Community-Acquired Pneumonia. J. Microbiol. Immunol. Infect..

[31] Yan A., Bryant E.E. (2022). Quinolones. StatPearls.

[32] Office of the Commissioner FDA Updates Warnings for Fluoroquinolone Antibiotics. https://www.fda.gov/news-events/press-announcements/fda-updates-warnings-fluoroquinolone-antibiotics.

[33] Gatti M., Bianchin M., Raschi E., De Ponti F. (2020). Assessing the Association between Fluoroquinolones and Emerging Adverse Drug Reactions Raised by Regulatory Agencies: An Umbrella Review. Eur. J. Intern. Med..

[34] Francisco E.M. Fluoroquinolone and Quinolone Antibiotics: PRAC Recommends New Restrictions on Use Following Review of Disabling Potentially Long-Lasting Side Effects. https://www.ema.europa.eu/en/news/fluoroquinolone-quinolone-antibiotics-prac-recommends-new-restrictions-use-following-review.

[35] Francisco E.M. Disabling and Potentially Permanent Side Effects Lead to Suspension or Restrictions of Quinolone Fluoroquinolone Antibiotics. https://www.ema.europa.eu/en/news/disabling-potentially-permanent-side-effects-lead-suspension-restrictions-quinolone-fluoroquinolone.

[36] Dassault Systèmes BIOVIA Draw for Academics. https://discover.3ds.com/biovia-draw-academic.

[37] Rubinstein E. (2001). History of Quinolones and Their Side Effects. CHE.

[38] Lungu I.-A., Moldovan O.-L., Biriș V., Rusu A. (2022). Fluoroquinolones Hybrid Molecules as Promising Antibacterial Agents in the Fight against Antibacterial Resistance. Pharmaceutics.

[39] Singh C.L., Singh A., Kumar S., Majumdar D.K. (2014). Besifloxacin the fourth generation fluoroquinolone: A review. J. Drug Deliv. Ther..

[40] Tanaka K., Vu H., Hayashi M. (2021). In Vitro Activities and Spectrum of Lascufloxacin (KRP-AM1977) against Anaerobes. J. Infect. Chemother..

[41] Bhatia A., Mastim M., Shah M., Gutte R., Joshi P., Kumbhar D., Periasamy H., Palwe S.R., Chavan R., Bhagwat S. (2020). Efficacy and Safety of a Novel Broad-Spectrum Anti-MRSA Agent Levonadifloxacin Compared with Linezolid for Acute Bacterial Skin and Skin Structure Infections: A Phase 3, Openlabel, Randomized Study. J. Assoc. Physicians India.

[42] Sweetman S.C. (2009). Martindale: The Complete Drug Reference.

[43] Narayanan V., Motlekar S., Kadhe G., Bhagat S. (2014). Efficacy and Safety of Nadifloxacin for Bacterial Skin Infections: Results from Clinical and Post-Marketing Studies. Dermatol. Ther..

[44] Limberakis C. (2007). Quinolone Antibiotics: Levofloxacin (Levaquin^®^), Moxifloxacin (Avelox^®^), Gemifloxacin (Factive^®^), and Garenoxacin (T-3811). The Art of Drug Synthesis.

[45] Cervantes L.J., Mah F.S. (2011). Clinical Use of Gatifloxacin Ophthalmic Solution for Treatment of Bacterial Conjunctivitis. Clin. Ophthalmol..

[46] Drug Approval Package: Zymar (Gatifloxacin) NDA #021493. https://www.accessdata.fda.gov/drugsatfda_docs/nda/2003/021493_Zymar.cfm.

[47] Mah F.S., Sanfilippo C.M. (2016). Besifloxacin: Efficacy and Safety in Treatment and Prevention of Ocular Bacterial Infections. Ophthalmol. Ther..

[48] Domagala J.M. (1994). Structure-Activity and Structure-Side-Effect Relationships for the Quinolone Antibacterials. J. Antimicrob. Chemother..

[49] Blum M.D., Graham D.J., McCloskey C.A. (1994). Temafloxacin Syndrome: Review of 95 Cases. Clin. Infect. Dis..

[50] Mogle B.T., Steele J.M., Thomas S.J., Bohan K.H., Kufel W.D. (2018). Clinical Review of Delafloxacin: A Novel Anionic Fluoroquinolone. J. Antimicrob. Chemother..

[51] Bassetti M., Hooper D., Tillotson G. (2019). Analysis of Pooled Phase 3 Safety Data for Delafloxacin in Acute Bacterial Skin and Skin Structure Infections. Clin. Infect. Dis..

[52] Suaifan G.A.R.Y., Mohammed A.A.M. (2019). Fluoroquinolones Structural and Medicinal Developments (2013–2018): Where Are We Now?. Bioorganic Med. Chem..

[53] Lu T., Zhao X., Li X., Drlica-Wagner A., Wang J.-Y., Domagala J., Drlica K. (2001). Enhancement of Fluoroquinolone Activity by C-8 Halogen and Methoxy Moieties: Action against a Gyrase Resistance Mutant of Mycobacterium Smegmatis and a Gyrase-Topoisomerase IV Double Mutant of Staphylococcus Aureus. Antimicrob. Agents Chemother..

[54] Thomas G. (2008). Medicinal Chemistry: An Introduction.

[55] Haas W., Sanfilippo C.M., Hesje C.K., Morris T.W. (2013). Contribution of the R8 Substituent to the in Vitro Antibacterial Potency of Besifloxacin and Comparator Ophthalmic Fluoroquinolones. Clin. Ophthalmol..

[56] Drlica K., Hiasa H., Kerns R., Malik M., Mustaev A., Zhao X. (2009). Quinolones: Action and Resistance Updated. Curr. Top Med. Chem..

[57] Cheng G., Hao H., Dai M., Liu Z., Yuan Z. (2013). Antibacterial Action of Quinolones: From Target to Network. Eur. J. Med. Chem..

[58] Redgrave L.S., Sutton S.B., Webber M.A., Piddock L.J.V. (2014). Fluoroquinolone Resistance: Mechanisms, Impact on Bacteria, and Role in Evolutionary Success. Trends Microbiol..

[59] Malik M., Zhao X., Drlica K. (2006). Lethal Fragmentation of Bacterial Chromosomes Mediated by DNA Gyrase and Quinolones. Mol. Microbiol..

[60] Hong Y., Zeng J., Wang X., Drlica K., Zhao X. (2019). Post-Stress Bacterial Cell Death Mediated by Reactive Oxygen Species. Proc. Natl. Acad. Sci. USA.

[61] Hong Y., Li Q., Gao Q., Xie J., Huang H., Drlica K., Zhao X. (2020). Reactive Oxygen Species Play a Dominant Role in All Pathways of Rapid Quinolone-Mediated Killing. J. Antimicrob. Chemother..

[62] Rodríguez-Rosado A.I., Valencia E.Y., Rodríguez-Rojas A., Costas C., Galhardo R.S., Blázquez J., Rodríguez-Beltrán J. (2018). Reactive Oxygen Species Are Major Contributors to SOS-Mediated Mutagenesis Induced by Fluoroquinolones. bioRxiv.

[63] Michalak K., Sobolewska-Włodarczyk A., Włodarczyk M., Sobolewska J., Woźniak P., Sobolewski B. (2017). Treatment of the Fluoroquinolone-Associated Disability: The Pathobiochemical Implications. Oxidative Med. Cell. Longev..

[64] Roberts J.R. (2018). InFocus: Fluoroquinolone Side Effects Just Got Scarier. Emerg. Med. News.

[65] EMA Quinolone- and Fluoroquinolone-Containing Medicinal Products. https://www.ema.europa.eu/en/medicines/human/referrals/quinolone-fluoroquinolone-containing-medicinal-products.

[66] Center for Drug Evaluation and Research FDA Drug Safety Communication: FDA Updates Warnings for Oral and Injectable Fluoroquinolone Antibiotics Due to Disabling Side Effects. FDA 2019. https://www.fda.gov/drugs/drug-safety-and-availability/fda-drug-safety-communication-fda-updates-warnings-oral-and-injectable-fluoroquinolone-antibiotics.

[67] Aschenbrenner D.S. (2016). The FDA Revises Boxed Warning For Fluoroquinolones-Again. Am. J. Nurs..

[68] Center for Drug Evaluation and Research FDA Reinforces Safety Information about Serious Low Blood Sugar Levels and Mental Health Side Effects with Fluoroquinolone Antibiotics; Requires Label Changes. FDA 2018. https://www.fda.gov/drugs/drug-safety-and-availability/fda-reinforces-safety-information-about-serious-low-blood-sugar-levels-and-mental-health-side.

[69] Office of the Commissioner FDA In Brief: FDA Warns That Fluoroquinolone Antibiotics Can Cause Aortic Aneurysm in Certain Patients. FDA 2019. https://www.fda.gov/news-events/fda-brief/fda-brief-fda-warns-fluoroquinolone-antibiotics-can-cause-aortic-aneurysm-certain-patients.

[70] EMA Meeting Highlights from the Pharmacovigilance Risk Assessment Committee (PRAC) 1–4 October 2018. https://www.ema.europa.eu/en/news/meeting-highlights-pharmacovigilance-risk-assessment-committee-prac-1-4-october-2018.

[71] Tanne J.H. (2008). FDA Adds “Black Box” Warning Label to Fluoroquinolone Antibiotics. BMJ.

[72] Waknine Y. Fluoroquinolones Earn Black Box Warning for Tendon-Related Adverse Effects. https://www.medscape.com/viewarticle/577302.

[73] Center for Drug Evaluation and Research Postmarket Drug Safety Information for Patients and Providers—Information for Healthcare Professionals: Fluoroquinolone Antimicrobial Drugs [Ciprofloxacin (Marketed as Cipro and Generic Ciprofloxacin), Ciprofloxacin Extended-Release (Marketed as Cipro XR and Proquin XR), Gemifloxacin (Marketed as Factive), Levofloxacin (Marketed as Levaquin), Moxifloxacin (Marketed as Avelox), Norfloxacin (Marketed as Noroxin), and Ofloxacin (Marketed as Floxin)]. http://wayback.archive-it.org/7993/20161022101528/http://www.fda.gov/Drugs/DrugSafety/PostmarketDrugSafetyInformationforPatientsandProviders/ucm126085.htm.

[74] Jones S.C., Sorbello A., Boucher R.M. (2011). Fluoroquinolone-Associated Myasthenia Gravis Exacerbation. Drug Saf..

[75] Center for Drug Evaluation and Research Drug Safety and Availability—FDA Drug Safety Communication: FDA Requires Label Changes to Warn of Risk for Possibly Permanent Nerve Damage from Antibacterial Fluoroquinolone Drugs Taken by Mouth or by Injection. http://wayback.archive-it.org/7993/20161022101530/http://www.fda.gov/Drugs/DrugSafety/ucm365050.htm.

[76] Center for Drug Evaluation and Research FDA Drug Safety Communication: FDA Advises Restricting Fluoroquinolone Antibiotic Use for Certain Uncomplicated Infections; Warns about Disabling Side Effects That Can Occur Together. FDA 2016. https://www.fda.gov/drugs/drug-safety-and-availability/fda-drug-safety-communication-fda-advises-restricting-fluoroquinolone-antibiotic-use-certain.

[77] FDA Drug Safety Communication FDA Warns about Increased Risk of Ruptures or Tears in the Aorta Blood Vessel with Fluoroquinolone Antibiotics in Certain Patients. https://www.fda.gov/drugs/drug-safety-and-availability/fda-warns-about-increased-risk-ruptures-or-tears-aorta-blood-vessel-fluoroquinolone-antibiotics.

[78] EMA Systemic and Inhaled Fluoroquinolones: Risk of Heart Valve Regurgitation/Incompetence. https://www.ema.europa.eu/en/medicines/dhpc/systemic-inhaled-fluoroquinolones-risk-heart-valve-regurgitationincompetence.

[79] Etminan M., Sodhi M., Ganjizadeh-Zavareh S., Carleton B., Kezouh A., Brophy J.M. (2019). Oral Fluoroquinolones and Risk of Mitral and Aortic Regurgitation. J. Am. Coll. Cardiol..

[80] Guzzardi D.G., Teng G., Kang S., Geeraert P.J., Pattar S.S., Svystonyuk D.A., Belke D.D., Fedak P.W.M. (2019). Induction of Human Aortic Myofibroblast-Mediated Extracellular Matrix Dysregulation: A Potential Mechanism of Fluoroquinolone-Associated Aortopathy. J. Thorac. Cardiovasc. Surg..

[81] Systemic and Inhaled Fluoroquinolones: Small Risk of Heart Valve Regurgitation; Consider Other Therapeutic Options First in Patients at Risk. https://www.gov.uk/drug-safety-update/systemic-and-inhaled-fluoroquinolones-small-risk-of-heart-valve-regurgitation-consider-other-therapeutic-options-first-in-patients-at-risk.

[82] Strange J.E., Holt A., Blanche P., Gislason G., Torp-Pedersen C., Christensen D.M., Hansen M.L., Lamberts M., Schou M., Olesen J.B. (2021). Oral Fluoroquinolones and Risk of Aortic or Mitral Regurgitation: A Nationwide Nested Case-Control Study. Eur. Heart J..

[83] Tandan M., Cormican M., Vellinga A. (2018). Adverse Events of Fluoroquinolones vs. Other Antimicrobials Prescribed in Primary Care: A Systematic Review and Meta-Analysis of Randomized Controlled Trials. Int. J. Antimicrob. Agents.

[84] Hook E.W., Golden M.R., Taylor S.N., Henry E., Tseng C., Workowski K.A., Swerdlow J., Nenninger A., Cammarata S. (2019). Efficacy and Safety of Single-Dose Oral Delafloxacin Compared With Intramuscular Ceftriaxone for Uncomplicated Gonorrhea Treatment: An Open-Label, Noninferiority, Phase 3, Multicenter, Randomized Study. Sex Transm Dis.

[85] Kurono Y., Kawauchi H., Hori S., Tateda K., Totsuka K., Asano M., Suzuki K. (2020). Phase III Double-Blind Comparative Study of Lascufloxacin versus Levofloxacin in Patients with Sinusitis. Jpn. J. Chemother..

[86] Health Canada Summary Safety Review—Oral Fluoroquinolones—Assessing the Potential Risk of Retinal Detachment. https://www.canada.ca/en/health-canada/services/drugs-health-products/medeffect-canada/safety-reviews/summary-safety-review-oral-fluoroquinolones-assessing-potential-risk-retinal.html.

[87] Health Canada Summary Safety Review—Fluoroquinolones—Assessing the Potential Risk of Persistent and Disabling Side Effects. https://www.canada.ca/en/health-canada/services/drugs-health-products/medeffect-canada/safety-reviews/summary-safety-review-fluoroquinolones-assessing-potential-risk-persistent-disabling-effects.html.

[88] Medicines and Healthcare products Regulatory Agency Fluoroquinolone Antibiotics: New Restrictions and Precautions for Use Due to Very Rare Reports of Disabling and Potentially Long-Lasting or Irreversible Side Effects. https://www.gov.uk/drug-safety-update/fluoroquinolone-antibiotics-new-restrictions-and-precautions-for-use-due-to-very-rare-reports-of-disabling-and-potentially-long-lasting-or-irreversible-side-effects.

[89] Cheng A.C., Turnidge J., Collignon P., Looke D., Barton M., Gottlieb T. (2012). Control of Fluoroquinolone Resistance through Successful Regulation, Australia. Emerg. Infect. Dis..

[90] Therapeutic Goods Administration (TGA) Fluoroquinolone Antibiotics and Risk of Aortic Aneurysm/Dissection. https://www.tga.gov.au/news/safety-updates/fluoroquinolone-antibiotics-and-risk-aortic-aneurysmdissection.

[91] Therapeutic Goods Administration (TGA) Update—Fluoroquinolone Antibiotics and Adverse Events. https://www.tga.gov.au/news/safety-updates/update-fluoroquinolone-antibiotics-and-adverse-events.

[92] Outterson K., Powers J.H., Seoane-Vazquez E., Rodriguez-Monguio R., Kesselheim A.S. (2013). Approval and Withdrawal of New Antibiotics and Other Antiinfectives in the U.S., 1980–2009. J. Law. Med. Ethics.

[93] Bowie W.R., Willetts V., Jewesson P.J. (1989). Adverse Reactions in a Dose-Ranging Study with a New Long-Acting Fluoroquinolone, Fleroxacin. Antimicrob. Agents Chemother..

[94] Geddes A.M. (1993). Safety of Fleroxacin in Clinical Trials. Am. J. Med..

[95] Kimura N., Miyazaki E., Matsuno O., Abe Y., Tsuda T. (1998). Drug-induced pneumonitis with eosinophilic infiltration due to tosufloxacin tosilate. J. Jpn. Respir. Soc..

[96] Choi M.K., Woo H.Y., Heo J., Cho M., Kim G.H., Song G.A., Kim M.B. (2011). Toxic Epidermal Necrolysis Associated with Sorafenib and Tosufloxacin in a Patient with Hepatocellular Carcinoma. Ann. Dermatol..

[97] Owens R.C., Ambrose P.G. (2005). Antimicrobial Safety: Focus on Fluoroquinolones. Clin. Infect. Dis..

[98] Aronson J.K. (2015). Meyler’s Side Effects of Drugs: The International Encyclopedia of Adverse Drug Reactions and Interactions.

[99] Mandell L.A., Ball P., Tillotson G. (2001). Antimicrobial Safety and Tolerability: Differences and Dilemmas. Clin. Infect. Dis..

[100] Finch R.G. (1993). The Withdrawal of Temafloxacin. Drug Saf..

[101] Young A.R., Fakouhi T.D., Harrison G.I., Roniker B., Swabb E.A., Hawk J.L.M. (1996). The UVR Wavelength Dependence for Lomefloxacin Photosensitization of Human Skin. J. Photochem. Photobiol. B Biol..

[102] Lowe N.J., Fakouhi T.D., Stern R.S., Bourget T., Roniker B., Swabb E.A. (1994). Photoreactions with a Fluoroquinolone Antimicrobial: Evening versus Morning Dosing. Clin. Pharmacol. Ther..

[103] Petersen U. (2006). Quinolone Antibiotics: The Development of Moxifloxacin. Analogue-Based Drug Discovery.

[104] Jaillon P., Morganroth J., Brumpt I., Talbot G. (1996). Overview of Electrocardiographic and Cardiovascular Safety Data for Sparfloxacin. Sparfloxacin Safety Group. J. Antimicrob. Chemother..

[105] Rubinstein E. (1996). Safety Profile of Sparfloxacin in the Treatment of Respiratory Tract Infections. J. Antimicrob. Chemother..

[106] Lipsky B.A., Dorr M.B., Magner D.J., Talbot G.H. (1999). Safety Profile of Sparfloxacin, a New Fluoroquinolone Antibiotic. Clin. Ther..

[107] King D.E., Malone R., Lilley S.H. (2000). New Classification and Update on the Quinolone Antibiotics. Am. Fam. Physician.

[108] Qureshi Z.P., Seoane-Vazquez E., Rodriguez-Monguio R., Stevenson K.B., Szeinbach S.L. (2011). Market Withdrawal of New Molecular Entities Approved in the United States from 1980 to 2009. Pharmacoepidemiol. Drug Saf..

[109] Melvani S., Speed B.R. (2000). Alatrofloxacin-Induced Seizures during Slow Intravenous Infusion. Ann. Pharmacother..

[110] Gales B.J., Sulak L.B. (2000). Severe Thrombocytopenia Associated with Alatrofloxacin. Ann. Pharmacother..

[111] File T.M., Schlemmer B., Garau J., Cupo M., Young C., The 049 Clinical Study Group (2001). Efficacy and Safety of Gemifloxacin in the Treatment of Community-Acquired Pneumonia: A Randomized, Double-Blind Comparison with Trovafloxacin. J. Antimicrob. Chemother..

[112] Pannu H.K., Gottlieb L., Fishman E.K. (2001). Acute Liver Failure Due to Trovafloxacin: CT Findings. Emerg. Radiol..

[113] Stahlmann R., Schwabe R. (1997). Safety Profile of Grepafloxacin Compared with Other Fluoroquinolones. J. Antimicrob. Chemother..

[114] Anderson M.E., Mazur A., Yang T., Roden D.M. (2001). Potassium Current Antagonist Properties and Proarrhythmic Consequences of Quinolone Antibiotics. J. Pharmacol. Exp. Ther..

[115] Zhanel G.G., Walkty A., Vercaigne L., Karlowsky J.A., Embil J., Gin A.S., Hoban D.J. (1999). The New Fluoroquinolones: A Critical Review. Can. J. Infect. Dis..

[116] FDA Determination That TEQUIN (Gatifloxacin) Was Withdrawn From Sale for Reasons of Safety or Effectiveness. https://www.federalregister.gov/documents/2008/09/09/E8-20938/determination-that-tequin-gatifloxacin-was-withdrawn-from-sale-for-reasons-of-safety-or.

[117] Mandell L., Tillotson G. (2002). Safety of Fluoroquinolones: An Update. Can. J. Infect. Dis..

[118] EMA Questions and Answers on the Withdrawal of the Marketing Authorisation Application for Factive Gemifloxacin. https://www.ema.europa.eu/en/documents/medicine-qa/questions-answers-withdrawal-marketing-authorisation-application-factive-gemifloxacin_en.pdf.

[119] EMA Menarini International Operations Luxembourg Withdraws Its Marketing Authorisation Application for Factive (Gemifloxacin). https://www.ema.europa.eu/en/news/menarini-international-operations-luxembourg-withdraws-its-marketing-authorisation-application.

[120] Center for Drug Evaluation and Research CDER Division of Drug Information. https://www.fda.gov/about-fda/center-drug-evaluation-and-research-cder/cder-division-drug-information.

[121] Molnar D.M., Kremzner M.E. (2019). Fluoroquinolones: A Hot Topic for Pharmacists and the Food and Drug Administration’s Division of Drug Information. J. Am. Pharm. Assoc..

[122] Nenoff P. (2006). Acne Vulgaris and Bacterial Skin Infections: Review of the Topical Quinolone Nadifloxacin. Expert Rev. Dermatol..

[123] EMA EMA/150639/2017 Nadifloxacin, List of Nationally Authorised Medicinal Products. https://www.ema.europa.eu/en/documents/psusa/nadifloxacin-list-nationally-authorised-medicinal-products-psusa/00002102/201605_en.pdf.

[124] Wetzel C., Lonneman M., Wu C. (2021). Polypharmacological Drug Actions of Recently FDA Approved Antibiotics. Eur. J. Med. Chem..

[125] Center for Drug Evaluation and Research Drug Trial Snapshot: Xepi. FDA 2020. https://www.fda.gov/drugs/drug-approvals-and-databases/drug-trial-snapshot-xepi.

[126] Troy Brown FDA Approves Ozenoxacin Cream for Impetigo. https://www.medscape.com/viewarticle/890180.

[127] Jacobs M.R., Appelbaum P.C. (2006). Nadifloxacin: A Quinolone for Topical Treatment of Skin Infections and Potential for Systemic Use of Its Active Isomer, WCK 771. Expert Opin. Pharmacother..

[128] Anderson D.L. (2008). Sitafloxacin Hydrate for Bacterial Infections. Drugs Today.

[129] Chen C.-K., Cheng I.-L., Chen Y.-H., Lai C.-C. (2020). Efficacy and Safety of Sitafloxacin in the Treatment of Acute Bacterial Infection: A Meta-Analysis of Randomized Controlled Trials. Antibiotics.

[130] NCATS Inxight Drugs—SITAFLOXACIN. https://drugs.ncats.io/drug/9TD681796G.

[131] Kawada Y., Ishihara S., Matsui T., Tsugawa M., Matsumoto T. (2008). Clinical Study of Sitafloxacin in Febrile Complicated Pyelonephritis. Jpn. J. Chemother..

[132] Kawada Y., Matsumoto T., Onodera S., Kaku M., Hori S. (2008). Clinical Study of Sitafloxacin in Male Nongonococcal Urethritis. Jpn. J. Chemother..

[133] Onodera S., Hori S. (2008). Clinical Study of Sitafloxacin in the Treatment of Male Gonococcal Urethritis. Jpn. J. Chemother..

[134] Matsuda S., Noguchi M., Yasuda J., Hori S. (2008). Clinical Study of Sitafloxacin in Treatment of Cervicitis with Chlamydia Trachomatis. Jpn. J. Chemother..

[135] Kawada Y., Ishihara S., Matsui T., Tsugawa M., Matsumoto T., Watanabe K., Nakashima M. (2008). Comparative Study on Sitafloxacin and Levofloxacin in Complicated Urinary Tract Infections. Jpn. J. Chemother..

[136] Kawada Y., Yasuda M., Tanaka K., Monden K., Akasaka S., Egashira T., Kaku M., Hori S. (2008). Dose-Comparative Study of Sitafloxacin in Complicated Urinary Tract Infections. Jpn. J. Chemother..

[137] Saito A., Tanigawara Y., Watanabe A., Aoki N., Niki Y., Kohno S., Kaku M., Hori S., Totsuka K. (2008). Open Study of Sitafloxacin in Patients with Respiratory Tract Infections. Jpn. J. Chemother..

[138] Sasaki J., Hori S. (2008). Oral Tissue Distribution, Efficacy, and Safety of Sitafloxacin in Patients with Dentistry and Oral Surgery Infection. Jpn. J. Chemother..

[139] Saito A., Watanabe A., Aoki N., Niki Y., Kohno S., Kaku M., Hori S. (2008). Phase III Double-Blind Comparative Study of Sitafloxacin versus Tosufloxacin in Patients with Community-Acquired Pneumonia. Jpn. J. Chemother..

[140] Kobayashi H., Watanabe A., Nakata K., Wada K., Niki Y., Kohno S. (2008). Double-Blind Comparative Study of Sitafloxacin versus Levofloxacin in Patients with Respiratory Tract Infection. Jpn. J. Chemother..

[141] FDA N. 22308/S-013 Besifloxacin Label 2009. https://www.accessdata.fda.gov/drugsatfda_docs/label/2018/022308s013lbl.pdf.

[142] Khimdas S., Visscher K.L., Hutnik C.M.L. (2011). Besifloxacin Ophthalmic Suspension: Emerging Evidence of Its Therapeutic Value in Bacterial Conjunctivitis. Ophthalmol. Eye Dis..

[143] Therapeutic Goods Administration (TGA) AusPAR: Besifloxacin Hydrochloride. https://www.tga.gov.au/resources/auspar/auspar-besifloxacin-hydrochloride.

[144] FDA Drug Approval Package Xtoro (Finafloxacin) Otic Suspension. https://www.accessdata.fda.gov/drugsatfda_docs/nda/2014/206307Orig1s000TOC.cfm.

[145] Barnes K.B., Zumbrun S.D., Halasohoris S.A., Desai P.D., Miller L.L., Richards M.I., Russell P., Bentley C., Harding S.V. (2019). Demonstration of the Broad-Spectrum In Vitro Activity of Finafloxacin against Pathogens of Biodefense Interest. Antimicrob. Agents Chemother..

[146] Barnes K.B., Richards M., Laws T.R., Nunez A., Thwaite J.E., Bentley C., Harding S. (2021). Finafloxacin Is an Effective Treatment for Inhalational Tularemia and Plague in Mouse Models of Infection. Antimicrob. Agents Chemother..

[147] Product-Taigexyn®-TaiGen Biotechnology—A Pharmaceutical Company Dedicating in Drug Discovery. https://www.taigenbiotech.com/en/product/detail/Taigexyn.

[148] Cao G., Zhang J., Zhang Y., Guo B., Yu J., Wu X., Chen Y., Wu J.-F., Shi Y. (2014). Safety, Tolerability, and Pharmacokinetics of Intravenous Nemonoxacin in Healthy Chinese Volunteers. Antimicrob. Agents Chemother..

[149] Kocsis B., Szabo D. (2016). Zabofloxacin for Chronic Bronchitis. Drugs Today.

[150] Dongwha News Dong Wha Pharm’s Quinolone Antibacterial Agent, “Zabolante,” Wins at the 19th KNDA. https://www.dong-wha.co.kr/english/customer/dnews/content.asp?t_idx=1139.

[151] Dong Wha Pharmaceutical CO., LTD Antibiotics. https://www.dong-wha.co.kr/english/product/content.asp?t_idx=545&t_page=1&d=&b=10&s=11.

[152] FDA Baxdela (Delafloxacin) Tablets and Injection. https://www.accessdata.fda.gov/drugsatfda_docs/nda/2017/208610Orig1s000,208611Orig1s000TOC.cfm.

[153] Scott L.J. (2020). Delafloxacin: A Review in Acute Bacterial Skin and Skin Structure Infections. Drugs.

[154] EMA Quofenix. https://www.ema.europa.eu/en/medicines/human/EPAR/quofenix.

[155] Eudaley S. (2018). Delafloxacin (Baxdela) for Skin Infections. Am. Fam. Physician.

[156] FDA XEPITM (Ozenoxacin) Cream, for Topical Use 2017. https://www.accessdata.fda.gov/drugsatfda_docs/label/2017/208945lbl.pdf.

[157] Garcia Ron G., Villa Arranz M. (2022). New Therapeutic Applications of Ozenoxacin in Superficial Skin Infections. Dermatol. Rep..

[158] Torrelo A., Grimalt R., Masramon X., Albareda López N., Zsolt I. (2020). Ozenoxacin, a New Effective and Safe Topical Treatment for Impetigo in Children and Adolescents. Dermatology.

[159] Ogihara S. (2019). NHI Drug Price Listing and Release of Oral Quinolone Antibacterial Agent “Lasvic®Tablets 75mg”. https://www.kyorin-pharm.co.jp/en/news/a329f0ae64024c1173f40660eede0efb37f1cbb0.pdf.

[160] Ogihara S., Liang X. KYORIN and Nanjing Neiwa Faith Signed License Agreement for Lascufloxacin in China. 2022, 1. https://www.kyorin-pharm.co.jp/en/news/KYORIN%20and%20Nanjing%20Neiwa%20Faith%20Signed%20License%20Agreement%20for%20Lascufloxacin%20in%20China.pdf.

[161] Tateda K., Tanioka S., Totsuka K., Kohno S. (2020). An Overview of Oral Lascufloxacin, a Novel Quinolone Antibiotic. Jpn. J. Chemother..

[162] Bakthavatchalam Y.D., Shankar A., Muniyasamy R., Peter J.V., Marcus Z., Triplicane Dwarakanathan H., Gunasekaran K., Iyadurai R., Veeraraghavan B. (2020). Levonadifloxacin, a Recently Approved Benzoquinolizine Fluoroquinolone, Exhibits Potent in Vitro Activity against Contemporary Staphylococcus Aureus Isolates and Bengal Bay Clone Isolates Collected from a Large Indian Tertiary Care Hospital. J. Antimicrob. Chemother..

[163] Saxena D., Kaul G., Dasgupta A., Chopra S. (2020). Levonadifloxacin Arginine Salt to Treat MRSA Infection and Acute Bacterial Skin and Skin Structure Infection. Drugs Today.

[164] Koulenti D., Xu E., Song A., Sum Mok I.Y., Karageorgopoulos D.E., Armaganidis A., Tsiodras S., Lipman J. (2020). Emerging Treatment Options for Infections by Multidrug-Resistant Gram-Positive Microorganisms. Microorganisms.

[165] Richards G.A., Brink A.J., Feldman C. (2019). Rational Use of the Fluoroquinolones. S. Afr. Med. J..

[166] Kuula L.S.M., Viljemaa K.M., Backman J.T., Blom M. (2019). Fluoroquinolone-Related Adverse Events Resulting in Health Service Use and Costs: A Systematic Review. PLoS ONE.

[167] Van Bambeke F., Michot J.-M., Van Eldere J., Tulkens P.M. (2005). Quinolones in 2005: An Update. Clin. Microbiol. Infect..

[168] Lipsky B.A., Baker C.A. (1999). Fluoroquinolone Toxicity Profiles: A Review Focusing on Newer Agents. Clin. Infect. Dis..

[169] Hayashi N., Nakata Y., Yazaki A. (2004). New Findings on the Structure-Phototoxicity Relationship and Photostability of Fluoroquinolones with Various Substituents at Position 1. Antimicrob. Agents Chemother..

[170] Emami S., Shafiee A., Foroumadi A. (2005). Quinolones: Recent Structural and Clinical Developments. Iran. J. Pharm. Res..

[171] Sutter R., Rüegg S., Tschudin-Sutter S. (2015). Seizures as Adverse Events of Antibiotic Drugs: A Systematic Review. Neurology.

[172] Bennett A.C., Bennett C.L., Witherspoon B.J., Knopf K.B. (2019). An Evaluation of Reports of Ciprofloxacin, Levofloxacin, and Moxifloxacin-Association Neuropsychiatric Toxicities, Long-Term Disability, and Aortic Aneurysms/Dissections Disseminated by the Food and Drug Administration and the European Medicines Agency. Expert Opin. Drug Saf..

[173] Lee C.-C., Lee M.G., Hsieh R., Porta L., Lee W.-C., Lee S.-H., Chang S.-S. (2018). Oral Fluoroquinolone and the Risk of Aortic Dissection. J. Am. Coll. Cardiol..

[174] Londhe A.A., Holy C.E., Weaver J., Fonseca S., Villasis A., Fife D. (2021). Risk of Aortic Aneurysm and Dissection Following Exposure to Fluoroquinolones, Common Antibiotics, and Febrile Illness Using a Self-Controlled Case Series Study Design: Retrospective Analyses of Three Large Healthcare Databases in the US. PLoS ONE.

[175] Jun C., Fang B. (2021). Current Progress of Fluoroquinolones-Increased Risk of Aortic Aneurysm and Dissection. BMC Cardiovasc. Disord..

[176] George Sakoulas Adverse Effects of Fluoroquinolones: Where Do We Stand?. https://www.jwatch.org/na48248/2019/02/13/adverse-effects-fluoroquinolones-where-do-we-stand.

[177] Hoefer I.E., den Adel B., Daemen M.J.A.P. (2013). Biomechanical Factors as Triggers of Vascular Growth. Cardiovasc. Res..

[178] Orobello N.C., Dirain C.O., Schultz G., Milne-Davies B.A., Ng M.R.A., Antonelli P.J. (2016). Ciprofloxacin Decreases Collagen in Mouse Tympanic Membrane Fibroblasts. Otolaryngol. Head Neck Surg..

[179] Sendzik J., Shakibaei M., Schäfer-Korting M., Lode H., Stahlmann R. (2010). Synergistic Effects of Dexamethasone and Quinolones on Human-Derived Tendon Cells. Int. J. Antimicrob. Agents.

[180] Guzzardi D., Teng G., Svystonyuk D., Kang S., Park D., Belke D., Turnbull J., Fedak P. (2017). Fluoroquinolone induces human aortic fibroblast-mediated extracellular matrix dysregulation. Can. J. Cardiol..

[181] Uivarosi V. (2013). Metal Complexes of Quinolone Antibiotics and Their Applications: An Update. Molecules.

[182] Walden D.M., Khotimchenko M., Hou H., Chakravarty K., Varshney J. (2021). Effects of Magnesium, Calcium, and Aluminum Chelation on Fluoroquinolone Absorption Rate and Bioavailability: A Computational Study. Pharmaceutics.

[183] Lecomte S., Baron M.H., Chenon M.T., Coupry C., Moreau N.J. (1994). Effect of Magnesium Complexation by Fluoroquinolones on Their Antibacterial Properties. Antimicrob. Agents Chemother..

[184] Akinremi C.A., Obaleye J.A., Amolegbe S.A., Adediji J.F., Bamigboye M.O. (2012). Biological Activities of Some Fluoroquinolones-Metal Complexes. Int. J. Med. Biomed. Res..

[185] Badal S., Her Y.F., Maher L.J. (2015). Nonantibiotic Effects of Fluoroquinolones in Mammalian Cells. J. Biol. Chem..

[186] Shakibaei M., Pfister K., Schwabe R., Vormann J., Stahlmann R. (2000). Ultrastructure of Achilles Tendons of Rats Treated with Ofloxacin and Fed a Normal or Magnesium-Deficient Diet. Antimicrob. Agents Chemother..

[187] Stahlmann R., Kühner S., Shakibaei M., Flores J., Vormann J., van Sickle D.C. (2000). Effects of Magnesium Deficiency on Joint Cartilage in Immature Beagle Dogs: Immunohistochemistry, Electron Microscopy, and Mineral Concentrations. Arch. Toxicol..

[188] Daneman N., Lu H., Redelmeier D.A. (2015). Fluoroquinolones and Collagen Associated Severe Adverse Events: A Longitudinal Cohort Study. BMJ Open.

[189] Yu P.-H., Hu C.-F., Liu J.-W., Chung C.-H., Chen Y.-C., Sun C.-A., Chien W.-C. (2020). The Incidence of Collagen-Associated Adverse Events in Pediatric Population with the Use of Fluoroquinolones: A Nationwide Cohort Study in Taiwan. BMC Pediatr..

[190] Markham A. (2017). Delafloxacin: First Global Approval. Drugs.

[191] Lodise T., Corey R., Hooper D., Cammarata S. (2018). Safety of Delafloxacin: Focus on Adverse Events of Special Interest. Open Forum. Infect. Dis..

[192] Office of the Commissioner FDA Updates Warnings for Fluoroquinolone Antibiotics on Risks of Mental Health and Low Blood Sugar Adverse Reactions. https://www.fda.gov/news-events/press-announcements/fda-updates-warnings-fluoroquinolone-antibiotics-risks-mental-health-and-low-blood-sugar-adverse.

[193] Hornak J.P., Reynoso D. (2022). Early Clinical Experience with Delafloxacin: A Case Series. Am. J. Med. Sci..

[194] Lee A., Lamb Y.N., Shirley M. (2022). Delafloxacin: A Review in Community-Acquired Pneumonia. Drugs.

[195] Stahlmann R., Lode H. (1999). Toxicity of Quinolones. Drugs.

[196] Kim G.K. (2010). The Risk of Fluoroquinolone-Induced Tendinopathy and Tendon Rupture. J. Clin. Aesthet. Dermatol..

[197] Fernández-Cuadros M.E., Casique-Bocanegra L.O., Albaladejo-Florín M.J., Gómez-Dueñas S., Ramos-Gonzalez C., Pérez-Moro O.S. (2019). Bilateral Levofloxacin-Induced Achilles Tendon Rupture: An Uncommon Case Report and Review of the Literature. Clin. Med. Insights Arthritis Musculoskelet. Disord..

[198] Melhus A., Apelqvist J., Larsson J., Eneroth M. (2003). Levofloxacin-Associated Achilles Tendon Rupture and Tendinopathy. Scand. J. Infect. Dis..

[199] Gold L., Igra H. (2003). Levofloxacin-Induced Tendon Rupture: A Case Report and Review of the Literature. J. Am. Board Fam. Pract..

[200] Kowatari K., Nakashima K., Ono A., Yoshihara M., Amano M., Toh S. (2004). Levofloxacin-Induced Bilateral Achilles Tendon Rupture: A Case Report and Review of the Literature. J. Orthop. Sci..

[201] Baik S., Lau J., Huser V., McDonald C.J. (2020). Association between Tendon Ruptures and Use of Fluoroquinolone, and Other Oral Antibiotics: A 10-Year Retrospective Study of 1 Million US Senior Medicare Beneficiaries. BMJ Open.

[202] Childs S.G. (2007). Pathogenesis of Tendon Rupture Secondary to Fluoroquinolone Therapy. Orthop. Nurs..

[203] Williams R.J., Attia E., Wickiewicz T.L., Hannafin J.A. (2000). The Effect of Ciprofloxacin on Tendon, Paratenon, and Capsular Fibroblast Metabolism. Am. J. Sports Med..

[204] Shiu J., Ting G., Kiang T.K. (2019). Clinical Pharmacokinetics and Pharmacodynamics of Delafloxacin. Eur. J. Drug Metab. Pharmacokinet..

[205] Bassetti M., Puente F.D., Magnasco L., Giacobbe D.R. (2020). Innovative Therapies for Acute Bacterial Skin and Skin-Structure Infections (ABSSSI) Caused by Methicillin-Resistant Staphylococcus Aureus: Advances in Phase I and II Trials. Expert Opin. Investig. Drugs.

[206] O’Riordan W., McManus A., Teras J., Poromanski I., Cruz-Saldariagga M., Quintas M., Lawrence L., Liang S., Cammarata S. (2018). PROCEED Study Group A Comparison of the Efficacy and Safety of Intravenous Followed by Oral Delafloxacin with Vancomycin Plus Aztreonam for the Treatment of Acute Bacterial Skin and Skin Structure Infections: A Phase 3, Multinational, Double-Blind, Randomized Study. Clin. Infect. Dis..

[207] Pullman J., Gardovskis J., Farley B., Sun E., Quintas M., Lawrence L., Ling R., Cammarata S. (2017). PROCEED Study Group Efficacy and Safety of Delafloxacin Compared with Vancomycin plus Aztreonam for Acute Bacterial Skin and Skin Structure Infections: A Phase 3, Double-Blind, Randomized Study. J. Antimicrob. Chemother..

[208] Horcajada J.P., Salata R.A., Álvarez-Sala R., Nitu F.M., Lawrence L., Quintas M., Cheng C.-Y., Cammarata S. (2020). DEFINE-CABP Study Group A Phase 3 Study to Compare Delafloxacin With Moxifloxacin for the Treatment of Adults With Community-Acquired Bacterial Pneumonia (DEFINE-CABP). Open Forum. Infect. Dis..

[209] Blair K., Czyz C.N. (2022). Retinal Detachment. StatPearls.

[210] Marchant J. (2018). When Antibiotics Turn Toxic. Nature.

[211] Etminan M., Forooghian F., Brophy J.M., Bird S.T., Maberley D. (2012). Oral Fluoroquinolones and the Risk of Retinal Detachment. JAMA.

[212] Pasternak B., Svanström H., Melbye M., Hviid A. (2013). Association Between Oral Fluoroquinolone Use and Retinal Detachment. JAMA.

[213] Brett A.S. (2013). Oral Fluoroquinolone Use and Retinal Detachment: Reconciling Conflicting Findings in Observational Research. JAMA.

[214] Eftekhari K., Ghodasra D.H., Haynes K., Chen J., Kempen J.H., VanderBeek B.L. (2014). Risk of Retinal Tear or Detachment with Oral Fluoroquinolone Use: A Cohort Study. Pharmacoepidemiol. Drug Saf..

[215] Chui C.S.L., Wong I.C.K., Wong L.Y.L., Chan E.W. (2015). Association between Oral Fluoroquinolone Use and the Development of Retinal Detachment: A Systematic Review and Meta-Analysis of Observational Studies. J. Antimicrob. Chemother..

[216] Raguideau F., Dray-Spira R., Zureik M. (2016). Oral Fluoroquinolone Use and Retinal Detachment-Reply. JAMA Ophthalmol..

[217] Douglas I.J., Root A., Krishnan B. (2016). Oral Fluoroquinolone Use and Retinal Detachment. JAMA Ophthalmol..

[218] Baek Y.-H., Park S.J., Jeong S., Oh I.-S., Jeong H.E., Park K.H., Shin J.-Y. (2018). Signal Detection Between Fluoroquinolone Use and the Risk of Rhegmatogenous Retinal Detachment: Sequence Symmetry Analysis Using Nationwide South Korean Healthcare Database Between 2004 and 2015. Clin. Drug Investig..

[219] Shin J.-Y., Jeong S., Jeon H.-L., Byun S., Park K.H., Jeong H.E., Park S.J. (2018). The Risk Profile of Rhegmatogenous Retinal Detachment before and after Using a Fluoroquinolone: A 12 Year Nationwide Self-Controlled Case Series Study. J. Antimicrob. Chemother..

[220] Taher M.K., Habsah M., Bjerre L.M., Momoli F., Mattison D., Krewski D. (2021). Systemic Quinolones and Risk of Retinal Detachment II: Systematic Review of Clinical Trials. Clin. Med. Rev. Case Rep..

[221] Taher M.K., Alami A., Gravel C.A., Tsui D., Bjerre L.M., Momoli F., Mattison D., Krewski D. (2022). Systemic Quinolones and Risk of Retinal Detachment I: Analysis of Data from the US FDA Adverse Event Reporting System. Expert Opin. Drug Saf..

[222] Taher M.K., Crispo J.A.G., Fortin Y., Moog R., McNair D., Bjerre L.M., Momoli F., Mattison D., Krewski D. (2022). Systemic Quinolones and Risk of Retinal Detachment III: A Nested Case-Control Study Using a US Electronic Health Records Database. Eur. J. Clin. Pharmacol..

[223] Ponsioen T.L., van Luyn M.J.A., van der Worp R.J., van Meurs J.C., Hooymans J.M.M., Los L.I. (2008). Collagen Distribution in the Human Vitreoretinal Interface. Investig. Ophthalmol. Vis. Sci..

[224] Reviglio V.E., Hakim M.A., Song J.K., O’Brien T.P. (2003). Effect of Topical Fluoroquinolones on the Expression of Matrix Metalloproteinases in the Cornea. BMC Ophthalmol..

[225] Sharma C., Velpandian T., Baskar Singh S., Ranjan Biswas N., Bihari Vajpayee R., Ghose S. (2011). Effect of Fluoroquinolones on the Expression of Matrix Metalloproteinase in Debrided Cornea of Rats. Toxicol. Mech. Methods.

[226] Khaliq Y., Zhanel G.G. (2003). Fluoroquinolone-Associated Tendinopathy: A Critical Review of the Literature. Clin. Infect. Dis..

[227] Granowitz E.V., Brown R.B. (2008). Antibiotic Adverse Reactions and Drug Interactions. Crit. Care Clin..

[228] Hedenmalm K., Spigset O. (1996). Peripheral Sensory Disturbances Related to Treatment with Fluoroquinolones. J. Antimicrob. Chemother..

[229] Cohen J.S. (2001). Peripheral Neuropathy Associated with Fluoroquinolones. Ann. Pharmacother..

[230] Morales D., Pacurariu A., Slattery J., Pinheiro L., McGettigan P., Kurz X. (2019). Association Between Peripheral Neuropathy and Exposure to Oral Fluoroquinolone or Amoxicillin-Clavulanate Therapy. JAMA Neurol..

[231] Popescu C. (2018). Severe Acute Axonal Neuropathy Induced by Ciprofloxacin: A Case Report. CRN.

[232] Francis J.K., Higgins E. (2014). Permanent Peripheral Neuropathy: A Case Report on a Rare but Serious Debilitating Side-Effect of Fluoroquinolone Administration. J. Investig. Med. High Impact Case Rep..

[233] Estofan L.J.F., Naydin S., Gliebus G. (2018). Quinolone-Induced Painful Peripheral Neuropathy: A Case Report and Literature Review. J. Investig. Med. High Impact Case Rep..

[234] Scavone C., Mascolo A., Ruggiero R., Sportiello L., Rafaniello C., Berrino L., Capuano A. (2020). Quinolones-Induced Musculoskeletal, Neurological, and Psychiatric ADRs: A Pharmacovigilance Study Based on Data from the Italian Spontaneous Reporting System. Front. Pharm..

[235] Althaqafi A., Ali M., Alzahrani Y., Ming L.C., Hussain Z. (2021). How Safe Are Fluoroquinolones for Diabetic Patients? A Systematic Review of Dysglycemic and Neuropathic Effects of Fluoroquinolones. Ther. Clin. Risk Manag..

[236] Menarini Group A Randomized, Observer-Blinded, Active-Controlled, Phase Illb Study to Compare IV/Oral Delafloxacin Fixed-Dose Monotherapy With Best Available Treatments in a Microbiologically Enriched Population With Surgical Site Infections; clinicaltrials.gov, 2022. https://clinicaltrials.gov/ct2/show/NCT04042077.

[237] Miki M., Mikasa K., Kadota J., Mukae H., Fujita J., Hori S., Yanagihara K., Tateda K., Totsuka K., Umemoto Y. (2021). Phase III Double-Blind Comparative Study of Lascufloxacin versus Levofloxacin in Patients with Community-Acquired Pneumonia|Cochrane Library. Jpn. J. Chemother..

[238] Chung D.T., Tsai C.-Y., Chen S.-J., Chang L.-W., King C.-H.R., Hsu C.-H., Chiu K.-M., Tan H.-C., Chang Y.-T., Hsu M.-C. (2010). Multiple-Dose Safety, Tolerability, and Pharmacokinetics of Oral Nemonoxacin (TG-873870) in Healthy Volunteers. Antimicrob. Agents Chemother..

[239] van Rensburg D.J.J., Perng R.-P., Mitha I.H., Bester A.J., Kasumba J., Wu R.-G., Ho M.-L., Chang L.-W., Chung D.T., Chang Y.-T. (2010). Efficacy and Safety of Nemonoxacin versus Levofloxacin for Community-Acquired Pneumonia. Antimicrob. Agents Chemother..

[240] Liu Y., Zhang Y., Wu J., Zhu D., Sun S., Zhao L., Wang X., Liu H., Ren Z., Wang C. (2017). A Randomized, Double-Blind, Multicenter Phase II Study Comparing the Efficacy and Safety of Oral Nemonoxacin with Oral Levofloxacin in the Treatment of Community-Acquired Pneumonia. J. Microbiol. Immunol. Infect..

[241] Fujita J., Cash H.L., Niki Y., Kadota J., Yanagihara K., Kohno S., Kaku M., Watanabe A., Aoki N., Hori S. (2013). Clinical and Bacteriological Efficacies of Sitafloxacin against Community-Acquired Pneumonia Caused by Streptococcus Pneumoniae: Nested Cohort within a Multicenter Clinical Trial. J. Infect. Chemother..

[242] Han H., Kim S.E., Shin K.-H., Lim C., Lim K.S., Yu K.-S., Cho J.-Y. (2013). Comparison of Pharmacokinetics between New Quinolone Antibiotics: The Zabofloxacin Hydrochloride Capsule and the Zabofloxacin Aspartate Tablet. Curr. Med. Res. Opin..

[243] Rhee C.K., Chang J.H., Choi E.G., Kim H.K., Kwon Y.-S., Kyung S.Y., Lee J.-H., Park M.J., Yoo K.H., Oh Y.M. (2015). Zabofloxacin versus Moxifloxacin in Patients with COPD Exacerbation: A Multicenter, Double-Blind, Double-Dummy, Randomized, Controlled, Phase III, Non-Inferiority Trial. Int. J. Chron. Obs. Pulmon. Dis..

[244] Tomé A.M., Filipe A. (2011). Quinolones: Review of Psychiatric and Neurological Adverse Reactions. Drug Saf..

[245] Kaur K., Fayad R., Saxena A., Frizzell N., Chanda A., Das S., Chatterjee S., Hegde S., Baliga M.S., Ponemone V. (2016). Fluoroquinolone-Related Neuropsychiatric and Mitochondrial Toxicity: A Collaborative Investigation by Scientists and Members of a Social Network. J. Community Support Oncol..

[246] Samyde J., Petit P., Hillaire-Buys D., Faillie J.-L. (2016). Quinolone Antibiotics and Suicidal Behavior: Analysis of the World Health Organization’s Adverse Drug Reactions Database and Discussion of Potential Mechanisms. Psychopharmacology.

[247] Sellick J., Mergenhagen K., Morris L., Feuz L., Horey A., Risbood V., Wojciechowski A., Ruh C., Bednarczyk E., Conway E. (2018). Fluoroquinolone-Related Neuropsychiatric Events in Hospitalized Veterans. Psychosomatics.

[248] Kushner J.M., Peckman H.J., Snyder C.R. (2001). Seizures Associated with Fluoroquinolones. Ann. Pharmacother..

[249] Freeman M.Z., Cannizzaro D.N., Naughton L.F., Bove C. (2021). Fluoroquinolones-Associated Disability: It Is Not All in Your Head. NeuroSci.

[250] Owens R.C., Ambrose P.G. (2000). Clinical Use of the Fluoroquinolones. Med. Clin. N. Am..

[251] Akahane K., Sekiguchi M., Une T., Osada Y. (1989). Structure-Epileptogenicity Relationship of Quinolones with Special Reference to Their Interaction with Gamma-Aminobutyric Acid Receptor Sites. Antimicrob. Agents Chemother..

[252] O’Riordan W., Mehra P., Manos P., Kingsley J., Lawrence L., Cammarata S. (2015). A Randomized Phase 2 Study Comparing Two Doses of Delafloxacin with Tigecycline in Adults with Complicated Skin and Skin-Structure Infections. Int. J. Infect. Dis..

[253] Jorgensen S.C.J., Mercuro N.J., Davis S.L., Rybak M.J. (2018). Delafloxacin: Place in Therapy and Review of Microbiologic, Clinical and Pharmacologic Properties. Infect. Dis. Ther..

[254] Jjingo C.J. Clinical Review (Baxdela) 2016. https://www.accessdata.fda.gov/drugsatfda_docs/nda/2017/208610Orig1s000,208611Orig1s000MedR.pdf.

[255] Maddix D.S., Stefani A. (1992). Comment: Myasthenia Gravis and Ciprofloxacin. Ann. Pharmacother..

[256] Sieb J.P., Milone M., Engel A.G. (1996). Effects of the Quinoline Derivatives Quinine, Quinidine, and Chloroquine on Neuromuscular Transmission. Brain Res..

[257] Tintinalli J.E. (2004). Fluoroquinolones Should Be Avoided in Myasthenia Gravis. Ann. Emerg. Med..

[258] Gunduz A., Turedi S., Kalkan A., Nuhoglu I. (2006). Levofloxacin Induced Myasthenia Crisis. Emerg Med. J..

[259] Pham Nguyen T.P., Leonard C.E., Bird S.J., Willis A.W., Hamedani A.G. (2021). Pharmacosafety of Fluoroquinolone and Macrolide Antibiotics in the Clinical Care of Patients with Myasthenia Gravis. Muscle Nerve.

[260] Deshpande S.S., Sheridan R.E., Adler M. (1997). Efficacy of Certain Quinolines as Pharmacological Antagonists in Botulinum Neurotoxin Poisoning. Toxicon.

[261] Sieb J.P. (1998). Fluoroquinolone Antibiotics Block Neuromuscular Transmission. Neurology.

[262] van der Linden P.D., van der Lei J., Vlug A.E., Stricker B.H.C. (1998). Skin Reactions to Antibacterial Agents in General Practice. J. Clin. Epidemiol..

[263] Balakirski G., Merk H.F. (2017). Cutaneous Allergic Drug Reactions: Update on Pathophysiology, Diagnostic Procedures and Differential Diagnosic. Cutan. Ocul. Toxicol..

[264] Sable D., Murakawa G.J. (2003). Quinolones in Dermatology. Clin. Dermatol..

[265] Scherer K., Bircher A.J. (2005). Hypersensitivity Reactions to Fluoroquinolones. Curr. Allergy Asthma Rep..

[266] Tang W., Rao E. (2022). Anaphylaxis to Ciprofloxacin Requiring Emergent Surgical Cricothyrotomy. Eur. J. Case Rep. Intern. Med..

[267] EMA Factive: Withdrawn Application. https://www.ema.europa.eu/en/medicines/human/withdrawn-applications/factive.

[268] Menarini International Operations Luxembourg S.A. Withdrawal Letter Factive. https://www.ema.europa.eu/en/documents/other/withdrawal-letter-factive_en.pdf.

[269] Yilmaz İ., Doğan S., Tutar N., Kanbay A., Büyükoğlan H., Demir R. (2012). Biphasic Anaphylaxis to Gemifloxacin. Asia Pac. Allergy.

[270] McGee E.U., Samuel E., Boronea B., Dillard N., Milby M.N., Lewis S.J. (2019). Quinolone Allergy. Pharmacy.

[271] Renaudin J.-M., Beaudouin E., Ponvert C., Demoly P., Moneret-Vautrin D.-A. (2013). Severe Drug-Induced Anaphylaxis: Analysis of 333 Cases Recorded by the Allergy Vigilance Network from 2002 to 2010. Allergy.

[272] Manfredi M., Severino M., Testi S., Macchia D., Ermini G., Pichler W.J., Campi P. (2004). Detection of Specific IgE to Quinolones. J. Allergy Clin. Immunol..

[273] Kulthanan K., Chularojanamontri L., Manapajon A., Dhana N., Jongjarearnprasert K. (2011). Cutaneous Adverse Reactions to Fluoroquinolones. Dermatitis.

[274] Devadarshini S., Pattnaik K.P., Samal R., Mohapatra J., Sahoo S.S. (2017). Comparative Analysis of Cutaneous Drug Reactions among Different Fluoroquinolones: An Experimental Study. Int. J. Basic Clin. Pharmacol..

[275] Neuman M.G., Cohen L.B., Nanau R.M. (2015). Quinolones-Induced Hypersensitivity Reactions. Clin. Biochem..

[276] Li R., Bernstein J.A. (2016). Anaphylactic Shock Caused By Moxifloxacin without Cross-Reactivity to Other Fluoroquinolones. J. Allergy Clin. Immunol..

[277] Doña I., Moreno E., Pérez-Sánchez N., Andreu I., Hernández Fernandez de Rojas D., Torres M.J. (2017). Update on Quinolone Allergy. Curr. Allergy Asthma Rep..

[278] Portilho N.C., Aun M.V., Kalil J., Giavina-Bianchi P. (2020). Quinolone-Induced Anaphylaxis. Curr. Treat. Options Allergy.

[279] Fernández T.D., Ariza A., Palomares F., Montañez M.I., Salas M., Martín-Serrano A., Fernández R., Ruiz A., Blanca M., Mayorga C. (2016). Hypersensitivity to Fluoroquinolones. Medicine.

[280] Doña I., Pérez-Sánchez N., Salas M., Barrionuevo E., Ruiz-San Francisco A., Hernández Fernández de Rojas D., Martí-Garrido J., Andreu-Ros I., López-Salgueiro R., Moreno E. (2020). Clinical Characterization and Diagnostic Approaches for Patients Reporting Hypersensitivity Reactions to Quinolones. J. Allergy Clin. Immunol. Pract..

[281] Doña I., Blanca-López N., Boteanu C., Cueva-Oliver B., Fernández-Sánchez F., Gajate P., García-Avilés M., García-Núñez I., Lobera T., Moreno E. (2021). Clinical Practice Guidelines for Diagnosis and Management of Hypersensitivity Reactions to Quinolones. J. Investig. Allergol. Clin. Immunol..

[282] Liu R., Hu S., Zhang Y., Che D., Cao J., Wang J., Zhao T., Jia Q., Wang N., Zhang T. (2019). Mast Cell-Mediated Hypersensitivity to Fluoroquinolone Is MRGPRX2 Dependent. Int. Immunopharmacol..

[283] Rodvold K.A., Gotfried M.H., Chugh R., Gupta M., Yeole R., Patel A., Bhatia A. (2018). Intrapulmonary Pharmacokinetics of Levonadifloxacin Following Oral Administration of Alalevonadifloxacin to Healthy Adult Subjects. Antimicrob. Agents Chemother..

[284] Lin L., Chang L.-W., Tsai C.-Y., Hsu C.-H., Chung D.T., Aronstein W.S., Ajayi F., Kuzmak B., Lyon R.A. (2010). Dose Escalation Study of the Safety, Tolerability, and Pharmacokinetics of Nemonoxacin (TG-873870), a Novel Potent Broad-Spectrum Nonfluorinated Quinolone, in Healthy Volunteers. Antimicrob. Agents Chemother..

[285] Guo B., Wu X., Zhang Y., Shi Y., Yu J., Cao G., Zhang J. (2012). Safety and Clinical Pharmacokinetics of Nemonoxacin, a Novel Non-Fluorinated Quinolone, in Healthy Chinese Volunteers following Single and Multiple Oral Doses. Clin. Drug Investig..

[286] Wu X., Zhang J., Guo B., Zhang Y., Yu J., Cao G., Chen Y., Zhu D., Ye X., Wu J. (2015). Pharmacokinetics and Pharmacodynamics of Multiple-Dose Intravenous Nemonoxacin in Healthy Chinese Volunteers. Antimicrob. Agents Chemother..

[287] Sousa J., Alves G., Fortuna A., Falcão A. (2014). Third and Fourth Generation Fluoroquinolone Antibacterials: A Systematic Review of Safety and Toxicity Profiles. Curr. Drug Saf..

[288] Singh A.P., Kumar V., Singh N. (2012). Phototoxic Potential Assessment of Enoxacin In Vitro: Phototoxicity.

[289] Kawada A., Hatanaka K., Gomi H., Matsuo I. (1999). In Vitro Phototoxicity of New Quinolones: Production of Active Oxygen Species and Photosensitized Lipid Peroxidation. Photodermatol. Photoimmunol. Photomed..

[290] Jałbrzykowska K., Chrzanowska A., Roszkowski P., Struga M. (2022). The New Face of a Well-Known Antibiotic: A Review of the Anticancer Activity of Enoxacin and Its Derivatives. Cancers.

[291] Mang R., Stege H., Krutmann J., Johansen J.D., Frosch P.J., Lepoittevin J.-P. (2011). Mechanisms of Phototoxic and Photoallergic Reactions. Contact Dermatitis.

[292] de Guidi G., Bracchitta G., Catalfo A. (2011). Photosensitization Reactions of Fluoroquinolones and Their Biological Consequences. Photochem. Photobiol..

[293] Robertson D.G., Epling G.A., Kiely J.S., Bailey D.L., Song B. (1991). Mechanistic Studies of the Phototoxic Potential of PD 117596, a Quinolone Antibacterial Compound. Toxicol. Appl. Pharmacol..

[294] Moore D.E. (1998). Mechanisms of Photosensitization by Phototoxic Drugs. Mutat. Res. Fundam. Mol. Mech. Mutagen..

[295] Martínez L., Chignell C.F. (1998). Photocleavage of DNA by the Fluoroquinolone Antibacterials. J. Photochem. Photobiol. B Biol..

[296] Matsumoto M., Kojima K., Nagano H., Matsubara S., Yokota T. (1992). Photostability and Biological Activity of Fluoroquinolones Substituted at the 8 Position after UV Irradiation. Antimicrob. Agents Chemother..

[297] Thompson A.M. (2007). Ocular Toxicity of Fluoroquinolones. Clin. Exp. Ophthalmol..

[298] Marrot L., Agapakis-Causse C. (2000). Differences in the Photogenotoxic Potential of Two Fluoroquinolones as Shown in Diploid Yeast Strain (Saccharomyces Cerevisae) and Supercoiled Plasmid DNA. Mutat. Res..

[299] Fasani E., Mella M., Caccia D., Tassi S., Fagnoni M., Albini A. (1997). The Photochemistry of Lomefloxacin. An Aromatic Carbene as the Keyintermediate in Photodecomposition. Chem. Commun..

[300] Cuquerella M.C., Miranda M.A., Bosca F. (2006). Generation of Detectable Singlet Aryl Cations by Photodehalogenation of Fluoroquinolones. J. Phys. Chem. B.

[301] Kowalska J., Banach K., Rok J., Beberok A., Rzepka Z., Wrześniok D. (2020). Molecular and Biochemical Basis of Fluoroquinolones-Induced Phototoxicity—The Study of Antioxidant System in Human Melanocytes Exposed to UV-A Radiation. Int. J. Mol. Sci..

[302] Viola G., Facciolo L., Canton M., Vedaldi D., Dall’Acqua F., Aloisi G.G., Amelia M., Barbafina A., Elisei F., Latterini L. (2004). Photophysical and Phototoxic Properties of the Antibacterial Fluoroquinolones Levofloxacin and Moxifloxacin. Chem. Biodivers..

[303] Dawe R.S., Ferguson J., Ibbotson S., Lawrence L., Paulson S., Duffy E., Cammarata S. (2018). Lack of Phototoxicity Potential with Delafloxacin in Healthy Male and Female Subjects: Comparison to Lomefloxacin. Photochem. Photobiol. Sci..

[304] Nandanwar M., Kansagara A., Gupta S., Patel A., Patel M.A., Yeole R., Thorve D., Patel M. (2022). Preclinical Safety Evaluation of Levonadifloxacin, a Novel Anti-Methicillin-Resistant Staphyloccocus Aureus Benzoquinolizine Fluoroquinolone by Intravenous and Oral Administration. J. Appl. Toxicol..

[305] Chugh R., Lakdavala F., Bhatia A. (2016). Safety and Pharmacokinetics of Multiple Ascending Doses of WCK 771 and WCK 2349. Proceedings of the Abstract P1268.

[306] Bhawsar S., Kale R., Deshpande P., Yeole R., Bhagwat S., Patel M. (2021). Design and Synthesis of an Oral Prodrug Alalevonadifloxacin for the Treatment of MRSA Infection. Bioorganic Med. Chem. Lett..

[307] Dawe R.S., Ibbotson S.H., Sanderson J.B., Thomson E.M., Ferguson J. (2003). A Randomized Controlled Trial (Volunteer Study) of Sitafloxacin, Enoxacin, Levofloxacin and Sparfloxacin Phototoxicity. Br. J. Dermatol..

[308] Bech-Thomsen N., Angelo H.R., Wulf H.C. (1994). Skin Pigmentation as a Predictor of Minimal Phototoxic Dose after Oral Methoxsalen. Arch. Dermatol..

[309] Lojanapiwat B., Nimitvilai S., Bamroongya M., Jirajariyavej S., Tiradechavat C., Malithong A., Predanon C., Tanphaichitra D., Lertsupphakul B. (2019). Oral Sitafloxacin vs Intravenous Ceftriaxone Followed by Oral Cefdinir for Acute Pyelonephritis and Complicated Urinary Tract Infection: A Randomized Controlled Trial. Infect. Drug Resist..

[310] Nelson R.L., Suda K.J., Evans C.T. (2017). Antibiotic Treatment for Clostridium Difficile-associated Diarrhoea in Adults. Cochrane Database Syst. Rev..

[311] Dhalla I.A., Mamdani M.M., Simor A.E., Kopp A., Rochon P.A., Juurlink D.N. (2006). Are Broad-Spectrum Fluoroquinolones More Likely To Cause Clostridium Difficile-Associated Disease?. Antimicrob. Agents Chemother..

[312] Weiss K. (2009). Clostridium Difficile and Fluoroquinolones: Is There a Link?. Int. J. Antimicrob. Agents.

[313] McCusker M.E., Harris A.D., Perencevich E., Roghmann M.-C. (2003). Fluoroquinolone Use and Clostridium Difficile–Associated Diarrhea. Emerg. Infect. Dis..

[314] Deshpande A., Pant C., Jain A., Fraser T.G., Rolston D.D.K. (2008). Do Fluoroquinolones Predispose Patients to Clostridium Difficile Associated Disease? A Review of the Evidence. Curr. Med. Res. Opin..

[315] Johnson S. (2009). Recurrent Clostridium Difficile Infection: A Review of Risk Factors, Treatments, and Outcomes. J. Infect..

[316] Eze P., Balsells E., Kyaw M.H., Nair H. (2017). Risk Factors for Clostridium Difficile Infections—An Overview of the Evidence Base and Challenges in Data Synthesis. J. Glob. Health.

[317] Davies K., Lawrence J., Berry C., Davis G., Yu H., Cai B., Gonzalez E., Prantner I., Kurcz A., Macovei I. (2020). Risk Factors for Primary Clostridium Difficile Infection; Results from the Observational Study of Risk Factors for Clostridium Difficile Infection in Hospitalized Patients With Infective Diarrhea (ORCHID). Front. Public Health.

[318] Zaiss N.H., Witte W., Nübel U. (2010). Fluoroquinolone Resistance and Clostridium Difficile, Germany. Emerg. Infect. Dis..

[319] Donskey C.J. (2017). Fluoroquinolone Restriction to Control Fluoroquinolone-Resistant Clostridium Difficile. Lancet Infect. Dis..

[320] Kyorin Pharmacetical Lasvic Tablets 75mg. https://www.rad-ar.or.jp/siori/english/search/result?n=42565.

[321] Mehta K.D., Sharma J.B., Anand A., Reddy N P.K., Kadam P., Debnath K., Bhapkar S., Thampi B.M. (2022). Real-World Evidence of Efficacy and Safety of Levonadifloxacin (Oral and IV) in the Management of Acute Bacterial Skin and Skin Structure Infections (ABSSSI): Findings of a Retrospective, Multi-Center Study. Cureus.

[322] Recanatini M., Poluzzi E., Masetti M., Cavalli A., De Ponti F. (2005). QT Prolongation through HERG K+ Channel Blockade: Current Knowledge and Strategies for the Early Prediction during Drug Development. Med. Res. Rev..

[323] Teng C., Walter E.A., Gaspar D.K.S., Obodozie-Ofoegbu O.O., Frei C.R. (2019). Torsades de Pointes and QT Prolongation Associations with Antibiotics: A Pharmacovigilance Study of the FDA Adverse Event Reporting System. Int. J. Med. Sci..

[324] Kang J., Wang L., Chen X.-L., Triggle D.J., Rampe D. (2001). Interactions of a Series of Fluoroquinolone Antibacterial Drugs with the Human Cardiac K+ Channel HERG. Mol. Pharmacol..

[325] Owens R.C. (2004). QT Prolongation with Antimicrobial Agents. Drugs.

[326] Briasoulis A., Agarwal V., Pierce W.J. (2011). QT Prolongation and Torsade de Pointes Induced by Fluoroquinolones: Infrequent Side Effects from Commonly Used Medications. CRD.

[327] Rubinstein E., Camm J. (2002). Cardiotoxicity of Fluoroquinolones. J. Antimicrob. Chemother..

[328] Roden D.M. (2008). Long-QT Syndrome. N. Engl. J. Med..

[329] Khan F., Ismail M., Khan Q., Ali Z. (2018). Moxifloxacin-Induced QT Interval Prolongation and Torsades de Pointes: A Narrative Review. Expert Opin. Drug Saf..

[330] Owens R.C. (2001). Risk Assessment for Antimicrobial Agent-Induced QTc Interval Prolongation and Torsades de Pointes. Pharmacother. J. Hum. Pharmacol. Drug Ther..

[331] Prabhakar M., Krahn A.D. (2004). Ciprofloxacin-Induced Acquired Long QT Syndrome. Heart Rhythm..

[332] Keivanidou A., Arnaoutoglou C., Krommydas A., Papanikolaou G., Tsiptses K., Chrisopoulos C., Kirpizidis C. (2009). Ciprofloxacin Induced Acquired Long QT Syndrome in a Patient under Class III Antiarrhythmic Therapy. Cardiol. J..

[333] Noel G.J., Natarajan J., Chien S., Hunt T.L., Goodman D.B., Abels R. (2003). Effects of Three Fluoroquinolones on QT Interval in Healthy Adults after Single Doses. Clin. Pharmacol. Ther..

[334] Tsikouris J.P., Peeters M.J., Cox C.D., Meyerrose G.E., Seifert C.F. (2006). Effects of Three Fluoroquinolones on QT Analysis after Standard Treatment Courses. Ann. Noninvasive Electrocardiol..

[335] Stancampiano F.F., Palmer W.C., Getz T.W., Serra-Valentin N.A., Sears S.P., Seeger K.M., Pagan R.J., Racho R.G., Ray J.C., Snipelisky D.F. (2015). Rare Incidence of Ventricular Tachycardia and Torsades de Pointes in Hospitalized Patients with Prolonged QT Who Later Received Levofloxacin: A Retrospective Study. Mayo Clin. Proc..

[336] Badshah A., Janjua M., Younas F., Halabi A.R., Cotant J.F. (2009). Moxifloxacin-Induced QT Prolongation and Torsades: An Uncommon Effect of a Common Drug. Am. J. Med. Sci..

[337] Matsukura S., Nakamura Y., Hoshiai K., Hayashi T., Koga T., Goto A., Chiba K., Lubna N.J., Hagiwara-Nagasawa M., Izumi-Nakaseko H. (2018). Effects of Moxifloxacin on the Proarrhythmic Surrogate Markers in Healthy Filipino Subjects: Exposure-Response Modeling Using ECG Data of Thorough QT/QTc Study. J. Pharmacol. Sci..

[338] Täubel J., Prasad K., Rosano G., Ferber G., Wibberley H., Cole S.T., Van Langenhoven L., Fernandes S., Djumanov D., Sugiyama A. (2020). Effects of the Fluoroquinolones Moxifloxacin and Levofloxacin on the QT Subintervals: Sex Differences in Ventricular Repolarization. J. Clin. Pharmacol..

[339] Taubel J., Ferber G., Lorch U., Batchvarov V., Savelieva I., Camm A.J. (2014). Thorough QT Study of the Effect of Oral Moxifloxacin on QTc Interval in the Fed and Fasted State in Healthy Japanese and Caucasian Subjects. Br. J. Clin. Pharmacol..

[340] Litwin J.S., Benedict M.S., Thorn M.D., Lawrence L.E., Cammarata S.K., Sun E. (2015). A Thorough QT Study to Evaluate the Effects of Therapeutic and Supratherapeutic Doses of Delafloxacin on Cardiac Repolarization. Antimicrob. Agents Chemother..

[341] Mason J.W., Chugh R., Patel A., Gutte R., Bhatia A. (2019). Electrocardiographic Effects of a Supratherapeutic Dose of WCK 2349, a Benzoquinolizine Fluoroquinolone. Clin. Transl. Sci..

[342] Zhang Y., Dai X., Wang T., Chen X., Liang L., Qiao H., Tsai C., Chang L., Huang P., Hsu C. (2014). Effects of an Al3+- and Mg2+-Containing Antacid, Ferrous Sulfate, and Calcium Carbonate on the Absorption of Nemonoxacin (TG-873870) in Healthy Chinese Volunteers. Acta Pharmacol. Sin..

[343] Zhang Y., Dai X., Yang Y., Chen X., Wang T., Tang Y., Tsai C., Chang L., Chang Y., Zhong D. (2016). Effects of Probenecid and Cimetidine on the Pharmacokinetics of Nemonoxacin in Healthy Chinese Volunteers. Drug Des. Devel. Ther..

[344] Zhao C., Lv Y., Li X., Hou F., Ma X., Wei M., Kang Z., Cui L., Xia Y., Liu Y. (2018). Effects of Nemonoxacin on thorough ECG QT/QTc Interval: A Randomized, Placebo- and Positive-Controlled Crossover Study in Healthy Chinese Adults. Clin. Ther..

[345] Kang Y., Li Y., Xu F., Zhang J., Wang K., Chen Y., Wu J., Guo B., Yu J., Zhang Y. (2019). Population Pharmacokinetics Study of Nemonoxacin Among Chinese Patients with Moderate Hepatic Impairment. Clin. Ther..

[346] Kelesidis T., Canseco E. (2010). Quinolone-Induced Hypoglycemia: A Life-Threatening but Potentially Reversible Side Effect. Am. J. Med..

[347] Ellis D.E., Hubbard R.A., Willis A.W., Zuppa A.F., Zaoutis T.E., Hennessy S. (2022). Comparative Risk of Serious Hypoglycemia among Persons Dispensed a Fluoroquinolone versus a Non-Fluoroquinolone Antibiotic. Diabetes Res. Clin. Pract..

[348] El Ghandour S., Azar S.T. (2015). Dysglycemia Associated with Quinolones. Prim. Care Diabetes.

[349] Kelesidis T., Canseco E. (2009). Levofloxacin-Induced Hypoglycemia: A Rare but Life-Threatening Side Effect of a Widely Used Antibiotic. Am. J. Med..

[350] Park-Wyllie L.Y., Juurlink D.N., Kopp A., Shah B.R., Stukel T.A., Stumpo C., Dresser L., Low D.E., Mamdani M.M. (2006). Outpatient Gatifloxacin Therapy and Dysglycemia in Older Adults. N. Engl. J. Med..

[351] Yip C., Lee A.J. (2006). Gatifloxacin-Induced Hyperglycemia: A Case Report and Summary of the Current Literature. Clin. Ther..

[352] Bobba R.K., Arsura E.L. (2006). Hyperglycemia in an Elderly Diabetic Patient: Drug-Drug or Drug-Disease Interaction?. South. Med. J..

[353] Murad M.H., Coto-Yglesias F., Wang A.T., Sheidaee N., Mullan R.J., Elamin M.B., Erwin P.J., Montori V.M. (2009). Drug-Induced Hypoglycemia: A Systematic Review. J. Clin. Endocrinol. Metab..

[354] Ishiwata Y., Sanada Y., Yasuhara M. (2006). Effects of Gatifloxacin on Serum Glucose Concentration in Normal and Diabetic Rats. Biol. Pharm. Bull..

[355] Kingsley J., Mehra P., Lawrence L.E., Henry E., Duffy E., Cammarata S.K., Pullman J. (2016). A Randomized, Double-Blind, Phase 2 Study to Evaluate Subjective and Objective Outcomes in Patients with Acute Bacterial Skin and Skin Structure Infections Treated with Delafloxacin, Linezolid or Vancomycin. J. Antimicrob. Chemother..

[356] Totsuka K., Sesoko S., Fukase H., Ikushima I., Odajima M., Niwayama Y. (2020). Pharmacokinetic Study of Lascufloxacin in Non-Elderly Healthy Men and Elderly Men. J. Infect. Chemother..

[357] Furuie H., Tanioka S., Shimizu K., Manita S., Nishimura M., Yoshida H. (2018). Intrapulmonary Pharmacokinetics of Lascufloxacin in Healthy Adult Volunteers. Antimicrob. Agents Chemother..

[358] (2020). NIDDK Fluoroquinolones. LiverTox: Clinical and Research Information on Drug-Induced Liver Injury.

[359] Giustarini G., Huppelschoten S., Barra M., Oppelt A., Wagenaar L., Weaver R.J., Bol-Schoenmakers M., Smit J.J., van de Water B., Klingmüller U. (2020). The Hepatotoxic Fluoroquinolone Trovafloxacin Disturbs TNF- and LPS-Induced P65 Nuclear Translocation in Vivo and in Vitro. Toxicol. Appl. Pharmacol..

[360] Leitner J.M., Graninger W., Thalhammer F. (2010). Hepatotoxicity of Antibacterials: Pathomechanisms and Clinical Data. Infection.

[361] Andrade R.J., Tulkens P.M. (2011). Hepatic Safety of Antibiotics Used in Primary Care. J. Antimicrob. Chemother..

[362] Hirsch A.C., Lundquist L.M. (2009). Ciprofloxacin-Induced Hepatotoxicity Resolved with Levofloxacin: A Case Report and a Review of the Literature. Hosp. Pharm..

[363] Orman E.S., Conjeevaram H.S., Vuppalanchi R., Freston J.W., Rochon J., Kleiner D.E., Hayashi P.H. (2011). Clinical and Histopathologic Features of Fluoroquinolone-Induced Liver Injury. Clin. Gastroenterol. Hepatol..

[364] Alshammari T.M., Larrat E.P., Morrill H.J., Caffrey A.R., Quilliam B.J., Laplante K.L. (2014). Risk of Hepatotoxicity Associated with Fluoroquinolones: A National Case–Control Safety Study. Am. J. Health Syst. Pharm..

[365] Taher M.K., Alami A., Gravel C.A., Tsui D., Bjerre L.M., Momoli F., Mattison D.R., Krewski D. (2021). Systemic Quinolones and Risk of Acute Liver Failure I: Analysis of Data from the US FDA Adverse Event Reporting System. JGH Open.

[366] Sun Q., Zhu R., Foss F.W., Macdonald T.L. (2007). Mechanisms of Trovafloxacin Hepatotoxicity: Studies of a Model Cyclopropylamine-Containing System. Bioorg. Med. Chem. Lett..

[367] Hoover R., Hunt T., Benedict M., Paulson S.K., Lawrence L., Cammarata S., Sun E. (2016). Single and Multiple Ascending-Dose Studies of Oral Delafloxacin: Effects of Food, Sex, and Age. Clin. Ther..

[368] Temple R. (2006). Hy’s Law: Predicting Serious Hepatotoxicity. Pharmacoepidemiol. Drug Saf..

[369] Yuan J., Mo B., Ma Z., Lv Y., Cheng S.-L., Yang Y., Tong Z., Wu R., Sun S., Cao Z. (2019). Safety and Efficacy of Oral Nemonoxacin versus Levofloxacin in Treatment of Community-Acquired Pneumonia: A Phase 3, Multicenter, Randomized, Double-Blind, Double-Dummy, Active-Controlled, Non-Inferiority Trial. J. Microbiol. Immunol. Infect..

[370] Kohno S., Niki Y., Kadota J.-I., Yanagihara K., Kaku M., Watanabe A., Aoki N., Hori S., Fujita J., Tanigawara Y. (2013). Clinical Dose Findings of Sitafloxacin Treatment: Pharmacokinetic-Pharmacodynamic Analysis of Two Clinical Trial Results for Community-Acquired Respiratory Tract Infections. J. Infect. Chemother..

[371] Hartmann A., Alder A.C., Koller T., Widmer R.M. (1998). Identification of Fluoroquinolone Antibiotics as the Main Source of UmuC Genotoxicity in Native Hospital Wastewater. Environ. Toxicol. Chem..

[372] Ikbal M., Doğan H., Odabaş H., Pirim İ. (2004). Genotoxic Evaluation of the Antibacterial Drug, Ciprofloxacin, in Cultured Lymphocytes of Patients with Urinary Tract Infection. Turk. J. Med. Sci..

[373] Hu J., Wang W., Zhu Z., Chang H., Pan F., Lin B. (2007). Quantitative Structure−Activity Relationship Model for Prediction of Genotoxic Potential for Quinolone Antibacterials. Environ. Sci. Technol..

[374] Khadra A., Pinelli E., Lacroix M.Z., Bousquet-Melou A., Hamdi H., Merlina G., Guiresse M., Hafidi M. (2012). Assessment of the Genotoxicity of Quinolone and Fluoroquinolones Contaminated Soil with the Vicia Faba Micronucleus Test. Ecotoxicol. Environ. Saf..

[375] Zhu Q., Li T., Wei X., Li J., Wang W. (2014). In Silico and in Vitro Genotoxicity Evaluation of Descarboxyl Levofloxacin, an Impurity in Levofloxacin. Drug Chem. Toxicol..

[376] Du M., Zhang D., Hou Y., Zhao X., Li Y. (2019). Combined 2D-QSAR, Principal Component Analysis and Sensitivity Analysis Studies on Fluoroquinolones’ Genotoxicity. Int. J. Environ. Res. Public Health.

[377] Zhao X., Wang X., Li Y. (2019). Combined HQSAR Method and Molecular Docking Study on Genotoxicity Mechanism of Quinolones with Higher Genotoxicity. Environ. Sci. Pollut. Res..

[378] PubChem Amifloxacin. https://pubchem.ncbi.nlm.nih.gov/compound/55492.

[379] Arjona A. (2009). Nemonoxacin Quinolone Antibiotic. Drug Future.

[380] Qin X., Huang H. (2014). Review of Nemonoxacin with Special Focus on Clinical Development. Drug Des. Devel. Ther..

[381] Li M., Wei D., Zhao H., Du Y. (2014). Genotoxicity of Quinolones: Substituents Contribution and Transformation Products QSAR Evaluation Using 2D and 3D Models. Chemosphere.

[382] Bhattacharya P., Mukherjee S., Mandal S.M. (2020). Fluoroquinolone Antibiotics Show Genotoxic Effect through DNA-Binding and Oxidative Damage. Spectrochim. Acta Part A Mol. Biomol. Spectrosc..

[383] Fengxian C., Reti H. (2017). Analysis of Positions and Substituents on Genotoxicity of Fluoroquinolones with Quantitative Structure-Activity Relationship and 3D Pharmacophore Model. Ecotoxicol. Environ. Saf..

[384] Kishii R., Yamaguchi Y., Takei M. (2017). In Vitro Activities and Spectrum of the Novel Fluoroquinolone Lascufloxacin (KRP-AM1977). Antimicrob Agents Chemother.

[385] (2020). Taigexyn^®^ Nemonoxacin Capsule 250 Mg.

[386] Shiomi M., Togawa M., Fujita K., Murata R. (1999). Effect of Early Oral Fluoroquinolones in Hemorrhagic Colitis Due to Escherichia Coli O157:H7. Pediatr. Int..

[387] Safdar N., Said A., Gangnon R.E., Maki D.G. (2002). Risk of Hemolytic Uremic Syndrome after Antibiotic Treatment of Escherichia Coli O157:H7 Enteritis: A Meta-Analysis. JAMA.

[388] Panos G.Z., Betsi G.I., Falagas M.E. (2006). Systematic Review: Are Antibiotics Detrimental or Beneficial for the Treatment of Patients with Escherichia Coli O157:H7 Infection?. Aliment. Pharmacol. Ther..

[389] Ohnishi K., Nakamura-Uchiyama F. (2012). Does Levofloxacin Induce Hemolytic Uremic Syndrome in Patients Infected with Verotoxin-Producing Escherichia Coli O157 Infections?. Jpn. J. Infect. Dis..

[390] Geerdes-Fenge H.F., Löbermann M., Nürnberg M., Fritzsche C., Koball S., Henschel J., Höhn R., Schober H.C., Mitzner S., Podbielski A. (2013). Ciprofloxacin Reduces the Risk of Hemolytic Uremic Syndrome in Patients with Escherichia Coli O104:H4-Associated Diarrhea. Infection.

[391] Kakoullis L., Papachristodoulou E., Chra P., Panos G. (2019). Shiga Toxin-Induced Haemolytic Uraemic Syndrome and the Role of Antibiotics: A Global Overview. J. Infect..

[392] Mody R.K., Hoekstra R.M., Scott M.K., Dunn J., Smith K., Tobin-D’Angelo M., Shiferaw B., Wymore K., Clogher P., Palmer A. (2021). Risk of Hemolytic Uremic Syndrome Related to Treatment of Escherichia Coli O157 Infection with Different Antimicrobial Classes. Microorganisms.

[393] Maguire R.B., Stroncek D.F., Gale E., Yearlsey M. (1994). Hemolytic Anemia and Acute Renal Failure Associated with Temafloxacin-Dependent Antibodies. Am. J. Hematol..

[394] Moise P.A., Birmingham M.C., Schentag J.J. (2000). Pharmacokinetics and Metabolism of Moxifloxacin. Drugs Today.

[395] Mulgaonkar A., Venitz J., Sweet D.H. (2012). Fluoroquinolone Disposition: Identification of the Contribution of Renal Secretory and Reabsorptive Drug Transporters. Expert Opin. Drug Metab. Toxicol..

[396] Lomaestro B.M. (2000). Fluoroquinolone-Induced Renal Failure. Drug Saf..

[397] Stahlmann R., Lode H. (2010). Safety Considerations of Fluoroquinolones in the Elderly. Drugs Aging.

[398] Bird S.T., Etminan M., Brophy J.M., Hartzema A.G., Delaney J.A.C. (2013). Risk of Acute Kidney Injury Associated with the Use of Fluoroquinolones. CMAJ.

[399] Farid S., Mahmood M., Abu Saleh O.M., Hamadah A., Nasr S.H., Garrigos Z.E., Leung N., Sohail M.R. (2018). Clinical Manifestations and Outcomes of Fluoroquinolone-Related Acute Interstitial Nephritis. Mayo Clin. Proc..

[400] Stratta P., Lazzarich E., Canavese C., Bozzola C., Monga G. (2007). Ciprofloxacin Crystal Nephropathy. Am. J. Kidney Dis..

[401] Khan M., Ortega L.M., Bagwan N., Nayer A. (2015). Crystal-Induced Acute Kidney Injury Due to Ciprofloxacin. J. Nephropathol..

[402] Perazella M.A., Shirali A., Gilbert S.J., Weiner D.E. (2014). 37—Kidney Disease Caused by Therapeutic Agents. National Kidney Foundation Primer on Kidney Diseases.

[403] Jaman M., Chowdhury A.A., Rana A.A., Masum S.M., Ferdous T., Rashid M.A., Karim M.M. (2015). In Vitro Evaluation of Ciprofloxacin Hydrochloride. Bangladesh J. Sci. Ind. Res..

[404] Torniainen K., Tammilehto S., Ulvi V. (1996). The Effect of PH, Buffer Type and Drug Concentration on the Photodegradation of Ciprofloxacin. Int. J. Pharm..

[405] Roca Jalil M.E., Baschini M., Sapag K. (2015). Influence of PH and Antibiotic Solubility on the Removal of Ciprofloxacin from Aqueous Media Using Montmorillonite. Appl. Clay Sci..

[406] Hoover R.K., Alcorn H., Lawrence L., Paulson S.K., Quintas M., Cammarata S.K. (2018). Delafloxacin Pharmacokinetics in Subjects With Varying Degrees of Renal Function. J. Clin. Pharmacol..

[407] Hoover R., Hunt T., Benedict M., Paulson S.K., Lawrence L., Cammarata S., Sun E. (2016). Safety, Tolerability, and Pharmacokinetic Properties of Intravenous Delafloxacin After Single and Multiple Doses in Healthy Volunteers. Clin. Ther..

[408] Hoover R., Alcorn H., Lawrence L., Paulson S.K., Quintas M., Cammarata S.K. (2018). Pharmacokinetics of Intravenous Delafloxacin in Patients With End-Stage Renal Disease. J. Clin. Pharmacol..

[409] Pierfitte C., Royer R.J., Moore N., Bégaud B. (2000). The Link between Sunshine and Phototoxicity of Sparfloxacin. Br. J. Clin. Pharmacol..

[410] Trisciuoglio D., Krasnowska E., Maggi A., Pozzi R., Parasassi T., Sapora O. (2002). Phototoxic Effect of Fluoroquinolones on Two Human Cell Lines. Toxicol Vitr..

[411] Fasano C.J., O’Malley G., Dominici P., Aguilera E., Latta D.R. (2008). Comparison of Octreotide and Standard Therapy versus Standard Therapy Alone for the Treatment of Sulfonylurea-Induced Hypoglycemia. Ann. Emerg. Med..

[412] McLaughlin S.A., Crandall C.S., McKinney P.E. (2000). Octreotide: An Antidote for Sulfonylurea-Induced Hypoglycemia. Ann. Emerg. Med..

[413] Hassan Z., Wright J. (2007). Use of Octreotide Acetate to Prevent Rebound Hypoglycaemia in Sulfonylurea Overdose. Emerg. Med. J..

[414] Muanda F.T., Sood M.M., Weir M.A., Sontrop J.M., Ahmadi F., Yoo E., Kim R.B., Silverman M.S., Knoll G.A., Garg A.X. (2022). Association of Higher-Dose Fluoroquinolone Therapy with Serious Adverse Events in Older Adults With Advanced Chronic Kidney Disease. JAMA Netw. Open.

[415] de With K., Allerberger F., Amann S., Apfalter P., Brodt H.-R., Eckmanns T., Fellhauer M., Geiss H.K., Janata O., Krause R. (2016). Strategies to Enhance Rational Use of Antibiotics in Hospital: A Guideline by the German Society for Infectious Diseases. Infection.

[416] Sartelli M., Weber D.G., Ruppé E., Bassetti M., Wright B.J., Ansaloni L., Catena F., Coccolini F., Abu-Zidan F.M., Coimbra R. (2016). Antimicrobials: A Global Alliance for Optimizing Their Rational Use in Intra-Abdominal Infections (AGORA). World J. Emerg. Surg..

[417] Adikwu E., Deo O. (2012). Fluoroquinolones Reported Hepatotoxicity. Pharmacol. Pharm..

[418] Yan K., Zhu M., Jia Y., Wang J., Cai Y. (2022). Efficacy and Safety of Quinolones vs. Other Antimicrobials for the Treatment of Uncomplicated Urinary Tract Infections in Adults: A Systematic Review and Meta-Analysis. Int. Urogynecol. J..

[419] Haiping L., Ziqiang J., Qina Z., Yuhua D. (2019). Adverse Reactions of Fluoroquinolones to Central Nervous System and Rational Drug Use in Nursing Care. Pak. J. Pharm. Sci..

