# Supplementary material for: Overview of Side-Effects of Antibacterial Fluoroquinolones: New Drugs versus Old Drugs, a Step Forward in the Safety Profile?
Source: Pharmaceutics. 2023 Mar 1;15(3):804. doi: 10.3390/pharmaceutics15030804 (PMC10056716; doi:10.3390/pharmaceutics15030804)
Supplement: Supplementary file 1 [file pharmaceutics-15-00804-s001.zip › pharmaceutics-2092231-supplementary.pdf]

pharmaceutics

MDPI

Review—Supplementary Material

Overview of Side-Effects of Antibacterial Fluoroquinolones: New Drugs versus Old Drugs, a Step Forward in the Safety Profile?

Aura Rusu [1,*], Alexandra-Cristina Munteanu [2], Eliza-Mihaela Arbănași [1] and Valentina Uivarosi [2]

1 Pharmaceutical and Therapeutical Chemistry Department, Faculty of Pharmacy, George Emil Palade University of Medicine, Pharmacy, Science, and Technology of Targu Mures, 540142 Targu Mures, Romania; arbanasi.eliza-mihaela@stud18.umfst.ro
2 Department of General and Inorganic Chemistry, Faculty of Pharmacy, Carol Davila University of Medicine and Pharmacy, 6 Traian Vuia Str., 020956 Bucharest, Romania; alexandra.ticea@umfcd.ro (A.-C.M.); valentina.uivarosi@umfcd.ro (V.U.)
* Correspondence: aura.rusu@umfst.ro; Tel.: +40-766-600-898

Citation: Rusu, A.; Munteanu, A.-C.; Arbănași, E.-M.; Uivarosi, V. Overview of Side-Effects of Antibacterial Fluoroquinolones: New Drugs versus Old Drugs, a Step Forward in the Safety Profile? *Pharmaceutics* **2023**, *15*, 804. https://doi.org/10.3390/pharmaceutics15030804

Academic Editor: Clive Prestidge

Received: 27 November 2022
Revised: 7 February 2023
Accepted: 17 February 2023
Published: 1 March 2023

Copyright: © 2023 by the authors. Licensee MDPI, Basel, Switzerland. This article is an open access article distributed under the terms and conditions of the Creative Commons Attribution (CC BY) license (https://creativecommons.org/licenses/by/4.0/).

Table S1. Notable AEs of novel FQs (AEs – adverse effects, ALT – alanine aminotransferase, AST – aspartate aminotransferase, b.i.d. – two times a day, COPD – Chronic Obstructive Pulmonary Disease, FQs – fluoroquinolones, i.v. – intravenous, qd – once a day, TEAE – treatment-emergent adverse event, Ref. – References, UTI – Urinary tract infections).

FQs	Type of clinical study	Adverse effects (AEs)	Ref.
**Delafloxacin (RX-3341, ABT-492, WQ-3034)**	Pooled phase 3 safety data for delafloxacin (300 mg i.v. in a 1-hour infusion every 12 h) versus vancomycin/aztreonam (vancomycin: 15 mg/kg every 12 h or local standard of care, with concomitant aztreonam, multiple-dose: 5–14 d) in AB-SSSI.	TEAEs in the delafloxacin group versus the comparator group were seen in 22.1% and 26.1% of patients, respectively. TEAEs: nausea 6.1% versus 4.3%, diarrhoea 6.1% and 2.0% for delafloxacin and vancomycin/aztreonam, respectively. Gastrointestinal events were reported at comparable rates when delafloxacin was given by i.v. (17.3%) or by i.v. followed by oral doses (16.8%). Skin and subcutaneous tissue disorders were seen at 0.9% for delafloxacin and 4.7% for vancomycin/aztreonam. Most TEAEs were mild or moderate in severity, with < 4% of patients in either group experiencing severe TEAEs. 1.5% of patients treated with delafloxacin withdrew from the study due to an adverse effect compared with 2.8% of patients treated with vancomycin/aztreonam.	[51], [207], [206],
	Randomized, double-blind, comparator-controlled, multicenter, global phase 3 study compared the efficacy and safety of delafloxacin 300 mg twice daily or moxifloxacin 400 mg once daily in adults with CABP.	65 (15.2%) in the delafloxacin group and 54 (12.6%) AEs in the moxifloxacin group were considered at least possibly related to the study drug. Most were mild in severity. Nineteen subjects (4.4%) in the delafloxacin group and 14 (3.3%) in the moxifloxacin group experienced severe TEAEs. Two (0.5%) in the delafloxacin group (hypersensitivity and *C. difficile* colitis) and none in the moxifloxacin group had SAEs that were considered potentially related to the study drug. Diarrhoea, increased transaminases, and headache were the only TEAEs reported in ≥ 2% of subjects.	[208]
	Phase 1, open-label pharmacokinetic and safety study of a single i.v. dose of 300 mg delafloxacin in subjects with mild, moderate, and severe hepatic impairment (Child-Pugh class A, B, and C, respectively) compared with matched healthy controls.	11 TEAEs were reported, and 10 of 39 subjects (26%) reported at least 1 TEAE after receiving 300 mg i.v. delafloxacin. Adverse events included infections (n = 3: hordeolum, tinea, nasopharyngitis), drug hypersensitivity (n = 2), headache and presyncope (n = 2), eye pain, abdominal pain, musculoskeletal pain, infusion site pain (n = 1 each). All TEAEs were mild to moderate in severity and resolved by the end of the study. There were no deaths or serious adverse events reported.	[367]
	Randomized clinical study of delafloxacin powder for solution for infusion 300 mg or tablet 450 mg, BID, for 5 to 14 days compared to best available therapy in patients with surgical site infections.	*Clostridium difficile* colitis 1/134 (0.75%); Diarrhea 3/134 (2.24%); Nausea 2/134 (1.49%); Hypokalaemia 3/134 (2.24%); White blood cell count increased 2/134 (1.49%); Headache 2/134 (1.49%); Pruritus 2/134 (1.49%); Phlebitis 2/134 (1.49%).	[236]
	Open-label, parallel-group crossover study in subjects with normal renal function or mild, moderate, or severe renal impairment (300 mg delafloxacin i.v., placebo i.v., and 400 mg	Most commonly reported TEAEs - gastrointestinal disorders (6 of 34 subjects [17.6%]), diarrhoea most commonly reported TEAE (4 subjects). No severe TEAEs occurred during the study.	[406]

FQs	Type of clinical study	Adverse effects (AEs)	Ref.
	delafloxacin orally in 3 periods separated by ≥14-day washouts).		
	1) A randomized, double-blind, placebo-controlled, single-(300, 450, 600, 750, 900, and 1200 mg) ascending-dose study of i.v. delafloxacin in 62 (52 active, 10 placebo) healthy volunteers; 2) A randomized, double-blind, placebo-controlled study of i.v. delafloxacin (300 mg) given as a single dose on day 1, followed by twice-daily dosing on days 2 through 14, in 12 (8 active, 4 placebo) healthy volunteers; 3) An open-label, randomized, 2-period, 2-sequence crossover study in which 56 healthy volunteers were randomly assigned to 1 of 2 sequences of a single oral dose of delafloxacin (450 mg) or i.v. delafloxacin (300 mg).	A total of 16 participants (32%) reported a TEAE, most of which were classified as gastrointestinal disorders (primarily nausea, vomiting, and diarrhoea) and CNS disorders. Most AEs were classified as mild and considered possibly related to delafloxacin. One participant treated with delafloxacin discontinued participation in the study because of an adverse effect of nausea and vomiting considered moderate in severity.	[407]
	Open-label, parallel-group, crossover study (300 mg delafloxacin containing sulfobutylether-β-cyclodextrin in 2 periods separated by ≥14-day washouts)	Delafloxacin was well tolerated in healthy subjects and subjects with end-stage renal disease. In ESRD subjects on hemodialysis, 27 TEAEs were reported, with 10 of 10 subjects (100%) experiencing at least 1 TEAE after receiving i.v. delafloxacin immediately before or after hemodialysis. The most commonly reported TEAEs overall were classified as gastrointestinal disorders (5 of 10 subjects [50%]), with diarrhoea, nausea, and vomiting reported for 2 of 10 subjects each.	[408]
	Phase 1, investigator-blind, placebo/active-controlled, randomized, parallel-group study conducted in 52 healthy male and female volunteers who received 200 or 400 mg of oral delafloxacin, 400 mg oral lomefloxacin or placebo, once daily for six days.	No phototoxicity was reported for delafloxacin, but lomefloxacin demonstrated moderate phototoxicity. Five subjects in the delafloxacin 400 mg and 1 subject in the placebo group reported diarrhoea, considered to be probably or possibly related to the study drug. AEs were mild or moderate in intensity and resolved spontaneously.	[303]
	Randomized, phase 2, double-blind, multicenter trial. Patients were randomized 1:1:1 to receive delafloxacin 300 mg i.v. every 12 h, delafloxacin 450 mg i.v. every 12 h, or tigecycline	Most frequent AEs: nausea, vomiting, and diarrhoea. 11 delafloxacin-treated patients (two in the 300 mg arm and nine in the 450 mg arm) and one tigecycline-treated patient had below-normal serum glucose values after having had normal values at baseline. Serious TEAE: a generalized seizure in a 53-year-old male in the delafloxacin 450 mg arm. Both delafloxacin groups had	[252]

FQs	Type of clinical study	Adverse effects (AEs)	Ref.
	100 mg i.v. × 1, followed by 50 mg i.v. every 12 h.	a lower incidence of gastrointestinal side-effects than the tigecycline group, but the 300 mg delafloxacin arm was the best-tolerated regimen.	
	Double-blind, phase 2 trial, 256 patients were randomized (1:1:1) to 300 mg of delafloxacin, 600 mg of linezolid or 15 mg/kg vancomycin (actual body weight), each administered i.v. twice daily for 5–14 days.	The most frequently reported TEAEs were nausea, diarrhoea and vomiting. Five patients (two in the delafloxacin and three in the vancomycin group) experienced a total of six TEAEs related to liver toxicity. TEAE of hyperglycaemia occurred in two, one and two patients in the delafloxacin, linezolid and vancomycin groups, respectively. No patient had a TEAE related to hypoglycaemia. No reports of *Clostridium difficile* diarrhoea. Reported SAEs of convulsions occurred in patients with a history of seizure disorder. No clinically relevant changes in the QT interval or clinically significant electrocardiogram abnormalities.	[355]
	Randomized, double-blind, placebo-controlled, 4-period crossover study was conducted in 52 healthy adults to assess the effect of delafloxacin on the corrected QT (QTc) interval after dosing with delafloxacin at 300 mg i.v. (therapeutic), delafloxacin at 900 mg i.v. (supratherapeutic), moxifloxacin at 400 mg orally (oral; positive control), and placebo.	Gastrointestinal TEAEs – vomiting, nausea - (23 subjects [44.2%]) were the most frequently experienced events, followed by nervous system disorders – headache, dizziness- (14 patients [26.9%]) and general disorders and administration site conditions (13 patients [25.0%]). No deaths, serious AEs, or AEs leading to study discontinuation and no clinically significant abnormalities in laboratory values or vital signs were observed at any time point after any dose of the study drug.	[340]
	2 phase 2 studies:<br>1) Single and multiple ascending-dose study that included evaluations of the effects of food, sex, and age; 2) Single-dose, randomized, 3-period crossover study -participants received 900 mg delafloxacin under fasted conditions, with a high-fat meal, or fasted with a high-fat meal 2 hours after dosing.	Diarrhoea - most common TEAE reported – 3 of the participants at the 1200-mg once-daily dose had diarrhoea of moderate intensity; all other incidents of diarrhoea were of mild intensity. Other TEAEs: rash, increased Alt, AST values. No evidence of significant QTc interval prolongation was observed.	[367]
	Open-label, noninferiority, phase 3, multicenter, randomized study in which 460 participants with uncomplicated gonorrhoea at 25 study centres were randomized (2:1) to receive a single 900 mg oral dose of delafloxacin or 250 mg intramuscular ceftriaxone.	Most common AEs: diarrhoea (delafloxacin: 31.9%, ceftriaxone: 7.1%), nausea (delafloxacin: 7.9%, ceftriaxone: 1.3%), headache (delafloxacin: 4.9%, ceftriaxone: 4.5%), vomiting (delafloxacin: 2.6%, ceftriaxone: 0.6%). Three participants in the delafloxacin group discontinued the study because of TEAEs of vomiting within 30 minutes after receiving the study drug.	[84]

FQs	Type of clinical study	Adverse effects (AEs)	Ref.
**Lascufloxacin (AM-197)**	Phase 3, double-blind, randomized, comparative study of lascufloxacin versus levofloxacin in patients with CAP - subjects were administered lascufloxacin 150 mg (300 mg on the initial day) by iv infusion once daily, or lascufloxacin 500 mg by i.v. infusion once daily. Both drugs were administered for a period of 7 to 14 days.	The incidence of AEs was 17.9% (25/140) in the lascufloxacin group and 19.0% (26/137) in the levofloxacin group. No deaths or severe safety problems were reported.	[237]
	Phase 3, double-blind, comparative study of lascufloxacin 75 mg once daily versus levofloxacin 500 mg once daily in patients with acute sinusitis or acute exacerbation of chronic sinusitis.	TEAEs: diarrhoea (lascufloxacin: 0.7%, lascufloxacin: 0%), blood insulin increased (lascufloxacin: 0.7%, levofloxacin: 0.7%), eosinophil count increased (lascufloxacin: 2.1%, levofloxacin: 0.7%), blood gamma-glutamyltransferase increased (lascufloxacin: 0.7%, levofloxacin: 0%), white blood cell count decreased (lascufloxacin: 0.7%, levofloxacin: 0%), blood alkaline phosphatase increased (lascufloxacin: 0.7%, levofloxacin: 0%), asthma (lascufloxacin: 0.7%, levofloxacin: 0%), rash (lascufloxacin: 0%, levofloxacin: 0.7%), headache (lascufloxacin: 0%, levofloxacin: 1.4%)	[85]
	Phase 1 clinical study to examine the pharmacokinetic profiles and safety of lascufloxacin multiple-dose oral administration of 75 mg (tablets) or single-dose oral administration of lascufloxacin 100-800 mg (capsules) in non-elderly Japanese healthy men and the effects of ageing on lascufloxacin 200 mg (capsules) pharmacokinetics in elderly Japanese healthy men.	AEs for which the causal relationship could not be denied was found only in 1 subject (1 case) receiving lascufloxacin 200mg (blood creatinine increased) and 2 subjects (2 cases) receiving lascufloxacin 800 mg (blood creatinine increased and white blood cell count decreased, 1 case each)	[356]
**Levonadifloxacin (WCK 771) and Alalevonadifloxacin (WCK 2349)**	Intrapulmonary pharmacokinetics study following oral administration of alalevonadifloxacin (1,000 mg twice daily for five days) to 30 healthy adult subjects.	The most frequent TEAEs included photophobia in four subjects and dysgeusia in four subjects. Other AEs related to the study drug were leukopenia, back pain, headache, and skin papule. No clinically significant changes were observed in physical examination findings, vital signs, or ECGs. All TEAEs were mild in severity, and no serious adverse events were reported.	[283]
	Crossover-designed thorough QT study in 48 healthy subjects randomized to treatment with placebo, oral alalevonadifloxacin 2,600 mg, or oral moxifloxacin, 400 mg	Alalevonadifloxacin exerted no significant effect on baseline-and placebo-corrected QTcF, QRS, or PR interval.	[341]
	A post-marketing, multi-centric, retrospective, observational study (PIONEER study)	Only two AEs were reported out of 227 subjects. One patient on i.v. therapy experienced diarrhoea, while an-	[321]

FQs	Type of clinical study	Adverse effects (AEs)	Ref.
	in 227 patients with ABSSSI receiving levonadifloxacin (oral and/or i.v.)	other on oral medication accused vomiting. Both incidents were mild and were quickly resolved. No severe AEs were reported.	
	Phase 3, open-label, randomized, active-comparator study in 500 subjects. Oral levonadifloxacin 1000 mg was compared with oral linezolid 600 mg, and i.v. levonadifloxacin 800 mg was compared with i.v. linezolid 600 mg, twice daily for 7–10 days.	Incidences of TEAEs were similar between treatment groups and between i.v. (20.8% vs. 22.4%, for levonadifloxacin and linezolid, respectively) and oral therapy (16.0% vs 13.5% for levonadifloxacin and linezolid, respectively). Gastrointestinal disorders (5.2% vs 6.0% for pooled levonadifloxacin and linezolid, respectively) were the most frequently reported AEs. The most common AEs in the levonadifloxacin-group were constipation (3.6%) (with mild severity), increased blood glucose (1.6%) (with mild to moderate severity and not related to levonadifloxacin; the majority of these patients had high blood glucose at screening), and cough (1.2%) (with mild severity and not related to levonadifloxacin). Four subjects were discontinued from the study due to TEAEs (two in the i.v. levonadifloxacin group and two in the i.v. linezolid group). Five serious TEAEs were reported in five subjects in the i.v. groups; three of these subjects died during the study period (one in i.v. levonadifloxacin and two in the i.v. linezolid group). All serious TEAEs were considered not related to the study drugs.	[41]
**Nemonoxacin (TG-873870)**	Randomized, double-blind, multicenter phase 2 study comparing the efficacy and safety of oral nemonoxacin with oral levofloxacin in the treatment of CAP.	The most common drug-related treatment-emergent adverse events (TEAEs) (≥2%) were nausea, vomiting, leucopenia, and abnormal liver function. Drug-related QT interval prolongation.	[240]
	Double-blind, ascending-single-dose study.	The most frequent adverse events were contact dermatitis, pruritus, and erythema. Twelve subjects out of 56 who were administered nemonoxacin experienced a total of 20 AEs, including 5 cases of mild ECG electrode contact dermatitis (12%), 5 cases of moderate pruritus (12%), 4 cases of mild erythema (10%), 2 cases of mild headache (5%), and 1 case each of mild oral hypoesthesia, abdominal pain, rash, and nausea (2%).	[284]
	Two single-dose, open-label, randomized, crossover studies were conducted in 24 healthy male Chinese volunteers (12 per study).	The AE profile did not differ between volunteers who received nemonoxacin only or those who received nemonoxacin and metal-ion-containing drugs. The most commonly reported AEs were increased blood uric acid and WBC count. For Study 1, a total of 13 AEs were reported by 7 volunteers. Two episodes of creatine phosphokinase increased (1 following administration of nemonoxacin only, 1 following administration of nemonoxacin 2 h before antacid) reported by the same subject were considered possibly related to the study drug. For Study 2, a total of 17 AEs were reported by 6	[312]

FQs	Type of clinical study	Adverse effects (AEs)	Ref.
		volunteers. Six episodes of blood uric acid increased reported by 3 volunteers (2 following administration of nemonoxacin only, 1 following administration of nemonoxacin with ferrous sulfate, and 1 following administration of nemonoxacin with calcium carbonate) and 1 episode of increased ALT reported by 1 volunteer following the administration of nemonoxacin only were considered possibly related to study drug.	
	A randomized, placebo-and positive-controlled crossover study in healthy Chinese adults.	Face flushing, pruritus (for subjects treated with nemonoxacin 750 mg in fasted condition), skin rash (for subjects treated with nemonoxacin 500 mg in fed condition).	[344]
	Two independent, open-label, randomized, crossover studies were conducted on 24 (12 per study) healthy Chinese volunteers.	In Study 1, one episode of sinus bradycardia and one episode of prolonged QT on ECG were considered to be possibly related to the study drug. In Study 2, one episode of increased blood uric acid, one episode of upper abdominal pain, and two episodes of flatulence were considered to be possibly related to the study drug. All the AEs in both studies were mild and resolved without treatment. None of the volunteers withdrew from the study because of an AE.	[343]
	Randomized, double-blind, multicenter study.	Nemonoxacin 750 mg: neutropenia (9.3%), dizziness (3.5%), nausea (5.8%), thrombocythemia (4.7%), headache (1.2%), diarrhoea (1.2%), ECG QTc interval prolonged (2.3%), blood amylase increased (1.2%); Nemonoxacin 750 mg: neutropenia (9.0%), dizziness (4.5%), nausea (1.1%), thrombocythemia (2.2%), headache (2.2%), diarrhoea (5.6%), blood amylase increased (1.1%).	[239]
	Three multicenter, randomized, double-blind, parallel, comparative clinical trials were conducted in Taiwan, South Africa, and China.	Gastrointestinal, hematologic, and hepatic disorders - most common drug-related AEs. Nemonoxacin 750 mg group had a higher frequency of nausea, neutropenia, leukopenia, thrombocytosis, prolonged QT interval, and abnormal liver function. The incidence of increased ALT was higher in the Nemonoxacin 500 mg group. The prevalence of common (≥1%) drug-related AEs was comparable between the treatment groups. CNS disorders, such as dizziness and headache (Nemonoxacin 500 mg: 1.9% and 1%; Nemonoxacin 750 mg: 1.9% and 1.3%) - lower than those reported for frequently used QNs.	[30]
	Multiple-dose safety, tolerability, and pharmacokinetics of oral nemonoxacin (TG-873870) in healthy volunteers.	AEs headache, contact dermatitis, and rash were mild and resolved spontaneously.	[238]
	Multiple-dose i.v. nemonoxacin in healthy Chinese volunteers.	Rash increased ALT and AST levels and elevation of the total bilirubin level.	[286]
	Population pharmacokinetics study of nemonoxacin among Chinese patients with moderate hepatic impairment.	Patients with moderate hepatic impairment: decreased white blood cell count (1 subject), decreased platelet count (2 subjects), increased total bilirubin level (2 subjects), increased ALT level (1 subject), increased AST	[345]

FQs	Type of clinical study	Adverse effects (AEs)	Ref.
		level (1 subject), mild T-wave change (1 subject), prolonged QT interval (2 subjects). Healthy subjects: headache (1 subject), ventricular premature beat (1 subject), T-wave changes (2 subjects), and sinus bradycardia (1 subject).	
	Safety and clinical pharmacokinetics of nemonoxacin in healthy Chinese volunteers following single and multiple oral doses.	Nausea and rash with or without pruritus were mild and resolved spontaneously; transaminase elevations.	[285]
	Phase 3, multicenter, randomized, double-blind, double-dummy, active-controlled, non-inferiority trial oral nemonoxacin versus levofloxacin in the treatment of CAP.	ALT elevation (nemonoxacin 5.1%), white blood cell decreased (nemonoxacin 2.0%), nausea (nemonoxacin 3.1%), vomiting (nemonoxacin 1.7%).	[369]
	Randomized, double-blind, placebo-controlled, dose-escalating safety and tolerability study in 92 subjects and a randomized, single-dose, open-label, 3-period<br>Latin-square crossover pharmacokinetic study in 12 subjects.	Local irritation at the injection site erythematous rash with or without pruritus.	[148]
**Sitafloxacin (DU-6859a)**	Pharmacokinetic-pharmacodynamic analysis of two clinical trial results for community-acquired respiratory tract infections.	The incidence of adverse events related to the study drug was 33.7% in the 100 mg qd group and 40.4% in the 50 mg bid group. The most common adverse drug reactions were diarrhoea (12.2% in the 100 mg qd group and 8.4% in the 50 mg bid group), increased levels of ALT (9.2% in the 100 mg qd group and 5.4% in the 50 mg bid group), increased levels of AST (8.2% in the 100 mg qd group and 9.6% in the 50 mg bid group), and increased eosinophil counts (3.1% in the 100-mg qd group and 7.2% in the 50 mg bid group).	[128], [370]
	Nested cohort within a multicenter clinical trial – sitafloxacin against CAP caused by *Streptococcus pneumoniae*	Minor AEs observed in 21 of the 48 patients (43.8 %) in the 100 mg once-daily group and in 5 of the 24 patients (20.8 %) in the 50 mg twice-daily group. The most common AEs were diarrhoea (drug-related 14.6% in the 100 mg once-daily group, 4.17% in the 50 mg twice-daily group), increased ALT (drug-related 14.6% in the 100 mg once-daily group, 8.33% in the 50 mg twice-daily group), increased AST (drug-related 14.6% in the 100 mg once-daily group, 8.33% in the 50 mg twice-daily group), and headache (drug-related 2.08% in the 100 mg once-daily group, 4.17% in the 50 mg twice-daily group). No severe AEs occurred in either group.	[241]
	Randomized controlled trial: Oral sitafloxacin vs intravenous ceftriaxone followed by oral	Drug-related AEs were more frequent in the sitafloxacin group (11.3%) than in the ceftriaxone/cefdinir group (5.2%). AEs that resulted in the discontinuation of study antibiotics were observed in two subjects (1.3%) in the	[309]

FQs	Type of clinical study	Adverse effects (AEs)	Ref.
	cefdinir for acute pyelonephritis and complicated UTI.	sitafloxacin group and one (0.6%) in the ceftriaxone/cefdinir group. No phototoxicity or severe AEs were observed in any study patients in either group.	
	Randomized controlled trial (volunteer study) of sitafloxacin, enoxacin, levofloxacin and sparfloxacin phototoxicity.	Caucasian subjects - mild UVA-dependent phototoxicity in the sitafloxacin group, as opposed to severe phototoxicity in the sparfloxacin group and moderate phototoxicity in the enoxacin group. Phototoxicity was not detected in the levofloxacin or placebo groups. In the Oriental study, no clinically relevant phototoxicity was seen with either sitafloxacin or placebo groups.	[307]
	Clinical study of sitafloxacin in febrile complicated pyelonephritis.	Most common AEs: increased ALT 18.2% (4/22), increased AST 9.1% (2/22), diarrhoea 13.6% (3/22), alkaline phosphatase 9.1% (2/22). All AEs were mild in severity.	[131]
	Clinical study of sitafloxacin in male nongonococcal urethritis.	Most common AE - diarrhoea 13.6% (6/44).	[132]
	Clinical study of sitafloxacin in the treatment of male gonococcal urethritis.	Most common AEs: diarrhoea 16.7% (2/12), headache 8.3% (1/12), and increased bilirubin 8.3% (1/12)	[133]
	Clinical study of sitafloxacin in treatment of cervicitis with *Chlamydia trachomatis.*	Most common AEs: diarrhoea 9.3% (4/43), vaginal candidiasis 7.0% (3/43), pruritus genital 2.3% (1/43), and increased eosinophil count 2.3% (1/43).	[134]
	Comparative study on sitafloxacin and levofloxacin in complicated UTI.	Most common AEs: diarrhoea (sitafloxacin: 8.2%, levofloxacin: 1.7%), abdominal pain (sitafloxacin: 2.5%, levofloxacin: 1.7%), increased ALT (sitafloxacin: 1.6%, levofloxacin: 0.8%), increased AST (sitafloxacin: 1.6%, levofloxacin: 0%), increased triglycerides (sitafloxacin: 1.6%, levofloxacin: 0.8%), increased eosinophil count (sitafloxacin: 1.6%, levofloxacin: 0.8%).	[135]
	Dose-comparative study of sitafloxacin in complicated UTI.	Group L - 50 mg b.i.d. oral sitafloxacin; Group H - 100 mg b.i.d. oral sitafloxacin. Most common AEs: diarrhoea (Group L: 6.9%, Group H: 10.3%), increased ALT (Group L: 2.3%, Group H: 0.8%), increased AST (Group L: 1.5%, Group H: 1.6%), rash (Group L: 1.5%, Group H: 1.6%), increased creatine phosphokinase (Group L: 2.3%, Group H: 0.8%), increased eosinophil count (Group L: 2.3%, Group H: 0.8%), and decreased blood glucose (Group L: 1.5%, Group H: 0%).	[136]
	Open study of sitafloxacin in patients with respiratory tract infections.	Group L - 50 mg b.i.d. oral sitafloxacin; Group H - 100 mg b.i.d. oral sitafloxacin. Most common AEs: diarrhoea (Group L: 10.4%, Group H: 24.2%), increased ALT (Group L: 4.3%, Group H: 9.1%), increased AST (Group L: 8.7%, Group H: 6.1%), increased eosinophil count (Group L: 8.7%, Group H: 3.0%), increased gamma-glutamyltransferase (Group L: 2.6%, Group H: 3.0%), and headache (Group L: 1.7%, Group H: 0%).	[137]
	Clinical pharmacology study using sitafloxacin at 50 mg per single dose and a phase 3 clinical study using sitafloxacin at	AEs which occurred in more than one patient: diarrhoea 22.4%, increased ALT 10.2%, increased AST 6.1%, and increased eosinophil count 4.1%	[138]

FQs	Type of clinical study	Adverse effects (AEs)	Ref.
	50 mg or 100 mg b.i.d. for 3 to 7 days		
	Phase 3 double-blind comparative study of sitafloxacin 100 mg orally twice daily versus tosufloxacin 150 mg three times daily in patients with CAP.	AEs which occurred in more than one patient: diarrhoea (sitafloxacin: 21.4%, tosufloxacin: 6.6%), increased ALT (sitafloxacin: 18.3%, tosufloxacin: 14.9%), increased AST (sitafloxacin: 10.3%, tosufloxacin: 5.8%), increased eosinophil count (sitafloxacin: 8.7%, tosufloxacin: 4.1%), increased gamma-glutamyltransferase (sitafloxacin: 6.3%, tosufloxacin: 5.0%), increased creatine phosphokinase (sitafloxacin: 2.4%, tosufloxacin: 0%), increased alkaline phosphatase (sitafloxacin: 3.2%, tosufloxacin: 2.5%), headache (sitafloxacin: 4%, tosufloxacin: 2.5%), upper abdominal pain (sitafloxacin: 2.4%, tosufloxacin: 0%), decreased blood glucose (sitafloxacin: 1.6%, tosufloxacin: 0.8%), increased lactate dehydrogenase (sitafloxacin: 1.6%, tosufloxacin: 1.7%), and nausea (sitafloxacin: 1.6%, tosufloxacin: 0%).	[139]
	Double-blind comparative study of sitafloxacin 50 mg orally twice daily versus levofloxacin 100 mg three times daily in patients with respiratory tract infection.	Adverse drug reactions which occurred in more than one patient: diarrhoea (sitafloxacin: 7.9%, levofloxacin: 4.3%), increased ALT (sitafloxacin: 7.0%, levofloxacin: 6.9%), increased AST (sitafloxacin: 5.3%, levofloxacin: 6.9%), increased eosinophil count (sitafloxacin: 4.4%, levofloxacin: 1.7%), nausea (sitafloxacin: 2.6%, levofloxacin: 0.9%), increased blood potassium levels (sitafloxacin: 1.8%, levofloxacin: 0%), and increased blood triglycerides (sitafloxacin: 1.8%, levofloxacin: 0.9%).	[140]
**Zabofloxacin (hydrochloride: DW224a), (aspartate: DW-224aa)**	Randomized, open-label, single-dose, two-way crossover study comparing zabofloxacin hydrochloride 400 mg capsule (366.7 mg as zabofloxacin) with zabofloxacin aspartate 488 mg tablet (366.5 mg as zabofloxacin).	AEs: two cases (7%) of nausea and a single case (3%) of somnolence, hypotension, presyncope, and increased blood creatine phosphokinase.	[242]
	Multicenter, double-blind, double-dummy, randomized, controlled, phase 3, non-inferiority trial: oral zabofloxacin (367 mg once daily for five days) versus oral moxifloxacin (400 mg once daily for seven days) in patients with COPD exacerbation.	AEs occurred in 9.7% (17/175; 25 events) of patients in the zabofloxacin group and in 9.6% (16/167; 23 events) of patients in the moxifloxacin group. There was no incidence of severe adverse drug reactions. The dropout rate due to adverse events was 0% (0/175) in the zabofloxacin group and 1.8% (3/167) in the moxifloxacin group. AEs: diarrhoea (zabofloxacin: 2.3%, moxifloxacin: 0.6%), nausea (zabofloxacin: 1.7%, moxifloxacin: 2.4%), dizziness (zabofloxacin: 1.7%, moxifloxacin: 0.6%), and chest discomfort (zabofloxacin: 1.1%, moxifloxacin: 0%).	[243]
